# Mercury in Neotropical birds: a synthesis and prospectus on 13 years of exposure data

**DOI:** 10.1007/s10646-023-02706-y

**Published:** 2023-10-31

**Authors:** Christopher J. Sayers, David C. Evers, Viviana Ruiz-Gutierrez, Evan Adams, Claudia M. Vega, Jessica N. Pisconte, Vania Tejeda, Kevin Regan, Oksana P. Lane, Abidas A. Ash, Reynold Cal, Stevan Reneau, Wilber Martínez, Gilroy Welch, Kayla Hartwell, Mario Teul, David Tzul, Wayne J. Arendt, Marvin A. Tórrez, Mrinalini Watsa, Gideon Erkenswick, Caroline E. Moore, Jacqueline Gerson, Victor Sánchez, Raúl Pérez Purizaca, Helen Yurek, Mark E. H. Burton, Peggy L. Shrum, Sebastian Tabares-Segovia, Korik Vargas, Finola F. Fogarty, Mathieu R. Charette, Ari E. Martínez, Emily S. Bernhardt, Robert J. Taylor, Timothy H. Tear, Luis E. Fernandez

**Affiliations:** 1 Department of Ecology and Evolutionary Biology, University of California, Los Angeles, CA 90095 USA; 2 Center for Mercury Studies, Biodiversity Research Institute, 276 Canco Road, Portland, ME 04103 USA; 3 Centro de Innovación Científica Amazónica, Puerto Maldonado, Madre de Dios, 17000 Peru; 4 Cornell Lab of Ornithology, 159 Sapsucker Woods Road, Ithaca, NY 14850 USA; 5 Department of Biology, Center for Energy, Environment and Sustainability, Wake Forest University, Winston-Salem, NC 27106 USA; 6 Environmental Research Institute, University of Belize, Price Center Road, P.O. Box 340, Belmopan, Cayo District Belize; 7 Foundation for Wildlife Conservation, Tropical Education Center, 28 George Price Highway, P.O. Box 368, La Democracia, Belize District Belize; 8 International Institute of Tropical Forestry, USDA Forest Service, 1201 Calle Ceiba, Jardín Botánico Sur, San Juan, 00926-1119 Puerto Rico; 9 Instituto Interdisciplinario de Ciencias Naturales, Universidad Centroamericana, Managua, Nicaragua; 10 Beckman Center for Conservation Research, San Diego Zoo Wildlife Alliance, P.O. Box 120551, San Diego, CA 92112 USA; 11 Field Projects International, Escondido, CA 92029 USA; 12 Department of Earth & Environmental Sciences, Michigan State University, East Lansing, MI 48824 USA; 13 Instituto de Investigación en Ecología y Conservación, Trujillo, Peru; 14 Universidad Nacional de Piura, Urb. Miraflores S/N, Castilla, 20002 Piura, Peru; 15 Department of Fisheries and Wildlife Biology, Clemson University, Clemson, SC 29634 USA; 16 Departamento de Biología, Universidad Nacional de Colombia, Bogotá, 111321 Colombia; 17 Department of Zoology, Faculty of Science, University of British Columbia, Vancouver, BC Canada; 18 Toucan Ridge Ecology and Education Society, 27.5 Miles Hummingbird Hwy, Stann Creek, Belize; 19 Department of Ecology & Evolutionary Biology, University of California, Santa Cruz, CA 95064 USA; 20 Department of Biology, Duke University, Durham, NC 27708 USA; 21 Department of Veterinary Medicine & Biomedical Sciences, Texas A&M University, College Station, TX 77843 USA; 22 Department of Global Ecology, Carnegie Institution for Science, Stanford, CA 94305 USA

**Keywords:** Mercury, Birds, Neotropics, Artisanal and small-scale gold mining, Bioaccumulation

## Abstract

Environmental mercury (Hg) contamination of the global tropics outpaces our understanding of its consequences for biodiversity. Knowledge gaps of pollution exposure could obscure conservation threats in the Neotropics: a region that supports over half of the world’s species, but faces ongoing land-use change and Hg emission via artisanal and small-scale gold mining (ASGM). Due to their global distribution and sensitivity to pollution, birds provide a valuable opportunity as bioindicators to assess how accelerating Hg emissions impact an ecosystem’s ability to support biodiversity, and ultimately, global health. We present the largest database on Neotropical bird Hg concentrations (*n* = 2316) and establish exposure baselines for 322 bird species spanning nine countries across Central America, South America, and the West Indies. Patterns of avian Hg exposure in the Neotropics broadly align with those in temperate regions: consistent bioaccumulation across functional groups and high spatiotemporal variation. Bird species occupying higher trophic positions and aquatic habitats exhibited elevated Hg concentrations that have been previously associated with reductions in reproductive success. Notably, bird Hg concentrations were over four times higher at sites impacted by ASGM activities and differed by season for certain trophic niches. We developed this synthesis via a collaborative research network, the Tropical Research for Avian Conservation and Ecotoxicology (TRACE) Initiative, which exemplifies inclusive, equitable, and international data-sharing. While our findings signal an urgent need to assess sampling biases, mechanisms, and consequences of Hg exposure to tropical avian communities, the TRACE Initiative provides a meaningful framework to achieve such goals. Ultimately, our collective efforts support and inform local, scientific, and government entities, including Parties of the United Nations Minamata Convention on Mercury, as we continue working together to understand how Hg pollution impacts biodiversity conservation, ecosystem function, and public health in the tropics.

## Highlights

- We summarized the largest database on Neotropical bird Hg concentrations to identify major patterns of pollution exposure to terrestrial biodiversity in the tropics.
- We detected the highest Hg concentrations in carnivorous bird species, aquatic habitats, and gold mining sites.
- We showcase among the highest published Hg concentrations for songbirds (Passeriformes) in the world.
- Madre de Dios, Peru, central Belize, and Ayapel, Colombia are biological Hg hotspots, but widespread sampling is necessary throughout the Neotropics.
- Inclusive collaboration will excel the field of tropical ecotoxicology by improving the efficiency and comparability of future monitoring efforts.

## Resúmen

La contaminación ambiental por mercurio (Hg) en los trópicos supera nuestra comprensión de sus consecuencias para la biodiversidad. Los vacíos de conocimiento que existen sobre la exposición a la contaminación podrían ocultar las amenazas para la conservación en el Neotrópico: una región que alberga a más de la mitad de las especies del mundo, pero que enfrenta una continua intensificación de las emisiones de Hg y del cambio de uso del suelo por el avance de la minería de oro artesanal y de pequeña escala (MAPE). Debido a su distribución global y su sensibilidad a la contaminación, las aves brindan una oportunidad valiosa como bioindicadores para evaluar cómo las emisiones de Hg afectan la capacidad de un ecosistema para sustentar la biodiversidad y, en última instancia, la salud global. Presentamos la más grande base de datos sobre concentraciones de Hg en aves Neotropicales (*n* = 2,316) para establecer una línea base para los niveles de exposición a Hg en 322 especies de aves de nueve países de América Central, América del Sur, y el Caribe. Encontramos patrones de las concentraciones de Hg en aves de los trópicos que se asemejan a los de las regiones templadas: mostrando una bioacumulación consistente a través de grupos funcionales y una alta variación espaciotemporal. Las especies de aves que ocupan posiciones más altas en la cadena trófica y en hábitats acuáticos registraron concentraciones elevadas de Hg que podrían tener efectos negativos en su éxito reproductivo. Es importante resaltar que las concentraciones de Hg en las aves de los sitios afectados por la MAPE fueron cuatro veces más altas que las de los sitios control y además difirió por temporada para ciertos nichos tróficos. Desarrollamos esta síntesis a través de una red de investigación colaborativa, la Iniciativa de Investigación Tropical para la Conservación y Ecotoxicología Aviar (TRACE), que ejemplifica un intercambio de datos inclusivo, equitativo e internacional. Si bien nuestros hallazgos sugieren una necesidad urgente de evaluar los sesgos en el muestreo, los mecanismos, y las consecuencias de la exposición al Hg en las comunidades de aves tropicales, la Iniciativa TRACE proporciona un marco para abordar estos objetivos. Nuestro esfuerzo colectivo tiene como propósito respaldar y brindar información a las entidades locales, científicas, y gubernamentales, incluyendo las Partes de la Convención de Minamata de las Naciones Unidas sobre el Mercurio, mientras continuamos trabajando juntos para comprender cómo la contaminación por Hg en los trópicos puede afectar la salud pública, el funcionamiento de los ecosistemas, y la conservación de la biodiversidad.

**Graphical Abstract:**

**Figure:**
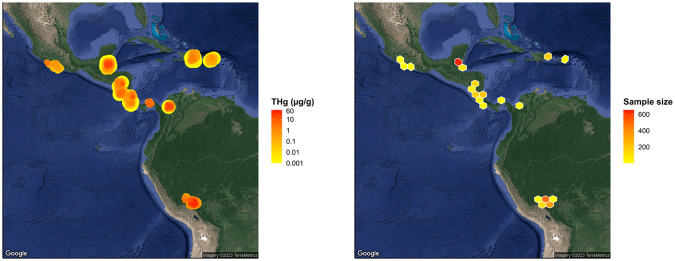

## Introduction

Environmental pollutants, including pesticides, microplastics, nutrients, and heavy metals, present profound threats to global biodiversity and health—creating a suite of unresolved challenges for health professionals, conservation biologists, landscape managers, and policy-makers (Mueller et al. 2022). Mercury (Hg) is one example of a persistent pollutant that adversely impacts environmental, animal, and public health on a global scale. Though Hg exists naturally in the environment, anthropogenic emissions from activities including resource extraction, fossil fuel combustion, metal and cement production, and waste incineration amplify environmental Hg loads (UNEP 2019). These activities can emit or release Hg directly into soils, waterways, or the atmosphere, where Hg can be mobilized around the globe and impact ecosystems far from original sources. At the local scale, microbes can convert inorganic Hg into organic methylmercury (MeHg), which is a more bioavailable form that readily biomagnifies in aquatic and terrestrial food webs (Evers et al. 2005; Cristol et al. 2008; Ackerman et al. 2016a) and can bioaccumulate to detrimental concentrations in longer-lived organisms (Evers et al. 2008; Hill et al. 2008; Scheuhammer et al. 2008; Rutkiewicz et al. 2011). For example, MeHg exposure in mammals, birds, and fish can cause behavioral, immunological, neurological, physiological, and reproductive impairment (Depew et al. 2012; Dietz et al. 2013; Scheuhammer et al. 2015; Ackerman et al. 2016a; Whitney and Cristol 2017; Evers 2018).

The World Health Organization’s “One Health” concept—an interdisciplinary approach to improve public health by mitigating threats to humans, animals, and the environment—requires robust data on global Hg emission, deposition, and exposure (WHO 2022). However, other international efforts seeking to mitigate health threats by curtailing anthropogenic Hg emissions typically have environmental data that better reflect nations in temperate, developed regions. The United Nations Minamata Convention on Mercury follows this pattern for North America (Seewagen 2010; Ackerman et al. 2016a; Cristol and Evers 2020) and Europe (Sun et al. 2019; Dietz et al. 2021), and lacks information on exposure throughout tropical regions of Africa, Asia, and Latin America (UNEP 2019). This sampling bias could undermine the goals of UN agencies, as the tropics play a crucial role in climate regulation (Sullivan et al. 2020), contribute to advances in modern medicine and biochemistry (Skirycz et al. 2016), support more than 75% of all species (Barlow et al. 2018), and will support more than half of the world’s human population by mid-century (Edelman et al. 2014). Therefore, investing in the understanding and preservation of tropical system health should be a global priority.

Human and wildlife Hg exposure in the tropics remains poorly quantified and potentially difficult to interpret due to the physical, chemical, and biological differences between Holarctic and Pantropical realms (Burger 1997; Lacher and Goldstein 1997). Many tropical regions feature three of the largest natural and anthropogenic sources of environmental Hg: geogenic (e.g., volcanism), artisanal & small-scale gold mining (ASGM), and biomass burning (Nriagu and Becker 2003; Saginor et al. 2013; Shi et al. 2019; UNEP 2019). Thus, the combination of these emissions and re-emissions has the potential to obscure source attribution and mitigation priorities to reduce organismal exposure. Relative to Holarctic forests, which have comparatively lower total leaf area, intact tropical wet forests have exceptional capacity to scavenge particulate and gaseous elemental Hg out of the atmosphere and direct it to forest floors via throughfall and litterfall (Gerson et al. 2022). Concurrently, the elevated precipitation, seasonal river fluctuations, and high wetland prevalence in tropical systems may enhance methylation rates (Burger 1997; Lacher and Goldstein 1997), as reflected by elevated “ecosystem sensitivity” for MeHg throughout the Pantropical realm (Evers and Sunderland 2019). However, some tropical regions may have enhanced demethylation rates (Shanley et al. 2020). From a community perspective, the high species richness and narrow niche breadth create more complex food webs, which are expected to increase the biomagnification potential of tropical systems (Burger 1997; Lacher and Goldstein 1997). Due to these collective factors, the ecotoxicological methods and analyses that have been developed and refined in the Holarctic realm might lack applicability in the tropics (Lacher and Goldstein 1997)—thereby limiting our capacity to assess the drivers, distribution, and impacts of Hg at these latitudes. These fundamental gaps in understanding have been repeatedly identified in past decades (Burger 1997; Lacher and Goldstein 1997; Seewagen 2010) and are particularly concerning given accelerating anthropogenic Hg emissions in many equatorial developing countries (UNEP 2019).

Artisanal and small-scale gold mining is the largest polluting sector of environmental Hg in the world, and accounts for almost 38% of global anthropogenic Hg emissions (UNEP 2019). As a major source of income for local and national economies (Wilson et al. 2015; Schwartz et al. 2021), ASGM has expanded with increasing gold demand, price, and road connectivity (Swenson et al. 2011; Alvarez-Berríos and Aide 2015; Caballero-Espejo et al. 2018), leading to further encroachment into intact tropical forests. Following forest removal, miners use high-pressure hydraulic jets to dislodge alluvial gold deposits in riparian sediments, and then add liquid elemental Hg to amalgamate gold particles (Damonte et al. 2013). Up to 60% of Hg inputs are discarded in sediments or washed downstream via mining tailings (Maurice-Bourgoin et al. 1999, 2000), while the remainder is released into the atmosphere following amalgam burning or re-emission (AMAP/UNEP 2019). Concurrently, ASGM’s alterations to hydrological landscapes drastically amplify MeHg production (Gerson et al. 2020). The *Neotropics*, collectively Central America, South America, and the West Indies, produce 42% of global ASGM Hg emissions (UNEP 2019) while also supporting around 60% of all terrestrial biodiversity (UNEP-WCMC 2016) and 8.2% of the world’s human population (World Bank 2022). This problematic overlap presents a potential conservation threat that deserves urgent attention.

Due to their global ubiquity, reliable association to specific habitats, and relative ease of detection, capture, tracking, and identification compared to other taxa, birds stand apart as the most well-studied and cost-effective taxonomic group for monitoring terrestrial biodiversity health and ecosystem function in the tropics (Bierregaard and Lovejoy 1989; Furness and Greenwood 1993; Remsen 1994; Stotz et al. 1996; Gardner et al. 2008; Lees et al. 2014). These collective factors position birds as ideal bioindicators for both local and regional ecotoxicological monitoring efforts (Furness and Greenwood 1993; Evers et al. 2008; Ackerman et al. 2016a; Egwumah et al. 2017; Sayers et al. 2021), and appropriate foci for monitoring global threats to One Health. The diversity of trophic niche and habitat specialization within the avian clade also allows species to be strategically sampled to understand exposure pathways and biogeochemical dynamics for different spatial and temporal scales (Furness and Greenwood 1993; Evers et al. 2008; Ackerman et al. 2016a; Cristol and Evers 2020). As environmental toxicant emissions continue to increase worldwide across almost all polluting industries (UNEP 2019), the associated exposure impacts could contribute to resident and migratory bird declines throughout the Americas (Robinson 1999, 2001; Sigel et al. 2006; Latta et al. 2011; Blake and Loiselle 2015, Boyle and Sigel 2015; Stanton et al. 2018; Rosenberg et al. 2019; Stouffer et al. 2020; Sherry 2021; Pollock et al. 2022). However, as only about 5% of bird species are represented in published literature on avian Hg exposure in the Neotropics (*n* = 171; Table S1), we currently have limited capacity to assess toxicological risk throughout the most species-rich region on Earth.

This synthesis builds from decades of research on the importance of Hg monitoring throughout the Global North, which has identified persistent gaps in our understanding of how Hg impacts biodiversity throughout the Global South (Burger 1997; Lacher and Goldstein 1997; Seewagen 2010; Elliott et al. 2015; Jackson et al. 2015; Ackerman et al. 2016a; Canham et al. 2020; Cristol and Evers 2020). Here, we present and summarize the largest database on Hg exposure to Neotropical birds. Our primary goals are (1) to begin quantifying the prevalence, variation, and distribution of Hg across the Neotropics by establishing exposure baselines for representative avian taxonomy and functional traits, and (2) to develop a series of recommendations, methodologies, and research priorities for avian Hg monitoring. In undertaking these goals, we hope to support future monitoring efforts in maximizing efficiency and comparability with existing research and augment our collective understanding of how Hg impacts biodiversity conservation, ecosystem function, and ultimately, public health.

## Methods

### Sample collection

We obtained samples of Neotropical resident and migratory bird Hg concentrations from published and unpublished datasets provided by the Biodiversity Research Institute, USA; Foundation for Wildlife Conservation, Belize; USDA Forest Service, Puerto Rico; Centro de Innovación Científica Amazónica, Peru; World Wildlife Fund, Peru; Universidad Centroamericana, Nicaragua; San Diego Zoo Wildlife Alliance, USA; Field Projects International, USA; Clemson University, USA; Belize Foundation for Research & Environmental Education, Belize; Toucan Ridge Ecology & Education Society, Belize; Universidad Nacional de Colombia, Colombia; and University of California, Santa Cruz, USA. Organizations are listed in descending order of samples contributed.

To collect these samples, we conducted ground-level mist-net or Bal-Chatri trap surveys at 41 sampling sites in nine countries across Central America, South America, and the West Indies during wet and dry seasons from 2007 to 2023. We selected study locations in a variety of habitats ranging from flooded tropical evergreen forest, to arid lowland scrub, to elfin forest. A total of five sampling sites in Ayapel, Colombia and Madre de Dios, Peru were located within a 7 km radius of artisanal gold mines that were either active at the time of sampling or had last been active in the preceding 5 years. Following thorough in-person exploration and inspection of present satellite imagery, we propose that ASGM emissions had a negligible direct influence on avian Hg exposure at all remaining sites, which were located upstream and at least 25 km from the closest mine. Site-specific capture and habitat information are featured in Table S2.

Whenever possible, we fitted all taxa, excluding hummingbirds (Trochilidae), with a uniquely-numbered aluminum leg band from the US Fish and Wildlife Service, CORBIDI (Center for Ornithology and Biodiversity, Lima, Peru), or the National Band & Tag Company (Newport, Kentucky, USA) to prevent resampling. In unique circumstances when we did not possess the proper leg bands, we assigned captured individuals and corresponding samples a unique identification number and clipped interior tail feathers to prevent short-term recapture. We then identified individuals to an appropriate species, sex, age, and molt cycle using a local bird field guide (Schulenberg et al. 2010; Garrigues and Dean 2014; Fagan and Komar 2016; Quiñones 2018) and either a calendar-based, or preferably, Wolfe-Ryder-Pyle (WRP) cycle-based age-classification system (Wolfe et al. 2010; Johnson et al. 2011; Tórrez and Arendt 2012, 2017; Pyle et al. 2016) described for Neotropical bird families in Johnson and Wolfe (2017). Depending on the priorities of each research effort, we measured and assessed birds for feather molt and wear, skull ossification, fat stores, muscle mass, wing chord, tarsometatarsus length, bill dimensions, tail length, body mass, and reproductive stage via cloacal protuberance and brood patch.

We followed tissue collection, preparation, and storage methods provided by the Biodiversity Research Institute (Evers et al. 2021). Whenever possible, we collected 30–60 µL of blood from the cutaneous ulnar vein using 75 µL heparinized capillary tubes, sealed tubes at both ends using Critocaps™ or Critoseal™, placed tubes into a plastic vacutainer, and stored samples in a cooler with ice packs. We transferred blood samples into a freezer within 8 h of collection, where they were stored below −4 ºC until laboratory analysis. For feather sampling, we collected the two outermost tail feathers, six “body” feathers (flank, breast, or back feathers, depending on project objectives) and stored feathers at ambient temperatures in paper coin envelopes or plastic Ziploc™ bags.

### Laboratory analysis

We analyzed avian tissue samples for total Hg (THg) at the Biodiversity Research Institute Toxicology Lab (Portland, Maine, USA; 81% of the cumulative samples), Laboratorio de Mercurio y Química Ambiental of Centro de Innovación Científica Amazónica (Puerto Maldonado, Madre de Dios, Peru; 15%), and Texas A&M University Trace Element Research Laboratory (College Station, Texas, USA; 4%) using a thermal decomposition and atomic absorption spectrophotometry technique with either a Milestone DMA-80 or Nippon MA-3000 direct Hg analyzer. Based on previous studies, we assumed that nearly all THg (>95%) in whole blood (Rimmer et al. 2005; Edmonds et al. 2010) and feathers (Thompson and Furness 1989) were in the MeHg form. Therefore, all tissue concentrations should approximate MeHg contamination of Neotropical avifauna. We followed United States Environmental Protection Agency (EPA) SW-846 Method 7473, “Mercury in solids and solutions by thermal decomposition, amalgamation, and atomic absorption spectrophotometry” (USEPA 1998). To ensure consistent precision and accuracy, we used quality control methods, including certified reference materials DORM-3, DORM-4, DOLT-4, DOLT-5, ERM-CE464, CRM-13, and NIST2710, which had recovery averages above 95% for both blood and feather tissues. We supplied an artificial concentration of 0.001 µg/g THg to any sample that registered below the analyzer’s lower detection limit (0.05 ng, 0.001 µg/g THg) rather than excluding data from statistical analyses (Shoari and Dubé 2018).

### Assigning functional traits

To explain variation in THg concentrations across bird individuals, we obtained information on species’ functional traits, which can be powerful tools to understand species’ extinction risks (Purvis et al. 2000), responses to land-use change (Hamer et al. 2015; Socolar and Wilcove 2019), as well as Hg bioaccumulation (Jackson et al. 2015; Ackerman et al. 2016a). We assigned species to an appropriate taxonomy, trophic niche, primary habitat association, and migratory status in R (v. 4.2.0; R Core Team 2022; R package “tidyverse”; Wickham et al. 2019). We standardized taxonomy based on the eBird/Clements Checklist of Birds of the World: v2022 (Clements et al. 2022). We assigned species to a trophic niche following Pigot et al. (2020), which was expanded from the EltonTraits 1.0 database (Wilman et al. 2014). We also assigned species to a primary habitat association and migratory status following Parker et al. (1996), which was adopted in Socolar and Wilcove (2019) to incorporate updated taxonomy. For modeling simplicity and ease of interpretation, we lumped habitat classifications into six representative categories for the Neotropical realm (see *Statistical analysis*). We refer to a “resident” as any species that occurs in the Neotropics throughout the full annual cycle and makes no seasonal migratory movements, a “partial migrant” as any species that breeds partly or fully within the Neotropical realm and changes their distribution in the nonbreeding season, and a “full migrant” as any species that occurs regularly in the Neotropics, but only as a nonbreeder (Parker et al. 1996; Stotz et al. 1996). Habitat classifications for migrant species represent primary nonbreeding habitat associations. Comprehensive habitat and migratory status descriptions are available in Stotz et al. (1996). In cases of taxonomic changes when there was no accompanying entry in Parker et al. (1996) or Pigot et al. (2020), we created a new entry in the databases using life history information present in Birds of the World (BOW 2022). If life history information was unclear or absent in Birds of the World, we deferred to the life history information present for the outdated species in Parker et al. (1996) and Pigot et al. (2020). We provide the updated Parker et al. (1996) and Pigot et al. (2020) databases for species included in this synthesis at https://github.com/csayers2/Neotropical-Bird-Hg-Synthesis. We summarize functional traits for all avian taxa sampled in this study in Table S3.

### Data transformation and filtering

For ease of interpretation, we pooled flank, breast, and back feather samples together as “body” feathers in subsequent models, summaries, and visualizations. Low et al. (2020) documented low THg variation among these feather tracks in North American resident and Neotropical migrant songbirds. However, researchers have not yet tested the assumption that body feather samples represent similar signals of MeHg body burden in Neotropical resident species. We lack the ability to attempt this comparison here because we did not sample sufficient individuals for multiple body feather categories.

In comparison to whole blood, which represents days to weeks of dietary Hg exposure, feathers from migratory species can represent Hg exposure from the capture location, previous migratory stopover sites, or their original breeding or wintering location before beginning migration depending on the sampling date and species’ molting patterns (see section *Tissue selection*). To increase our confidence that bird Hg concentrations represented dietary exposure at each sampling location, we excluded feather samples from migratory taxa in all models, summaries, and visualizations. Likewise, we excluded feather samples from all taxa in models and visualizations examining within-year temporal trends (see sections *Statistical analysis*, *Tissue selection*).

### Statistical analysis

Building from decades of research that have established the ecology of avian Hg exposure and bioaccumulation within the Holarctic realm, we constructed two candidate sets of linear mixed-effects models fit with maximum likelihood estimations to identify biotic and abiotic factors that best explained variation in Neotropical bird THg concentrations (R package “glmmTMB”; Brooks et al. 2017). We performed a natural-log transformation on THg concentrations so that the response variable approximated a Gaussian distribution. For the global functional trait model, we included tissue type (three types: whole blood, body feather, tail feather), trophic niche (seven niches: terrestrial vertivore, aquatic predator, invertivore, omnivore, nectarivore, frugivore, granivore), primary habitat association (six habitats: aquatic, lowland evergreen forest, lowland deciduous forest, montane evergreen forest, secondary forest, grassland/scrub), migratory status (three statuses: resident, partial migrant, full migrant), and ASGM presence at each sampling location within a 7 km radius (two levels: present or absent) as fixed effects, and included a nested sampling site/station term (41 sites, 57 sampling stations), a nested family/species/individual term (51 families, 322 species, 1856 individuals), and year (13 years represented from 2007 to 2023) as random effect intercepts. For the global temporal model of avian blood THg concentrations, we included a season (two seasons: wet and dry) by trophic niche interaction term as a fixed effect and kept the same random effects structure as the functional trait model. We performed model selection on all reduced model combinations for both the functional trait and temporal models based on second-order Akaike’s Information Criterion for small-sample sizes (*AIC*_*C*_; Burnham and Anderson 2002; R package “MuMIn”; Bartoń 2022). Using the models of best fit, we then performed an analysis of variance based on type II Wald chi-square tests (R package “car”; Fox and Weisberg 2019) and conducted Tukey pairwise comparisons to assess the relative importance of modeled factors (R package “emmeans”; Lenth 2022). We present all model-generated results as back-transformed predicted means and 95% confidence intervals from the model of best fit (R package “ggeffects”; Lüdecke 2018).

## Results and discussion

We present a collective database containing 2,316 THg samples collected from 17 orders, 51 families, and 322 bird species from 41 sites in nine countries across Central America, South America, and the West Indies from 2007–2023. Whole blood (*n* = 963) was the most well-represented sampling tissue, followed by tail feathers (*n* = 690) and body feathers (*n* = 663). Samples were distributed across Belize (*n* = 946; 5 sites), Peru (*n* = 676; 6 sites), Nicaragua (*n* = 261; 12 sites), Costa Rica (*n* = 136; 6 sites), Dominican Republic (*n* = 134; 4 sites), Colombia (*n* = 63; 1 site), Puerto Rico (*n* = 59; 1 site), Mexico (*n* = 24; 5 sites), and Panama (*n* = 17; 1 site). The seven most well-sampled bird families were Parulidae (New World warblers; *n* = 299), Thraupidae (tanagers; *n* = 240), Tyrannidae (tyrant flycatchers; *n* = 222), Furnariidae (ovenbirds; *n* = 216), Pipridae (manakins; *n* = 206), Thamnophilidae (antbirds; *n* = 154), and Troglodytidae (wrens; *n* = 107).

Following natural log-transformation of the response variable and visual inspection of plotted residuals, we detected a lack of residual normality via skewness in quantile-quantile plots for both global models (Zuur et al. 2009). Evidence of a poor distributional assumption signals that our models may conservatively overestimate the variance around predicted means. These modeling shortcomings could arise from haphazard sampling or high variance in Hg exposure among sites. Therefore, we advocate that future biomonitoring efforts pursue standardized and thorough sampling designs to better model avian Hg exposure (see *Spatial variation*, *Temporal variation*). Despite these shortcomings, other evidence suggests that our models remain statistically robust. Our top-performing functional trait and temporal model accounted for ≥92% of variation in the data, ≥68% of total model weight, and were statistically distinguishable (*∆AIC*_*C*_ > 2) from all other models within their respective candidate sets (Table S4). Therefore, we chose to forgo model averaging and base inferences and predictions off the models of best fit. Tables S4–5 summarize linear mixed-effects model selection and analysis of variance results, and Tables S8, S6–8, and Figs. S1–6 summarize arithmetic mean THg concentrations by country, site, trophic niche, primary habitat association, and taxonomy.

### Trophic niche, habitat association, and taxonomy

**Fig. 1:**
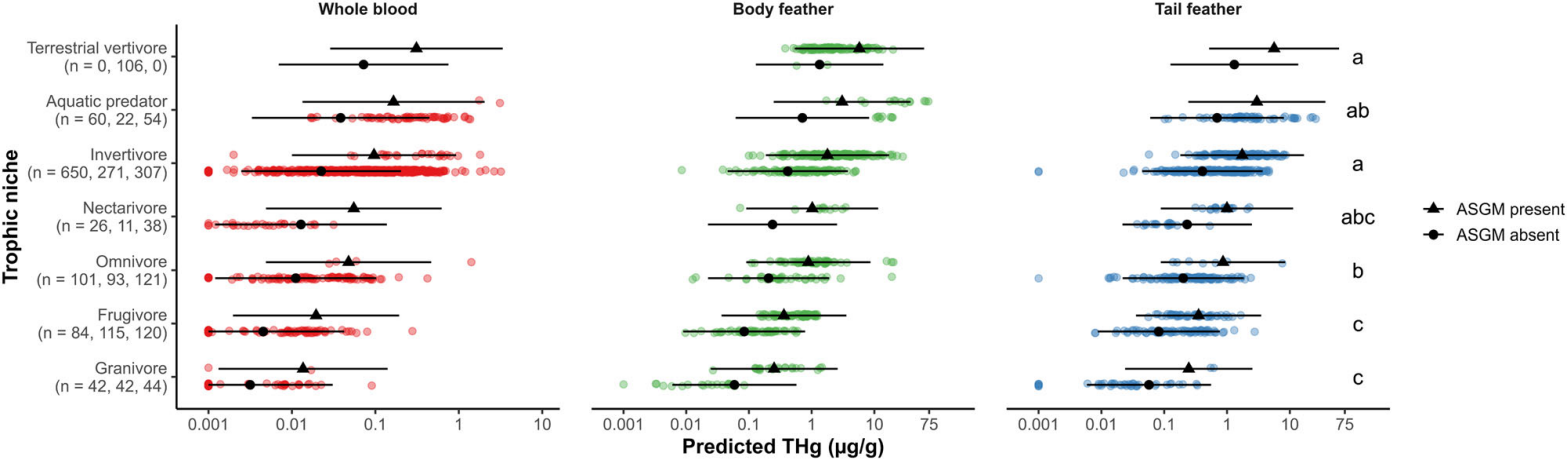
Total mercury (THg) concentrations (µg/g) overlaid with back-transformed predicted means ± 95% confidence intervals among Neotropical bird trophic niches and artisanal and small-scale gold mining (ASGM) presence. Bird THg concentrations were nearly four times higher at ASGM sites on average (*p* < 0.001). Non-overlapping letters indicate statistically significant differences (*p* < 0.05) among groups based on Tukey pairwise comparisons across tissue types

**Fig. 2:**
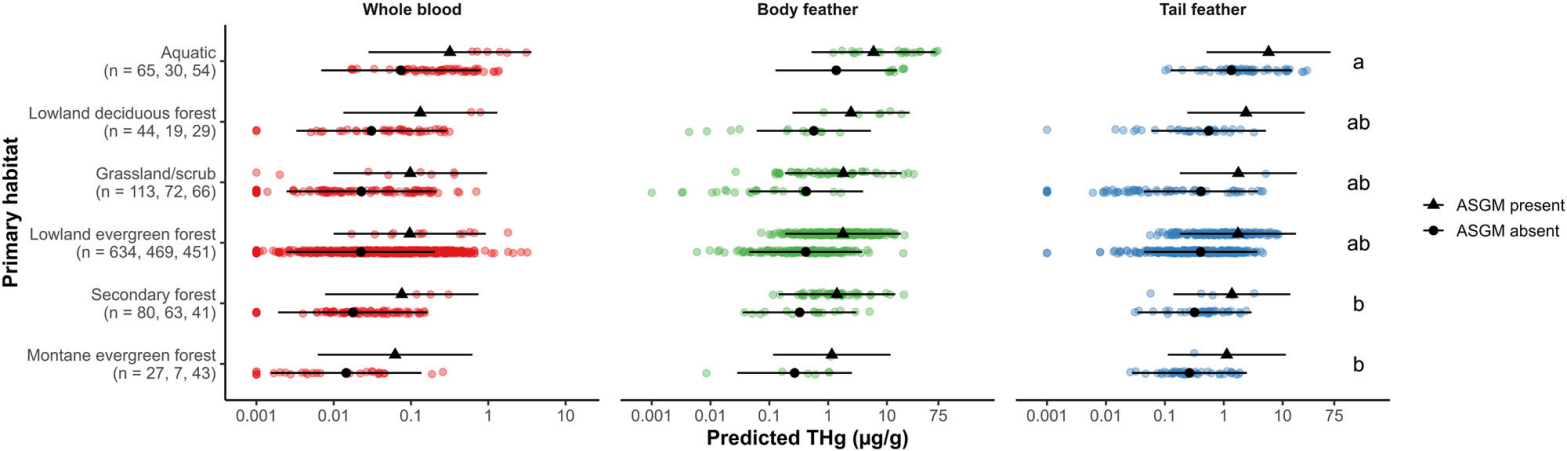
Total mercury (THg) concentrations (µg/g) overlaid with back-transformed predicted means ± 95% confidence intervals among Neotropical bird primary habitat associations and artisanal and small-scale gold mining (ASGM) presence. Bird THg concentrations were nearly four times higher at ASGM sites on average (*p* < 0.001). Non-overlapping letters indicate statistically significant differences (*p* < 0.05) among groups based on Tukey pairwise comparisons across tissue types

**Fig. 3:**
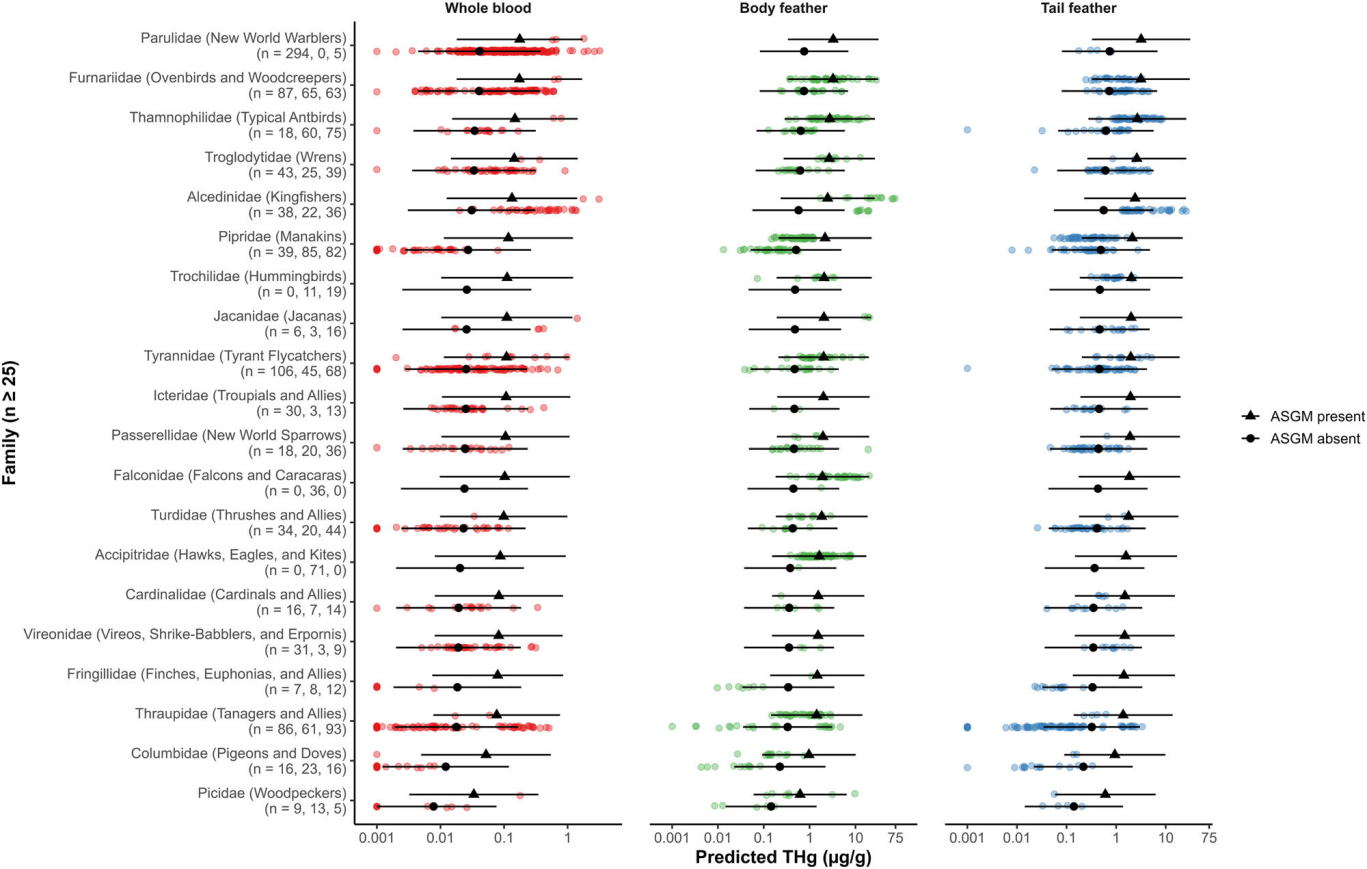
Total mercury (THg) concentrations (µg/g) overlaid with back-transformed predicted means ± 95% confidence intervals among Neotropical bird families and artisanal and small-scale gold mining (ASGM) presence. Bird THg concentrations were nearly four times higher at ASGM sites on average (*p* < 0.001). Families with fewer than 25 samples are excluded

Neotropical bird THg concentrations differed among trophic niches (*χ*^2^ = 85.59, *p* < 0.001; Figs. 1, S1; Tables S5, S6), primary habitat associations (*χ*^2^ = 14.47, *p* = 0.013; Figs. 2, S2; Tables S5, S7), and taxonomy (*χ*^2^ = 639.34, *p* < 0.001; Figs. 3, S3–5; Tables S5, S8). Terrestrial vertivores, aquatic predators, and invertivores exhibited the highest predicted and mean THg concentrations, while omnivores, frugivores, and granivores exhibited the lowest (Figs. 1, S1; Table S6). Taxonomic groups that occupy carnivorous trophic levels, especially families Parulidae (New World warblers), Furnariidae (ovenbirds), Thamnophilidae (antbirds), Troglodytidae (wrens), and Alcedinidae (kingfishers), also ranked highest in predicted and mean THg concentrations (Figs. 3, S4; Table S8). Species that prefer aquatic habitats exhibited the highest predicted and mean THg concentrations and were significantly higher than those that prefer secondary and montane evergreen forests (Figs. 2, S2; Tables S5, S7). In terms of data variability, coefficients of THg variation (CVs) were high (≥50%) in blood and feather samples for most trophic niches, primary habitat associations, orders, families, and species (Tables S6–8).

These results are consistent with avian Hg dynamics and regional syntheses within the Holarctic realm (Evers et al. 2005; Evers et al. 2011; Mallory and Braune 2012; Jackson et al. 2015, 2016; Ackerman et al. 2016a). Birds feeding at higher trophic levels and in aquatic habitats typically bioaccumulate higher THg concentrations due to MeHg biomagnification up food webs (Evers et al. 2005; Jackson et al. 2015; Ackerman et al. 2016a) and because of biogeochemical conditions that facilitate microbial methylation in aquatic environments (Ullrich et al. 2001). The high variation present within these data is also not surprising given the high spatial heterogeneity of avian Hg exposure (Evers et al. 2005; Lane et al. 2011, 2020; Ackerman et al. 2016a; Tsui et al. 2018; Brasso et al. 2020; Sayers et al. 2021), and similarly high coefficients of Hg variation when sampling over large geographic areas (Ackerman et al. 2016a; Dzielski et al. 2019).

Trophic niche pairwise comparisons indicated that invertivores, aquatic predators, and terrestrial vertivores were not significantly different in predicted THg (Fig. 1; Table S5). Avian invertivores often bioaccumulate Hg concentrations that match or exceed those in taxa occupying higher trophic positions, especially avian piscivores (Evers et al. 2005; Cristol et al. 2008; Jackson et al. 2015; Ackerman et al. 2016a; Abeysinghe et al. 2017; Adams et al. 2020). This is one of the first studies based in the Neotropical realm to support this relationship and identify a variety of invertivorous taxa that experience Hg exposure comparable to taxa that predate fish and other vertebrates. We showcase repeated instances of species in Parulidae (New World warblers), Furnariidae (ovenbirds), Thamnophilidae (antbirds), Formicariidae (antthrushes), and Troglodytidae (wrens) that exceeded terrestrial vertivores in Falconidae (falcons and caracaras), Accipitridae (hawks, eagles, and kites), and Strigidae (owls), as well as aquatic predators in Alcedinidae (kingfishers), Ardeidae (herons), and Sulidae (boobies and gannets), in predicted and mean THg concentrations (Fig. S5; Table S8). These observations may be attributable to a rich diet of arachnids for some species, in which the predatory behavior and elevated biomagnification potential of spiders can increase avian Hg bioaccumulation (Cristol et al. 2008; Chumchal et al. 2022). Our findings have potentially large implications for the Pantropical realm considering that invertivores constitute more than 60% of all tropical bird species (Sherry 2021). However, we must also acknowledge that aquatic predators including *Chloroceryle americana* (green kingfisher; 35.75 ± 27.92 µg/g fw body feather), *Chloroceryle amazona* (Amazon kingfisher; 23.90 ± 5.85 µg/g fw body feather), and *Chloroceryle aenea* (American pygmy kingfisher; 10.31 ± 7.73 µg/g fw tail feather; 0.54 ± 0.40 µg/g ww whole blood) exhibited the highest mean THg concentrations across all tissue types due to a combination of high trophic position and ASGM emissions (see *Artisanal and Small-scale Gold Mining (ASGM)*; Fig. S5; Table S8).

Notably, nectarivores were not statistically different from all higher trophic niches in predicted THg, in which Trochilidae (hummingbirds) ranked seventh highest among families with 25 or more samples (Figs. 1, 3; Table S5). This is likely a biased signal because all hummingbird samples were collected from ASGM sites in Madre de Dios, Peru. However, this comparison does illuminate the high bioaccumulation potential of hummingbirds and how invertivorous diets (e.g., predating spider webs) may influence their Hg exposure (Remsen et al. 1986; Stiles 1995; Guevara and Stiles 2005; Hardesty 2008; Mikoni et al. 2017).

Overall, our findings showcase consistent patterns in avian Hg exposure between Holarctic and Neotropical realms. Further, they assert that Neotropical invertivores regularly match or even exceed THg concentrations in taxa occupying higher trophic positions and identify regions and taxonomic groups that deserve additional focus (see *Spatial variation*, *Sentinel species selection*).

### Spatial variation

Neotropical bird THg concentrations also differed among sampling sites (*χ*^2^ = 321.52, *p* < 0.001; Figs. S6, 7; Tables S2, S5), which aligns well with previous predictions about the high spatial heterogeneity of the Neotropics (Burger 1997; Lacher and Goldstein 1997), as well as with site-specific Hg variation across the Holarctic realm (Evers et al. 2005; Lane et al. 2011, 2020; Ackerman et al. 2016a; Sayers et al. 2021). When comparing among the most well-sampled sites (*n* ≥ 25), central Belize, Ayapel, Colombia, and Madre de Dios, Peru exhibited consistently high predicted and mean THg concentrations—signaling that these regions are biological Hg hotspots (Figs. S6–7; Table S2). While there is still much to investigate here, bird communities in Belize may have elevated THg concentrations because of gaseous elemental Hg emissions from local landfill incineration, coal combustion in central Mexico (UNEP 2019), or industrial and artisanal gold mining in the Chiquibul/Maya Mountains (Cornec 2010; Briggs et al. 2013; Manzanero 2014; Rath 2016). In addition, there is an abundance of Hg methylating habitats at some Belize sampling sites, including seasonal wetlands, that may convert inorganic emissions to a more bioavailable form. We principally attribute elevated bird THg concentrations in Ayapel, Colombia, and Madre de Dios, Peru to ASGM activities. All three of our sampling stations in Ayapel feature freshwater wetlands directly downstream from active gold mining operations. This situation creates theoretically ideal conditions for high MeHg production: elevated volumes of aqueous inorganic Hg that interact with organic sediment via periodic inundation. Madre de Dios is now recognized as a global hotspot for ASGM, where this often-illicit industry consumes up to 10,000 ha of primary rainforest and releases up to 185 Mg of inorganic Hg into local waterways per year (Andina 2015; Asner and Tupayachi 2017; Collyns 2019; Caballero-Espejo et al. 2018). Although the Peruvian government augmented their enforcement of illegal gold mining in certain areas of Madre de Dios in 2019–2020 (e.g., Operación Mercurio 2019; Leas 2019), ASGM continues unrestrained in other parts of the region. Further, legacy Hg released from over five decades of ASGM activity in Madre de Dios persists in aquatic and terrestrial systems (Diringer et al. 2015).

### Artisanal and Small-scale Gold Mining (ASGM)

Perhaps the most important finding of this study is that bird THg concentrations were over four times higher at sites within 7 km of artisanal gold mining activities than at other sites across the Neotropics (*χ*^2^ = 20.54, *p* < 0.001; Figs. 1–3; Tables S2, S5). These ASGM-impacted sites not only featured among the highest Hg concentrations ever published for songbirds (Passeriformes; Abeysinghe et al. 2017; Evers et al. 2023) but also kingfisher samples that set a new record for the highest mean feather Hg concentration ever reported for a bird species in South America (*Chloroceryle americana*: 35.75 ± 27.92 µg/g fw body feather; Balza et al. 2021).

These results represent a wake-up call for tropical bird conservation, and signal an urgent need to assess the community-level consequences of this industry by further clarifying the scale and endpoints of impact, as well as the biomagnification mechanisms at play. Our model estimates should neutralize much of the doubt surrounding the role of ASGM in contributing to elevated Hg exposure for tropical biodiversity. As many tropical regions are geochemically active and face a surge of biomass burning via agricultural expansion, there has been perpetual uncertainty and scrutiny about whether natural Hg emissions and re-emissions are responsible for high organismal exposure. While we cannot adequately determine source attribution without the use of stable Hg isotopes, an evolving methodology for birds (Kumar et al. 2018; Tsui et al. 2018; Li et al. 2022), we assert that it is negligent to disregard ASGM as one of the principal drivers of Hg exposure in the Neotropics given that (1) global anthropogenic emissions vastly exceed natural ones, and (2) ASGM contributes the majority of Hg emissions across all anthropogenic sectors (UNEP 2019). Regardless of the theoretical presence of naturally-sourced Hg at ASGM sites, our model estimates demonstrate a clear additive effect of ASGM emissions compared to background concentrations.

### Temporal variation

Neotropical bird THg concentrations tended to be higher in dry seasons for many trophic niches, but there was not a significant seasonal difference at the community level in our top-performing model (*χ*^2^ = 3.65, *p* = 0.056; Table S5). Including a season by trophic niche interaction term vastly improved model fit, indicating that certain trophic niches experienced significant differences in exposure between dry and wet seasons (*χ*^2^ = 41.19, *p* < 0.001; Fig. S8; Table S5). Hg concentrations also differed among years (*χ*^2^ = 103.67, *p* < 0.001; Table S5), but due to our inconsistent sampling across locations throughout the full annual cycle, we are unable to determine the directionality of these trends. Intra- and inter-annual temporal patterns are likely to be most biologically meaningful for taxonomic groups occupying higher trophic niches and populations occupying Hg hotspots.

Intra-annual mechanisms, such as seasonal re-emission and methylation rates or even dietary shifts throughout the annual cycle, could contribute to seasonal differences in avian Hg exposure. Tropical precipitation rates change dramatically throughout the annual cycle, leading to distinct dry and wet seasons, often with accompanying pulses in biomass burning and flooding, respectively. These intra-annual changes likely contribute to strong seasonal atmospheric Hg trends in tropical South America, though further research is valuable (Koenig et al. 2021; Gerson et al. 2022). Due to the substantial sequestration of gaseous elemental Hg by forests, leaf litter, and soil organic matter (Obrist 2007; Jiskra et al. 2015, 2018; Obrist et al. 2018; Gerson et al. 2022), during months of relatively little precipitation, biomass burning events can re-emit large quantities of Hg to the atmosphere (Webster et al. 2016; Fraser et al. 2018; Shi et al. 2019; Koenig et al. 2021). Changing sediment moisture conditions, such as those following rainfall and flooding events, produce conditions that amplify MeHg production and bioavailability (Snodgrass et al. 2000; Hall et al. 2008). Therefore, we expect avian Hg exposure acquired through food web biomagnification to track well with seasonal atmospheric trends—peaking during the dry-wet seasonal transition following biomass burnings in the dry season (Shi et al. 2019; Koenig et al. 2021) and the release of MeHg into aquatic systems during inundation (Devito and Hill 1999; Snodgrass et al. 2000; Eimers et al. 2003; Hall et al. 2008). Concurrently, many species within omnivorous, frugivorous, and granivorous trophic niches shift to a more invertivorous diet during the breeding cycle to match the protein needs of their offspring (Moermond and Denslow 1985). Therefore, due to elevated Hg biomagnification in carnivorous food webs, we should expect bird Hg concentrations to increase during breeding seasons, which vary by taxonomy and geography.

These proximate, intra-annual mechanisms operate amidst ultimate, inter-annual mechanisms pertaining to emission, methylation, land-use, and climate changes. Anthropogenic Hg emissions have steadily increased throughout the Neotropical realm since at least 1980 (UNEP 2013, 2019; Streets et al. 2017). These trends are largely a result of ASGM emissions, which have almost doubled at 5-year increments since 2005 in South America (UNEP 2013, 2019). Artisanal and industrial Hg emissions may be compounded by (1) the increased methylation capacity of ASGM landscapes (Gerson et al. 2020), and (2) increasing biomass burning re-emissions via agricultural expansion and evapotranspiration feedback loops (Nature 2019; Escobar 2020). ASGM activities convert forests to aquatic habitats, especially ponds, which can dramatically increase MeHg production (Gerson et al. 2020). Cattle ranching and soybean production, which regularly utilize slash-and-burn land clearing methods, are the principal drivers of deforestation and biomass burning in the Neotropics (Sampaio et al. 2007; Nature 2019; Lapola et al. 2023). Specifically in Amazonia, there is now mounting evidence that forest cover loss reduces evapotranspiration and precipitation recycling, leads to an altered hydrological cycle with an extended dry season, increases the frequency and intensity of biomass burnings and subsequent re-emissions of gaseous elemental Hg, and further reduces forest cover (Sampaio et al. 2007; Gloor et al. 2015; Lovejoy and Nobre 2018; Peña-Claros and Nobre 2023)—a positive feedback loop known as savannification. Anecdotally, the management actions necessary to bring the Amazon back from this tipping point (Lovejoy and Nobre 2018; Peña-Claros and Nobre 2023), such as implementing sustainable agriculture practices to curb deforestation and increase soil carbon stocks (Loker 1994; zu Ermgassen et al. 2018; Ogle et al. 2019), and mending fragmented habitats through ecosystem restoration (Aide et al. 2000; Strassburg et al. 2020), all have the potential to greatly decrease Hg re-emissions, while protecting the health and longevity of humans and biodiversity—a win-win-win situation.

### Migratory strategy

Migratory status as a fixed effect was excluded from the top-performing model during model selection and Neotropical bird Hg concentrations did not differ among migratory statuses in the global model (*χ*^2^ = 1.77, *p* = 0.412; Table S2). However, Hg biomonitoring efforts should continue to sample Neotropical migrants in parallel with resident species since there are several toxic mechanisms that could influence their migratory success (Seewagen 2020).

New World warblers and tyrant flycatchers are among the highest-ranking Neotropical families in predicted and mean THg concentrations and have declined over 25% and 20% in North America, respectively (Rosenberg et al. 2019). As these species must overcome immense physical challenges to complete a successful migration, this life history stage is commonly associated with high mortality (Sillett and Holmes 2002; Paxton et al. 2017; Rushing et al. 2017). Hg exposure throughout the full annual cycle (Evers 2018; Cristol and Evers 2020) is widely expected to introduce additional complications for navigation, flight endurance, stopover refueling, cell oxidation, and predator avoidance along the migratory route (Ma et al. 2018a, b; Adams et al. 2020; Seewagen 2020), which could contribute to seasonal carry-over effects and regional population declines (Ma et al. 2018a).

Methylmercury has the potential to influence migratory success via acute and chronic mechanisms of toxicity. Ma et al. (2018b) showed that *Setophaga coronata* (yellow-rumped warblers) rapidly accumulated dietary MeHg into the bloodstream and incurred more frequent strikes and decreased flight endurance during wind tunnel experiments as a consequence of acute neurotoxicity. One chronic toxicity mechanism that can impact migratory success is fluctuating asymmetry, in which feather growth departs from bilateral symmetry on the wings and tail. In *Sterna forsteri* (Forster’s terns) and *Gavia immer* (common loons), fluctuating asymmetry was positively correlated with blood and feather Hg concentrations (Evers et al. 2008; Herring et al. 2017) and has been shown to reduce take off speed, aerial maneuverability, and flight performance in *Sturnus vulgaris* (European starlings; Swaddle et al. 1996). Birds accommodating for imperfections in drag, wing shape, and weight distribution due to clipped or naturally molting feathers, generate less mechanical power, have slower flight speeds, and exert more energy during flight (Thomas 1993; Chai 1997; Chai and Dudley 1999; Hambly et al. 2004). This increased energy expenditure has profound implications for migratory success. Over long distances, and especially trans-oceanic flights, Hg-driven asymmetry could therefore result in shorter flight durations, lengthened stopover time, delayed arrival to favored breeding territories (Seewagen 2020), reduced predator avoidance abilities (Lind et al. 2010), or even distance misjudgments, starvation, and fatigue.

Considering these potential mechanisms, Ma et al. (2018a) introduced and supported a fundamental hypothesis in which long-distance Neotropical migrant songbirds departing from the breeding grounds during autumn migration with high Hg exposure are less likely to return in the following spring. As a result, scientists would observe a higher frequency of migrants with lower Hg concentrations during spring migration—with the main implication being that, due to a Hg-driven reduction in migration success, there are fewer breeding migrants as time progresses. However, this hypothesis ignores Hg exposure present on the nonbreeding grounds. Framing Hg-migration dynamics only in terms of breeding ground exposure is problematic because it removes any culpability that migrant wintering grounds can contribute to reductions in spring migration success, and reduces the impetus for ecotoxicological biomonitoring in tropical regions. Using the Ma et al. (2018a) hypothesis, we would also predict that Neotropical migrants sampled on the wintering grounds with the highest Hg concentrations would be the least likely to complete a successful spring migration. And if this prediction is valid, we would also observe a higher frequency of migrants with lower Hg concentrations during spring in North America. To elucidate the effects of Hg on Neotropical migrants and the role that Hg may have in regional migratory bird declines (Rosenberg et al. 2019), ecotoxicologists should implement strategic tissue sampling of this clade during all life stages (Jackson et al. 2015).

### Risk assessment

**Fig. 4:**
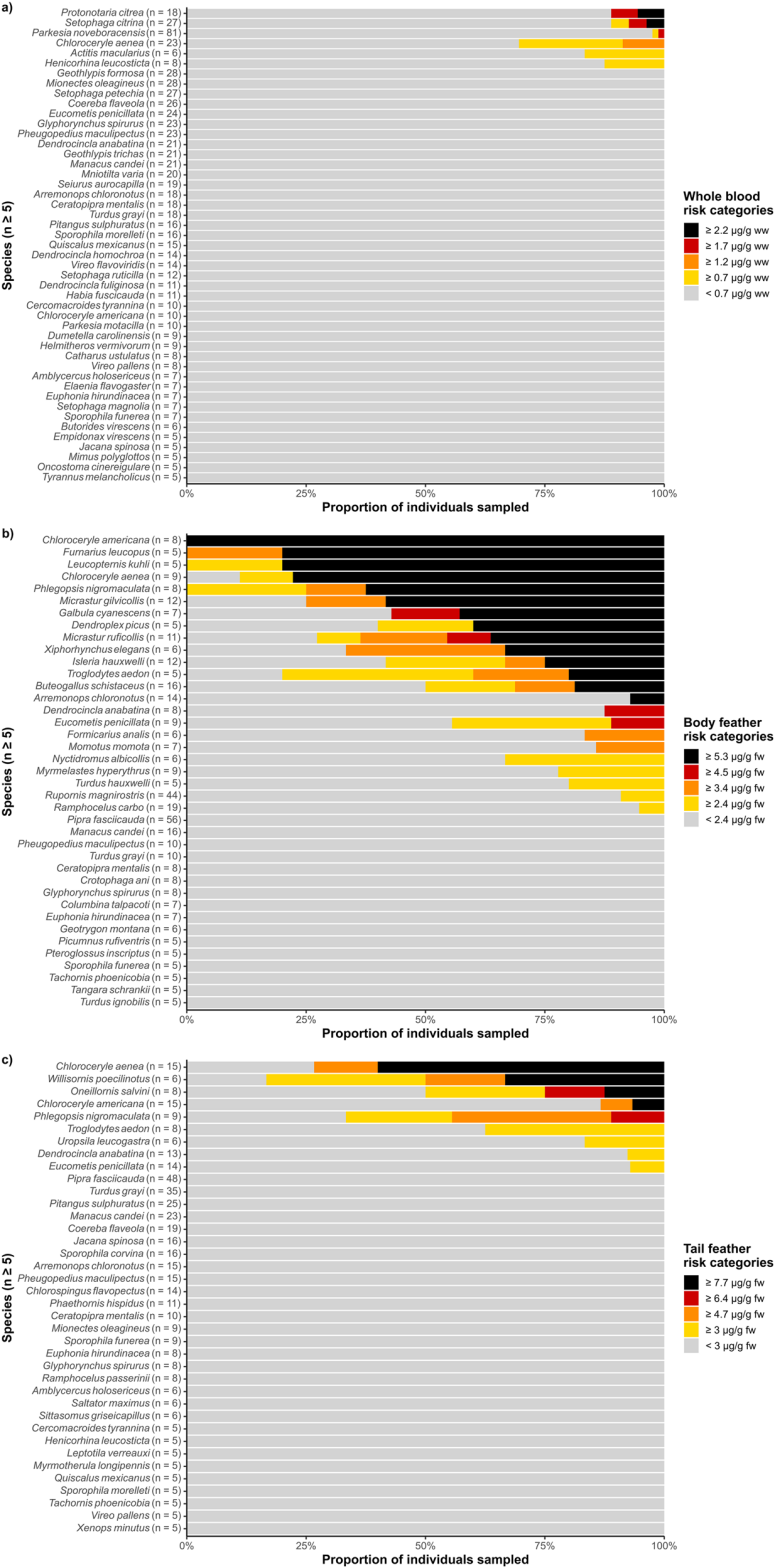
Proportion of Neotropical bird species sampled for (**a**) whole blood, (**b**) body feathers, and (**c**) tail feathers that may be subject to reductions in reproductive success via MeHg exposure, as defined by Jackson et al. (2011). Species with fewer than five samples are excluded. Whole blood risk categories are: < 0.7 µg/g ww (gray, ≤10% decline in reproductive success), ≥0.7 µg/g ww (yellow, ≥10%), ≥1.2 µg/g ww (orange, ≥20%), ≥1.7 µg/g ww (red, ≥30%), and ≥2.2 µg/g ww (black, ≥40%). Body feather risk categories are: < 2.4 µg/g ww (gray, ≤10%), ≥2.4 µg/g ww (yellow, ≥10%), ≥3.4 µg/g ww (orange, ≥20%), ≥4.5 µg/g ww (red, ≥30%), and ≥5.3 µg/g ww (black, ≥40%). Tail feather risk categories are: <3 µg/g ww (gray, ≤10%), ≥3 µg/g ww (yellow, ≥10%), ≥4.7 µg/g ww (orange, ≥20%), ≥6.4 µg/g ww (red, ≥30%), and ≥7.7 µg/g ww (black, ≥40%)

Using established effect concentrations featured in Jackson et al. (2011), 9.5% (221/2316) of individuals across 26% (85/322) of species and 45% (23/51) of families within our sampled community may experience a 10% or greater reduction in reproductive success. At the species level, we estimate that a total of five terrestrial vertivore, three aquatic predators, 19 invertivores, and four omnivore species with more than five samples in a given tissue category fall above this risk threshold (Fig. 4). Most at-risk species were among the highest-ranking families in predicted and mean THg concentrations: Alcedinidae (kingfishers), Thamnophilidae (antbirds), Furnariidae (ovenbirds), Parulidae (New World warblers), and Troglodytidae (wrens; Figs. 3, S4). In addition, most at-risk individuals were sampled from Hg hotspots that we identified: central Belize, Ayapel, Colombia, and Madre de Dios, Peru.

Because we collected over 95% of bird tissue samples via ground-level mist-net surveys, our results and subsequent risk assessments are applicable to strata generalists and understory species. This is particularly valuable considering the declines of understory invertivores within protected Neotropical forest tracts (Robinson 1999, 2001; Sigel et al. 2006; Latta et al. 2011; Blake and Loiselle 2015; Boyle and Sigel 2015; Stouffer et al. 2020; Sherry 2021; Pollock et al. 2022). Several species with mean THg concentrations that exceeded sublethal effect thresholds, including *Formicarius analis* (black-faced antthrush), *Willisornis poecilinotus* (common scale-backed antbird), *Dendrocincla fuliginosa* (plain-brown woodcreeper), *Dendrocincla homochroa* (ruddy woodcreeper), and *Myrmotherula axillaris* (white-flanked antwren), also suffered significant abundance declines in recent studies on Neotropical avifaunal collapse (Stouffer et al. 2020; Pollock et al. 2022; Table S8). Given these risk estimates and overall elevated concentrations for these taxa, we posit that ASGM and other Hg-polluting industries throughout Latin America may play a role in shaping the understory invertivore community. However, we acknowledge that toxicity reference values developed for Holarctic species may be limiting when assessing risk for Neotropical resident avifauna (Canham et al. 2020).

Selecting Hg effect thresholds that can be applied to the 322 bird species included in this synthesis is a difficult task. While toxicity reference values have been defined for a variety of endpoints, relatively few bird species have been studied, and none exist for tropical biomes (Fuchsman et al. 2017; Cristol and Evers 2020). Researchers have often relied on extrapolation across species, families, or even orders of similar diet or body size when reference values have not been defined for a species of interest (Warner et al. 2010; Lane et al. 2011, 2020; Winder 2012; Sayers et al. 2021). Because species vary widely in their respective sensitivities to Hg (Fuchsman et al. 2017; Cristol and Evers 2020), extrapolation has the potential to be inaccurate (USEPA 2007), and to ignore interspecific differences in size, trophic position, and evolved Hg tolerance (Thompson and Furness 1989; Eagles-Smith et al. 2009; Fuchsman et al. 2017).

Neotropical species may have higher or lower tolerance to Hg exposure than Holarctic species due to a variety of environmental and evolutionary factors. As the Neotropics are a geochemically-active region, resident bird communities could exhibit elevated tolerance to MeHg bioaccumulation via generations of natural exposure from volcanism and leaching of volcanic rock (Nriagu and Becker 2003; Saginor et al. 2013). Neotropical birds tend to have longer molt cycles than Holarctic species (Moreno-Palacios et al. 2018), which provides more time to depurate Hg into developing feathers and reduce their body burden. Neotropical birds also tend to be larger, longer-lived, and have lower metabolic rates than Holarctic species; which, in addition to differentiated cellular properties, appears to increase their resistance to oxidative stress (Jimenez et al. 2014). Therefore, Neotropical bird species may be able to tolerate higher concentrations of Hg (Canham et al. 2020). On the contrary, the slower “pace of life” and metabolism of Neotropical species (Wikelski et al. 2003; Jimenez et al. 2014) could reduce their capacity to eliminate Hg through excretion, depuration, or demethylation. Neotropical species tend to have longer breeding periods with smaller clutch sizes, sometimes lasting all year, have a lower annual reproductive output, and exist at lower densities than Holarctic species (Jetz et al. 2008; Jimenez et al. 2014; but see Arendt 2004, 2006). Therefore, Hg exposure may have a disproportionately negative impact on Neotropical bird population growth, stability, and recovery (Burger 1997). Despite these broad-scale differences between Neotropical and Holarctic taxa, it is important to attempt to provide context for our results. Therefore, as we lack any evidence of how Hg may influence Neotropical bird species, we cautiously extrapolate from frequently-cited toxicity reference values for songbirds in North America.

Jackson et al. (2011) monitored *Thryothorus ludovicianus* (Carolina wrens), a non-migratory invertivorous songbird, breeding along two contaminated rivers in Virginia, USA, to develop effect concentrations based on percent reductions in nesting success. Females with blood, body feather, and tail feather THg concentrations of 0.7, 2.4, and 3.0 µg/g, respectively, were projected to experience a 10% reduction in nesting success. These toxicity reference values have since been routinely used to provide context for a variety of invertivorous passerine species (Winder 2012; Evers 2018; Lane et al. 2020; Sayers et al. 2021). While non-passerine species, including members of Alcedinidae (kingfishers), Falconidae (falcons), Strigidae (owls), and Momotidae (motmots), tend to be larger and consume higher trophic level prey items than invertivorous songbirds, McNab (1988) notes that vertebrate-eating birds weighing less than a kilogram, including small raptors (Order Accipitriformes and Falconiformes), seabirds (Order Procellariiformes), and members of family Alcedinidae (kingfishers), have high basal metabolic rates similar to invertivorous passerines. Because avian Hg exposure and toxicokinetics are related to body mass, birds of similar size and metabolic rate should have similar sensitivities to Hg (Fuchsman et al. 2017). Therefore, we have increased confidence in applying toxicity reference values from Jackson et al. (2011) to non-passerine species present in our database.

## Research priorities & recommendations for future biomonitoring

A plethora of additional research is necessary to address important and persistent gaps in our understanding of Hg exposure to Neotropical birds. We assert that the most immediate priority for the field of Neotropical ecotoxicology is to clarify how Hg exposure and risk changes over time and space, which not only requires interdisciplinary expertize and coordination, but also systematic sampling of sites, habitats, taxonomy, and tissues.

A critical, albeit challenging, task to overcome the present limitations of interpreting Hg exposure to Neotropical birds is to develop effect concentrations for reproductive endpoints using field-based approaches. These toxicity thresholds should reflect the most abundant and widespread sentinel species to broaden their taxonomic and geographic applicability (see *Sentinel species selection*; Table S9). Metrics of reproductive success (e.g., nesting attempts, clutch size, nestling survival, proportion of chicks fledged) are the most useful interpretive endpoints for ecological Hg effects because they can be relatively easy to measure in cavity-nesting species and have direct population-level consequences (Brasso and Cristol 2008; Evers et al. 2008; Jackson et al. 2011). In controlled laboratory-based, or ex situ, studies that rely on the dosing of captive individuals, reproductive success is easily isolated and quantifiable without the influence of stochastic environmental stressors, including food abundance, weather, predation, or human interference. However, the lack of stochasticity creates a potentially large limitation in applying ex situ adverse-effect thresholds to in situ free-living populations, which must compensate for Hg contamination in the face of environmental stress. Therefore, laboratory-based studies should supplement empirical field-based studies to characterize the role of environmental stochasticity, define toxicokinetic relationships, and better explain in situ responses. By integrating these efforts, we can begin to provide more-accurate estimations of Hg risk in past, present, and future avian populations.

A robust way to examine temporal patterns in avian Hg exposure is through the simultaneous monitoring and comparison of contemporary and historical bird communities. To better understand proximate, intra-annual mechanisms, we recommend the consistent collection of avian blood samples—a tissue representative of recent dietary Hg exposure (see *Tissue selection*)—across sites throughout the full annual cycle. To define ultimate, inter-annual mechanisms, however, museum collections provide enormous power to understand past, present, and future Hg emissions scenarios for biodiversity (Vo et al. 2011; Evers et al. 2014; Perkins et al. 2020). By leveraging the chemical stability of MeHg in feathers (Applequist et al. 1984) and evolving methods to quantify stable Hg isotope ratios (Kumar et al. 2018; Tsui et al. 2018; Li et al. 2022), we can analyze preserved bird specimens in natural history museums to understand how emission sources, biogeochemical cycling, and biological risk of Hg pollution have changed over time.

Ecotoxicologists should continue to monitor the Hg hotspots we identify here, as they provide the opportunity to: (1) further understand inherent differences between Holarctic and tropical Hg dynamics, (2) inform government agencies about the ecological impacts of gold mining activities, (3) refine the selection of sentinel species for Neotropical habitats and regions, (4) examine temporal changes in Hg emission and methylation rates, and (5) serve as reference sites for future comparisons. In particular, weighing the rapid expansion of ASGM in Neotropical lowland ecosystems with the global significance and general lack of Hg exposure data for these regions, the Amazon River Basin should be one of the top sampling priorities for Neotropical ecotoxicologists throughout the next decade (Canham et al. 2020). Coupled with immense inorganic Hg emissions at small-scale operations, mining can contribute 9–70% of local annual deforestation in Amazonia (Sonter et al. 2017; Caballero-Espejo et al. 2018). As such, there are not only ecotoxicological concerns but also significant fragmentation, biogeography, carbon sequestration, climate change, and savannification implications associated with ASGM—placing further strain on conservation efforts in the face of mining pressure (Sonter et al. 2018). Given its staggering biodiversity, relatively small size, high density of ASGM activity, and robust network of biological stations and ecolodges, we propose Madre de Dios, Peru as a meaningful starting point for long-term Hg biomonitoring efforts in Amazonia.

**Fig. 5:**
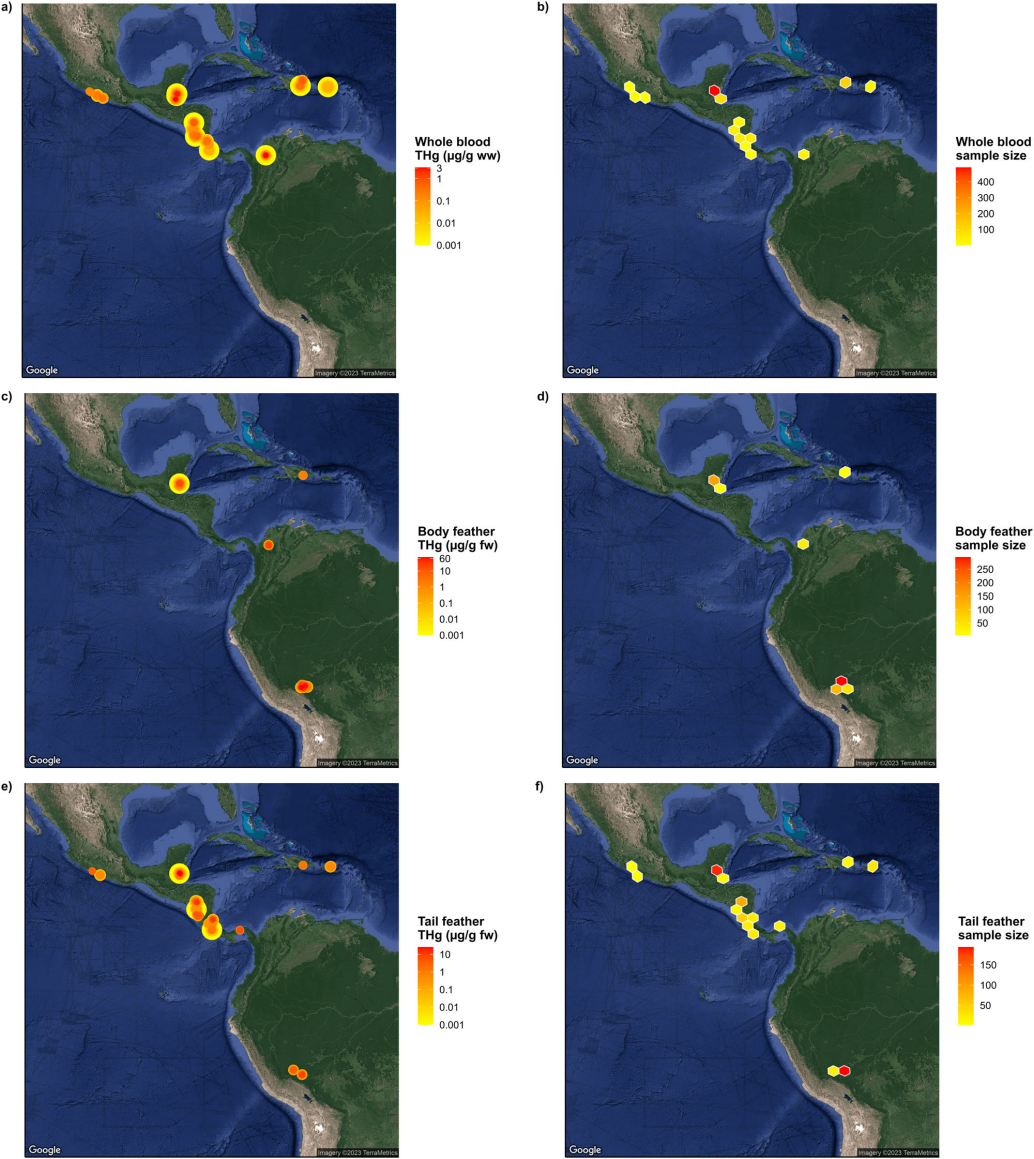
Total mercury (THg) concentrations (µg/g) and sample sizes of birds sampled for (**a**, **b**) whole blood, (**c**, **d**) body feathers, and (**e**, **f**) tail feathers across Central America, South America, and the West Indies from 2007–2023. Point size and color are arranged in order of increasing THg concentration and hexagonal grid cells are colored in terms of increasing sample size

We must also stress, however, that the Neotropics are in great need of widespread Hg sampling beyond these hotspots, and that reducing the spatial coverage of biomonitoring efforts would be counterproductive to articulating Hg exposure and risk on a multi-continental scale. Historical and present sampling efforts have largely been confined to accessible locations with adequate infrastructure, often near tourist destinations within politically-stable countries. As such, we still consider the Neotropical realm to be poorly assessed in terms of avian Hg exposure. We urge researchers to consider venturing outside of our established sampling sites (Fig. 5; Table S2), and highlight that inclusive collaboration provides a viable solution to mitigate current geographic sampling biases (see *The Tropical Research for Avian Conservation & Ecotoxicology (TRACE) Initiative*).

Finally, following the recommendations of Jackson et al. (2015), conservation ornithologists should consider incorporating Hg exposure metrics into mark-recapture and full-annual-cycle population models to isolate the impacts of pollution from a plethora of other stressors, including land-use and climate changes. Such models would be most appropriate for resident and migratory species that have the highest biomagnification potential, the highest probability to interact with ASGM-polluted landscapes, or the highest conservation concern (see *Sentinel species selection*).

### Tissue selection

Neotropical bird THg concentrations differed among tissue types across individuals (*χ*^2^ = 3358.75, *p* < 0.001; Table S5), which highlights the importance of selecting sampling tissues appropriately when organizing Hg biomonitoring efforts. Funding and logistical constraints can often influence tissue selection decisions, since there are different equipment, analysis, or storage requirements. While both internal and external tissues are appropriate for monitoring MeHg exposure in birds (Ackerman et al. 2016a; Fuchsman et al. 2017; Evers 2018), we present economical, nonlethal, and minimally-invasive options suitable for conservation research: blood and feather collection from mature birds. Non-viable eggs are another popular, non-invasive sampling option, and are particularly useful for estimating a laying female’s MeHg exposure during egg development (Ackerman et al. 2016b; Espín et al. 2016). However, in tropical moist forest habitats where nests are difficult to find and access, eggs may not be appropriate for rapid assessments. Tissue selection should ultimately be informed by the desired research endpoint, as different tissues represent different temporal or geographic scales of MeHg exposure. To help guide tissue sampling selections for avian Hg assessments, we provide a procedural and inferential flowchart in Table S9.

Whole blood is perhaps the most favorable and commonly sampled tissue for Hg biomonitoring, and is the most reliable proxy for determining MeHg body burden in birds (Ackerman et al. 2016a, b; Low et al. 2020). Whole blood Hg concentrations are strongly correlated with those in other internal tissues, such as eggs (Ackerman et al. 2016b), as well as in tissues that can only be assessed through euthanasia, such as muscle and liver (Eagles-Smith et al. 2008; Ackerman et al. 2016b). This strong correlation facilitates both the confident conversion of Hg concentrations and the comparison of toxicity reference values among tissue types when necessary (Ackerman et al. 2016a). Whole blood samples represent short-term dietary MeHg exposure from days to weeks (Evers 2018); which, for taxa not in active migration, also reliably represents site-specific MeHg exposure. Therefore, whole blood can be considered a spatiotemporal “snapshot” of MeHg exposure, and is the ideal sampling matrix when seeking to quantify the spatial distribution of MeHg sources across a landscape or region (Evers et al. 2005; Sayers et al. 2021).

Whole blood sampling is complicated by bulky, expensive equipment and challenging storage requirements relative to other nonlethal sampling options, including feathers. Accepted whole blood sampling procedures require the use of coolers, ice packs, and plastic vacutainers to temporarily insulate and protect blood-collection receptacles, especially glass microhematrocirit capillary tubes. These materials can be cumbersome in the field and require careful handling to avoid breakage. Blood-collection receptacles also need to be sealed and stored below freezing with minimal freeze-thaw cycles to prevent changes in Hg concentrations due to contamination, moisture loss, and interconversions of Hg species (Horvat and Byrne 1992; Varian-Ramos et al. 2011; Sommer et al. 2016; Evers et al. 2021). At remote tropical field sites, resources to transport and store samples may be difficult to organize or access, which precludes the use of whole blood as a sampling tissue. However, the collection of dried blood spots on filter paper cards may present a convenient alternative in remote tropical environments since samples do not need to be refrigerated (Chaudhuri et al. 2009; Basu et al. 2017; Perkins and Basu 2018; Nyanza et al. 2019; Santa-Rios et al. 2021)—all while representing the same spatiotemporal scale of MeHg exposure.

Feathers are another preferred tissue for avian Hg monitoring efforts in both temperate (Cristol et al. 2008; Evers et al. 2008; Jackson et al. 2011; Lane et al. 2020) and tropical biomes (Burger and Gochfeld 1991; Anbazhagan et al. 2021; Parolini et al. 2021) due to their unique spatiotemporal Hg signal and convenient sampling, transportation, and storage requirements (Furness et al. 1986; Burger 1993; Bortolotti 2010; Espín et al. 2016). During molting periods, when feathers are connected to the bloodstream, birds can mitigate their body burden by depurating toxicants from internal tissues into growing feathers (Furness et al. 1986; Braune and Gaskin 1987; Burger 1993; Markowski et al. 2013). Following feather maturation, the MeHg bound to feather keratin structures is chemically stable and becomes isolated from the rest of the body (Applequist et al. 1984). The amount of Hg detected in a single feather thus represents a bird’s dietary MeHg exposure and endogenous accumulation throughout the time of feather growth (Burger et al. 1992; Evers et al. 2005; Markowski et al. 2013; but see Furness et al. 1986).

Depending on a species’ molt strategy and movement behavior during molt, feather Hg concentrations can represent a much wider spatiotemporal scale of MeHg exposure than whole blood (Evers 2018). This may complement certain research goals and even allow for ex situ Hg monitoring, but is a strong disadvantage when trying to pinpoint MeHg sources in a landscape. Feather sampling is also complicated by high Hg variation among feathers within or between tracts from the same individual (Bond and Diamond 2008; Carravieri et al. 2014; Ackerman et al. 2019; Peterson et al. 2019; Low et al. 2020) and poor prediction of Hg concentrations in internal tissues, especially for migratory species (Eagles-Smith et al. 2008; Ackerman et al. 2019; Low et al. 2020). As such, blood sampling may be the only approach for gathering a precise spatiotemporal estimate of MeHg exposure for birds that make large seasonal movements (Bildstein 2004; Hobson et al. 2003; Hsiung et al. 2018). In contrast, feather sampling can be more appropriate for species with limited movements, where feather molt occurs near the sampling location (Ackerman et al. 2012). Additional exceptions exist for species with well-documented molt cycles and individuals with accompanying telemetry or isotope data, where we can accurately estimate the geographic origin of Hg detected in a feather (Fort et al. 2014; Ma et al. 2021). Because many Neotropical resident species maintain small territories (generally < 64 ha, Terborgh et al. 1990; Jirinec et al. 2018), feathers from nonmigratory taxa may closely resemble the geographic MeHg signal of blood, while integrating MeHg exposure over a longer time period. Considering these dynamics, researchers should obtain a robust understanding of the molt and movement ecology of their focal species before selecting appropriate sampling tissues.

External contamination is yet another necessary variable to consider when analyzing feathers for Hg. As a general rule, MeHg constitutes approximately 95% of the total Hg detected in biological tissues, including blood (Rimmer et al. 2005), eggs (Ackerman et al. 2013), and feathers (Thompson and Furness 1989). Analyzing tissue samples for total Hg (THg), a much cheaper alternative to MeHg, allows researchers to substantially reduce laboratory costs. However, in unique circumstances, such as museum specimens preserved with mercuric chloride (HgCl_2_) (Goldberg 1996) or feathers sampled near ASGM zones with high inorganic Hg emissions and re-emissions, researchers should analyze feathers for MeHg. This practice avoids the potential bias from exogenous contamination from the adsorption of inorganic Hg on the feather surface (Vo et al. 2011; Markowski et al. 2013; Evers et al. 2014; Dzielski et al. 2019; Perkins et al. 2020). Nevertheless, the convenience of feather sampling outweighs some of the shortcomings.

Logistical advantages of sampling feathers include that they can be plucked without causing permanent damage, sampled repeatedly in the same individual to illustrate temporal bioaccumulation trends, have a much lower risk of disease transmission than blood, are resistant to environmental degradation, and are accessible via natural history collections (Evers et al. 2008; Bortolotti 2010; Espín et al. 2016; Dzielski et al. 2019; Perkins et al. 2020). As with dried blood spots, the chemical stability of MeHg within keratin-based tissues precludes the need to store and transport feather samples below freezing (Applequist et al. 1984; Espín et al. 2016; Evers et al. 2021), which profoundly simplifies field planning in remote tropical locations. In addition, feather sampling circumvents the logistical complications surrounding the transmission of Exotic Newcastle Disease and the H5N1 subtype of Highly Pathogenic Avian Influenza when importing and exporting samples internationally (Paul 2005), since MeHg in fully-grown feathers remains stable when exposed to extreme treatments (Applequist et al. 1984; Goede and de Bruin 1984).

Once familiarizing themselves with the life history of their focal species, researchers interested in sampling feathers for Hg analysis should consider tail and flank feathers as useful standards. The sampling of two symmetrical outer tail feathers (rectrices, typically R6) is less invasive compared to flight feathers of the wing (remiges)—which have greater implications for flight efficiency, predator avoidance, and migratory success (Swaddle et al. 1996; Lind et al. 2010; McDonald and Griffith 2011)—allows for consistent comparisons among individuals (Varela et al. 2016), and also allows for the measurement of Hg-related fluctuating asymmetry (Espín et al. 2016). Additionally, many species have well-documented and predictable tail feather molt cycles, which reduces the temporal uncertainty of Hg exposure (Bortolotti 2010). Flank feathers can be easily plucked and analyzed whole, are typically larger and provide more mass than adjacent breast or belly feathers—which is useful for the minimum mass requirement in direct Hg analysis—and tend to have lower Hg variation relative to other feather tracts (Furness et al. 1986; Low et al. 2020). Since museum curators aim to minimize destructive sampling, flank feathers offer a unique opportunity to conduct retrospective analyses using preserved specimens in natural history collections (Applequist et al. 1984; Vo et al. 2011; Evers et al. 2014; Dzielski et al. 2019; Perkins et al. 2020). In Neotropical migratory songbirds, while flight feathers may only be molted once a year during the postbreeding phase prior to autumn migration, body feathers can be molted twice a year, and represent two distinct spatiotemporal periods of MeHg exposure (Pyle 1997; Rohwer et al. 2005). These combined traits make flank feathers one of the most favorable sampling matrices for avian Hg monitoring (Furness et al. 1986). Despite the inherent complications and advanced considerations necessary when sampling feathers, their ease of collection and storage, especially in regions with limited-resources, and the chemical stability of MeHg within keratin-based complexes, make feathers a desirable tissue choice for some Hg monitoring situations.

### Sentinel species selection

Sentinel species provide a convenient and efficient proxy for quantifying contaminant risk at various temporal and spatial scales. Distilling the writing of Beeby (2001) and Jackson et al. (2015), in general, sentinel species should be abundant and widespread to facilitate extensive sampling and geographic comparisons, live long enough and occupy a home range that matches the spatiotemporal scale in question, and have a relatively well-documented life history that can provide context for how contaminants affect their behavior, physiology, and reproduction. More simply, an effective sentinel species is one that best complements the research question or system of interest. With over 3500 bird species native to the Neotropics, the selection of sentinel species is much more challenging than for studies based in temperate biomes. Due to the high diversity and low density of Neotropical species, broader taxonomic or functional groups may even be more useful sentinels than individual species (Lacher and Goldstein 1997). Because our cumulative sampling efforts are not exhaustive, and the vast majority of Neotropical species remain unsampled (i.e., this synthesis provides THg exposure profiles for only about 9% of Neotropical species), we cannot produce a definitive list of all relevant and thus, irrelevant species. Here, we present a conservative list of species that have good potential to act as sentinels for various Neotropical habitats and regions given our current understanding (Table S10).

Piscivorous birds have particular advantages as sentinel species. Both small and large piscivores, including species in Alcedinidae (kingfishers) and Ardeidae (herons), respectively, are at a high risk of Hg exposure due to their high trophic position and association with aquatic environments. Alcedinidae and Ardeidae feather THg concentrations have exceeded 6 µg/g fw in previous studies (Herring et al. 2009; Zamani-Ahmadmahmoodi et al. 2009), and ascend to 72 µg/g fw in this study (Table S8)—highlighting their bioaccumulation potential. Due to the global distribution of these families, ecotoxicologists have the opportunity to compare Hg concentrations in Neotropical species of Alcedinidae and Ardeidae to those observed in species elsewhere around the world. Concurrent logistical advantages include that species in Alcedinidae can be captured using passive ground-level mist-net arrays and, due to their aggressive territoriality, can also be lured with auditory playback tools. Additionally, species in Ardeidae nest in large colonies—meaning that samples can be collected more efficiently once a nesting site is identified. Due to their relative trophic position, habitat occupancy, logistical advantages, and previous Hg research focused on these families, members of Alcedinidae and Ardeidae, such as *Chloroceryle amazona* (Amazon kingfisher), *Chloroceryle americana* (green kingfisher), *Chloroceryle aenea* (American pygmy-kingfisher), and *Ardea alba* (great egret), are well-suited as sentinel piscivores for Neotropical aquatic habitats.

Due to their high bioaccumulation potential and convenience of sampling, invertivorous songbirds are ideal avian sentinels to assess Hg contamination across terrestrial habitats (Jackson et al. 2015; Cristol and Evers 2020)—especially in unison with established mist-netting surveys. Two avian invertivore clades of particular interest to Neotropical regions are ant-following birds and Neotropical migrants, both of which exhibit elevated THg concentrations relative to other invertivores (Figs. 3, S4; Table S7).

An estimated 465 Neotropical bird species engage in obligate, facultative, or opportunistic ant-following behavior—one of the most conspicuous and captivating foraging spectacles native to wet forest biomes of Central and South America—by targeting invertebrate or vertebrate prey items that attempt to escape foraging army ant swarms (Oniki and Willis 1972; Stotz et al. 1996; Roberts et al. 2000; Wrege et al. 2005; Tórrez et al. 2009). New World army ants, particularly *Eciton burchellii* and *Labidus praedator*, are obligate nomadic group predators that roam the forest floor in large raids while searching for their preferred prey items: mainly pupae of other ant species (Willis and Oniki 1978; Powell 2011). Army ants will also consume virtually any organism that they can immobilize, including frogs, lizards, snakes, spiders, and nestling birds (Breed and Moore 2016). Several bird families that regularly attend swarms, such as Thamnophilidae (antbirds), Furnariidae (ovenbirds), Momotidae (motmots), Tyrannidae (tyrant flycatchers), Cuculidae (cuckoos), Troglodytidae (wrens), and Parulidae (New World warblers), are among the highest-ranking families in predicted and mean THg concentrations (Figs. 3, S4). While ant-following species are not thought to target the ants themselves when foraging, individuals may consume ants infrequently as bycatch (Willis and Oniki 1978). Thus, ant-followers may experience elevated Hg exposure due to both increased access to higher trophic level prey items, such as spiders and lizards, or the accidental consumption of predatory ants. Given their invertivorous trophic niche and widespread distribution throughout Latin America, within the ant-following clade, several species that standout as potential sentinels include *Formicarius analis* (black-faced antthrush), *Taraba major* (great antshrike), *Sittasomus griseicapillus* (olivaceous woodcreeper), and *Eucometis penicillata* (gray-headed tanager). These species all present ideal opportunities for continental-scale comparative analyses throughout the Neotropical realm.

Ant-following birds present a model system with unique conservation significance for Neotropical wet forests (Martínez et al. 2021). Further, ant-following food web dynamics have yet to be described by the ecotoxicological community. Given the placement of army ants as keystone species (Martínez et al. 2021), the presence and impacts of Hg in the ant-following system should be further scrutinized. In sampling ant-following taxa, which are among the most frequently-captured individuals in passive ground-level mist-net surveys, we can (1) better understand how Hg migrates and biomagnifies through Neotropical terrestrial systems, (2) quantify the toxicological impacts of ASGM on declining biota, and (3) articulate the presence and influence of Hg throughout the full annual-cycle for resident and migratory species.

Birds of prey are perhaps the most charismatic taxonomic group worth considering as avian Hg sentinels. Because raptors occupy apex trophic positions of tropical terrestrial food webs, they provide a unique biomagnification profile relative to avian piscivores and invertivores. Raptors often bioaccumulate elevated Hg concentrations in both temperate and tropical biomes (DeSorbo et al. 2018; Bourbour et al. 2016; Keyel et al. 2020), and have repeatedly served as focal organisms for Hg assessments in Latin America (Shrum 2009; Elliott et al. 2015; Canham et al. 2020; Balza et al. 2021). Following the relationships illustrated for temperate raptors (Bourbour et al. 2016; Keyel et al. 2020), larger Neotropical raptors in Accipitridae (hawks, eagles, and kites) that hunt primarily granivorous or frugivorous prey groups such as rodents, primates, and sloths, would not be expected to bioaccumulate elevated Hg concentrations due to the low trophic positions of their preferred prey items. However, members of Falconidae (falcons and caracaras) that scavenge marine carrion (Balza et al. 2021), or hunt birds, bats, lizards, or insects are more likely to experience increased biomagnification. For example, the opportunistic ant-following, and primarily bird- and insect-eating, *Micrastur ruficollis* and *Micrastur gilvicollis* (barred and lined forest-falcons; Oniki and Willis 1972) exhibited the highest feather THg concentrations among raptor species sampled in Madre de Dios, Peru (Shrum 2009; Figs. 4, S5). While raptors are generally sparsely-populated, difficult to capture, and maintain much larger home ranges than songbirds, baited traps can be an effective way of assessing apex biomagnification and intra-taxonomic variation within this clade across large spatial scales of interest (Bloom et al. 2007; Shrum 2009).

Deriving Hg effect concentrations in wild bird populations is most feasible for species that can be efficiently accessed and sampled relative to more cryptic nesters. These can include cavity-nesting species that utilize man-made nest boxes (Brasso and Cristol 2008; Jackson et al. 2011), colonial-nesting species (King et al. 1991; Henny et al. 2002; Hill et al. 2008), or species that are easily observed and tracked over time (Evers et al. 2008). Therefore, obligate or opportunistic cavity-nesting piscivores and invertivores, including members of Alcedinidae (kingfishers), Momotidae (motmots), Hirundinidae (swallows), Furnariidae (ovenbirds), and Troglodytidae (wrens)—as well as colonial-nesting piscivores, including members of Ardeidae (herons)—could make ideal candidate species for monitoring Hg impacts to avian health in the Neotropics.

### The Tropical Research for Avian Conservation & Ecotoxicology (TRACE) Initiative

Mercury biomonitoring efforts over the past century have pursued divergent sampling strategies and focal species (including this study), while neglecting the most biologically-rich regions on the planet. These decisions have limited our ability to make meaningful comparisons across respective datasets and biological realms. Therefore, the field of ecotoxicology stands to benefit greatly from a coordinated effort that aims to resolve current uncertainties by overcoming previous methodological inconsistencies. We offer potential solutions in the form of (1) a consolidated bird sampling protocol for Hg analysis (Evers et al. 2021), and (2) an international, collaborative data-sharing platform known as the Tropical Research for Avian Conservation and Ecotoxicology (TRACE) Initiative (https://www.briwildlife.org/TRACE). TRACE builds upon the international collaborations responsible for generating the data we showcase in this manuscript, and offers a framework for a variety of researchers and stakeholders to interact (e.g., local, state, and federal governmental agencies, nongovernmental conservation organizations, academic institutions, natural history museums, trace element laboratories, and local communities). Our bird Hg sampling protocol provides a meaningful complement to this platform and a set of instructions to standardize focal species and sampling tissue selection, as well as data management practices, among adjacent ecotoxicological research efforts (Evers et al. 2021). By synchronizing these efforts, we can effectively maximize the comparability of data across time and space, and minimize the redundant use of resources to achieve intended research goals.

Notably, TRACE also provides an opportunity to dismantle systemic barriers and colonialist precedents within the field of Neotropical ornithology (van Deelen 2022; Ruelas Inzunza et al. 2023; Soares et al. 2023). Fundamental to the TRACE Initiative is to distance ourselves from “parachute science,” where researchers from the Global North exploit local knowledge in the Global South to collect data, and return to Western societies to disseminate their findings via inaccessible communication outlets (e.g., for-profit journals)—all without involving local stakeholders in the scientific process (Asase et al. 2022). Therefore, through an equitable exchange of expertise, funding, resources, data, and authorship among collaborators, TRACE can: (1) generate crucial research capacity and leadership opportunities in tropical nations, which are disproportionately impacted by environmental crises, and (2) produce standardized information on the prevalence, distribution, toxicokinetics, and biogeochemistry of Hg—using birds as a convenient, ecologically-important, and charismatic proxy. Ultimately, we aim to broadly disseminate this standardized information to empower local communities and inform national and international policies, including those coordinated by Parties of the United Nations Minamata Convention on Mercury.

## Conclusion

This synthesis provides unprecedented baseline knowledge of Neotropical bird Hg exposure and is a valuable first step in improving our understanding of how Hg could potentially impact avian health and productivity throughout Central America, South America, and the West Indies. Given the limited spatial density and extent of these data, we cannot expect our findings to be representative of the entire Neotropical realm. However, we highlight that Hg exposure to avian communities extends well beyond pollution hotspots and illustrate the general ubiquity of this heavy metal across Neotropical regions. Due to the previously mounting uncertainty surrounding avian Hg exposure in the Neotropics, recent, and otherwise comprehensive, publications on Neotropical bird declines have not acknowledged Hg as a potential stressor (Robinson 1999, 2001; Sigel et al. 2006; Latta et al. 2011; Blake and Loiselle 2015; Boyle and Sigel 2015; Powell et al. 2015; Stouffer et al. 2020; Sherry 2021; Pollock et al. 2022). We believe that our findings merit the consideration of Hg emission, deposition, methylation, and biomagnification when setting priorities for tropical avian conservation and management, and more broadly, defining global biodiversity targets (Mueller et al. 2022; Sigmund et al. 2022).

With the exception of Barbados, Belize, French Guiana, Guatemala, Grenada, Haiti, Saint Vincent and the Grenadines, and Trinidad and Tobago, all Neotropical nations have acceded or ratified both the UN Convention on Biological Diversity and the Minamata Convention on Mercury (CBD Secretariat 2021; MCM 2021). As such, there is overwhelming support to understand how Hg impacts public health, ecosystem function, and biodiversity conservation. While international agreements provide the framework and capacity to implement solutions, birds—as indicators of ecosystem health—offer the ideal sampling foci to generate crucial information on this accelerating issue across the Pantropical realm.

### Appendix 1. Supplementary materials

Supplementary materials include figures displaying mean THg concentrations among avian trophic niches, primary habitat associations, orders, families, species, and sampling sites (Figs. S1–6); predicted THg concentrations among sites (Fig. S7); and predicted seasonal THg concentrations among trophic niches (Fig. S8); tables displaying publications that report Hg exposure to birds in the Neotropics (Table S1); mean THg concentrations among countries, sites, trophic niches, primary habitat associations, and taxonomy (Tables S2, S6–8); taxonomic, foraging guild, primary habitat, and migratory classifications assigned to sampled bird species (Table S3); model selection and analysis of variance results (Tables S4–5); an inferential flowchart for tissue selection (Table S9); and potential avian sentinel species (Table S10).

### Supplementary information

SupplementaryMaterials_NeotropicalBirdHgSynthesis

## Data Availability

All data and software curated for this research are archived and available at https://github.com/csayers2/Neotropical-Bird-Hg-Synthesis.
