# Supplementary material for: Mercury in Neotropical birds: a synthesis and prospectus on 13 years of exposure data
Source: Ecotoxicology. 2023 Oct 31;32(8):1096–123. doi: 10.1007/s10646-023-02706-y (PMC10622370; doi:10.1007/s10646-023-02706-y)
Supplement: Supplementary file 1 — SupplementaryMaterials_NeotropicalBirdHgSynthesis [file 10646_2023_2706_MOESM1_ESM.pdf]

Mercury in Neotropical birds: a synthesis and prospectus on 13 years of exposure data
Exposición al mercurio en aves Neotropicales: una síntesis y prospecto sobre 13 años de datos

Christopher J. Sayers II[1,2,3*] (csayers2@ucla.edu; 0000-0002-7394-3929), David C. Evers[2] (david.evers@briwildlife.org), Viviana Ruiz-Gutierrez[4] (vr45@cornell.edu; 0000-0001-7116-1168), Evan Adams[2] (evan.adams@briwildlife.org; 0000-0002-4327-6926), Claudia M. Vega[3,5] (vegacm@wfu.edu; 0000-0003-4012-2363), Jessica Pisconte[3] (pisconj@wfu.edu), Vania Tejeda[3] (vaniat_36@hotmail.com), Kevin Regan[2] (kevin.regan@briwildlife.org), Oksana P. Lane[2] (oksanalane@yahoo.com), Abidas A. Ash[6] (aash@ub.edu.bz), Reynold Cal[7] (raycalsm@gmail.com), Stevan Reneau[7] (stevan_reneau@yahoo.com), Wilber Martínez[7] (wadmartinez@yahoo.com), Gilroy Welch[7] (gilroywelch30@gmail.com), Kayla Hartwell[7] (kaylahartwell@gmail.com; 0000-0003-4062-7193), Mario Teul[7] (teulmario2@gmail.com), David Tzul[7] (cracid2000@yahoo.com), Wayne J. Arendt[8] (waynearendt@gmail.com; 0000-0001-8462-4416), Marvin A. Tórrez[9] (mtorrez@uca.edu.ni), Mrinalini Watsa[10,11] (merkenswickwatsa@sdzwa.org; 0000-0002-8130-8810), Gideon Erkenswick[11] (gideon@fieldprojects.org; 0000-0001-6040-1170), Caroline E. Moore[10] (camoore@sdzwa.org; 0000-0001-6999-1656), Jacqueline Gerson[12] (gersonja@msu.edu; 0000-0001-5228-447X), Victor Sánchez[13] (victor.sanzca@gmail.com; 0000-0002-3316-973X), Raúl Pérez Purizaca[14] (r.perezpurizaca@gmail.com), Helen Yurek[2] (helen.yurek@briwildlife.org), Mark E.H. Burton[2] (mark.burton@briwildlife.org; 0000-0002-1566-8869), Peggy L. Shrum[15] (plsbirds817@yahoo.com; 0000-0002-4426-7271), Sebastian Tabares-Segovia[16] (setabaress@unal.edu.co; 0009-0003-4486-5743); Korik Vargas[2] (korik.vargas@briwildlife.org); Finola F. Fogarty[17,18] (fogartyfinola@gmail.com; 0000-0002-1729-539X), Mathieu R. Charette[18] (mcharette@treesociety.org; 0000-0002-8236-2800), Ari E. Martínez[19] (arimartinez043@gmail.com), Emily S. Bernhardt[20] (emily.bernhardt@duke.edu), Robert J. Taylor[21] (rtaylor@cvm.tamu.edu), Timothy H. Tear[2] (timothy.tear@briwildlife.org), Luis E. Fernandez[3,5,22] (fernanle@wfu.edu; 0000-0003-3879-1445)

[1]Department of Ecology and Evolutionary Biology, University of California, Los Angeles, CA 90095, USA
[2]Center for Mercury Studies, Biodiversity Research Institute, 276 Canco Road, Portland, ME 04103, USA
[3]Centro de Innovación Científica Amazónica, Puerto Maldonado, Madre de Dios 17000, Peru
[4]Cornell Lab of Ornithology, 159 Sapsucker Woods Road, Ithaca, NY 14850, USA
[5]Department of Biology, Center for Energy, Environment and Sustainability, Wake Forest University, Winston-Salem, NC 27106, USA
[6]University of Belize Environmental Research Institute, Price Center Road, P.O. Box 340, Belmopan, Cayo District, Belize
[7]Foundation for Wildlife Conservation, Tropical Education Center, 28 George Price Highway, P.O. Box 368, La Democracia, Belize District, Belize
[8]International Institute of Tropical Forestry, USDA Forest Service, 1201 Calle Ceiba, Jardín Botánico Sur, San Juan 00926-1119, Puerto Rico
[9]Instituto Interdisciplinario de Ciencias Naturales, Universidad Centroamericana, Managua, Nicaragua

[10]Beckman Center for Conservation Research, San Diego Zoo Wildlife Alliance, P.O. Box 120551, San Diego, CA 92112, USA
[11]Field Projects International, Escondido CA 92029, USA
[12]Department of Earth & Environmental Sciences, Michigan State University, East Lansing, MI 48824, USA
[13]Instituto de Investigación en Ecología y Conservación, Peru
[14]Universidad Nacional de Piura, Urb. Miraflores S/N, Castilla 20002, Piura, Peru
[15]Department of Fisheries and Wildlife Biology, Clemson University, Clemson, SC 29634, USA
[16]Departamento de Biología, Universidad Nacional de Colombia, Bogotá, 111321, Colombia
[17]Department of Zoology, Faculty of Science, University of British Columbia, Vancouver, BC, Canada
[18]Toucan Ridge Ecology and Education Society, 27.5 Miles Hummingbird Hwy, Stann Creek, Belize
[19]Department of Ecology & Evolutionary Biology, University of California, Santa Cruz, CA 95064, USA
[20]Department of Biology, Duke University, Durham, NC 27708, USA
[21]Department of Veterinary Medicine & Biomedical Sciences, Texas A&M University, College Station, TX 77843, USA
[22]Department of Global Ecology, Carnegie Institution for Science, Stanford, CA 94305, USA
*Corresponding author: csayers2@ucla.edu

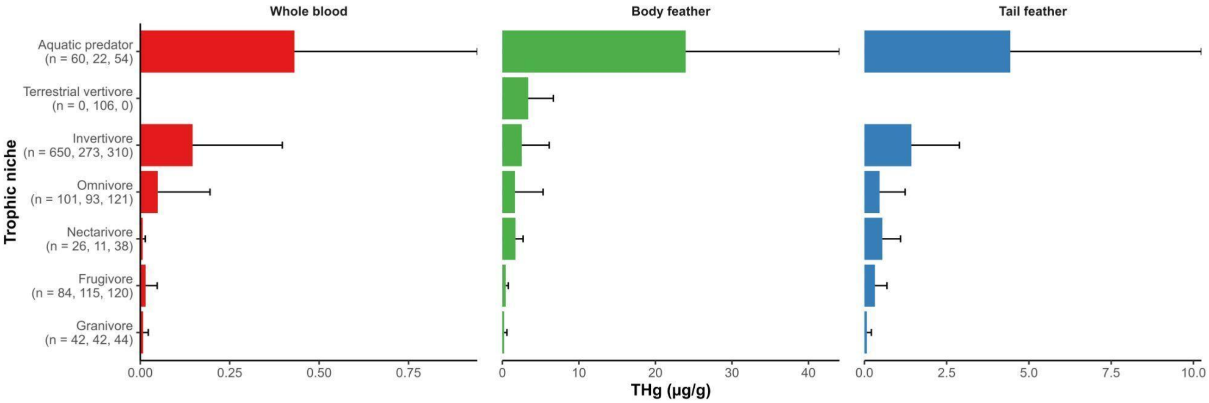

Figure S1. Arithmetic mean ± standard deviation total mercury (THg) concentrations (µg/g) among Neotropical bird trophic niches sampled across Central America, South America, and the West Indies from 2007–2023. Trophic niches are arranged by the maximum percentile rank of mean THg concentrations among tissue types.

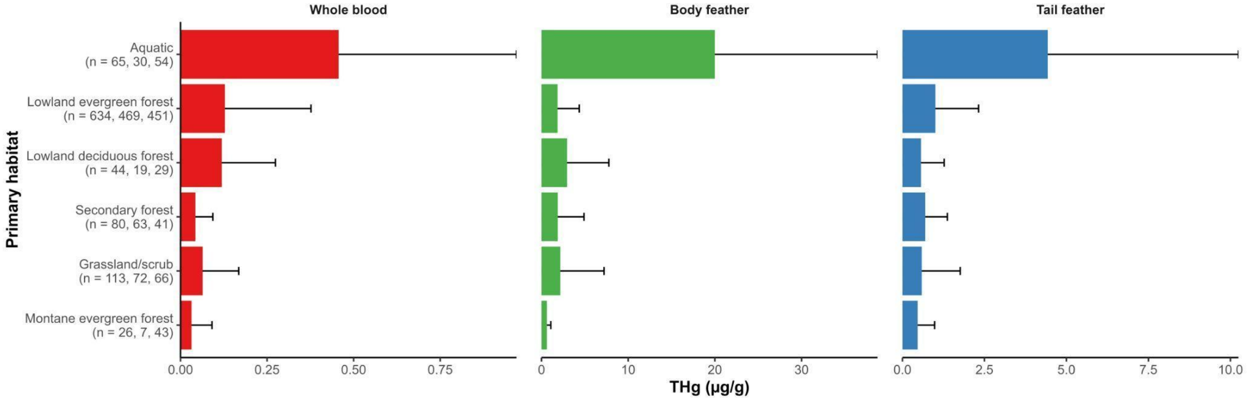

Figure S2. Arithmetic mean ± standard deviation total mercury (THg) concentrations (μg/g) among Neotropical bird primary habitat associations sampled across Central America, South America, and the West Indies from 2007–2023. Habitats are arranged by the maximum percentile rank of mean THg concentrations among tissue types.

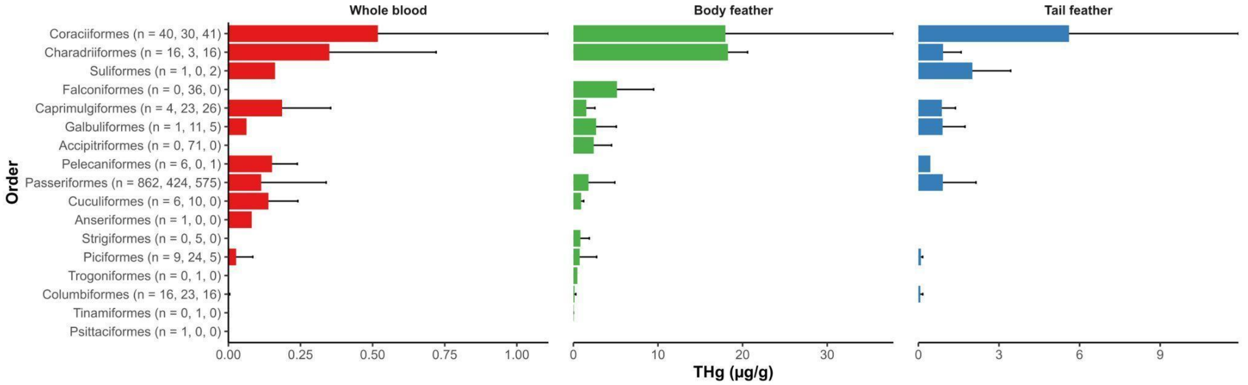

Figure S3. Arithmetic mean ± standard deviation total mercury (THg) concentrations (μg/g) among Neotropical bird orders sampled across Central America, South America, and the West Indies from 2007–2023. Orders are arranged by the maximum percentile rank of mean THg concentrations among tissue types.

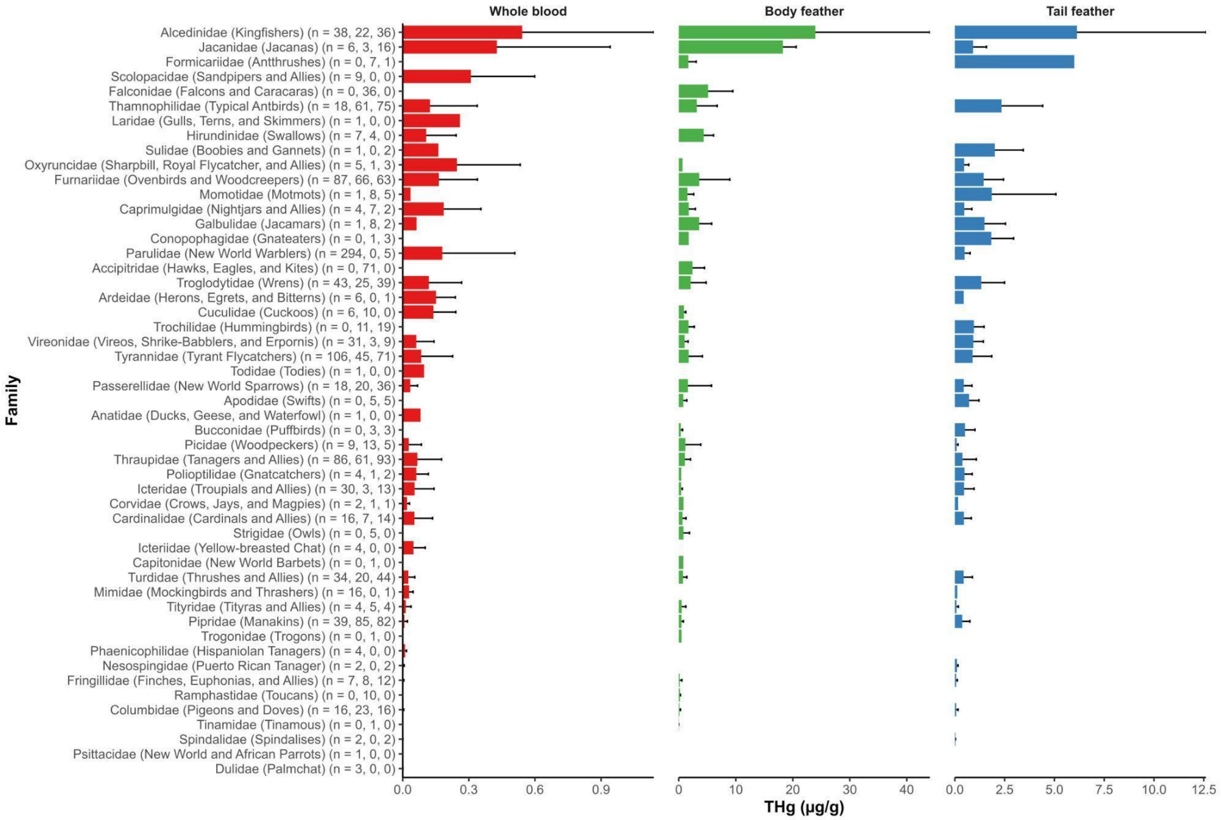

Figure S4. Arithmetic mean ± standard deviation total mercury (THg) concentrations (µg/g) among Neotropical bird families sampled across Central America, South America, and the West Indies from 2007–2023. Families are arranged by the maximum percentile rank of mean THg concentrations among tissue types.

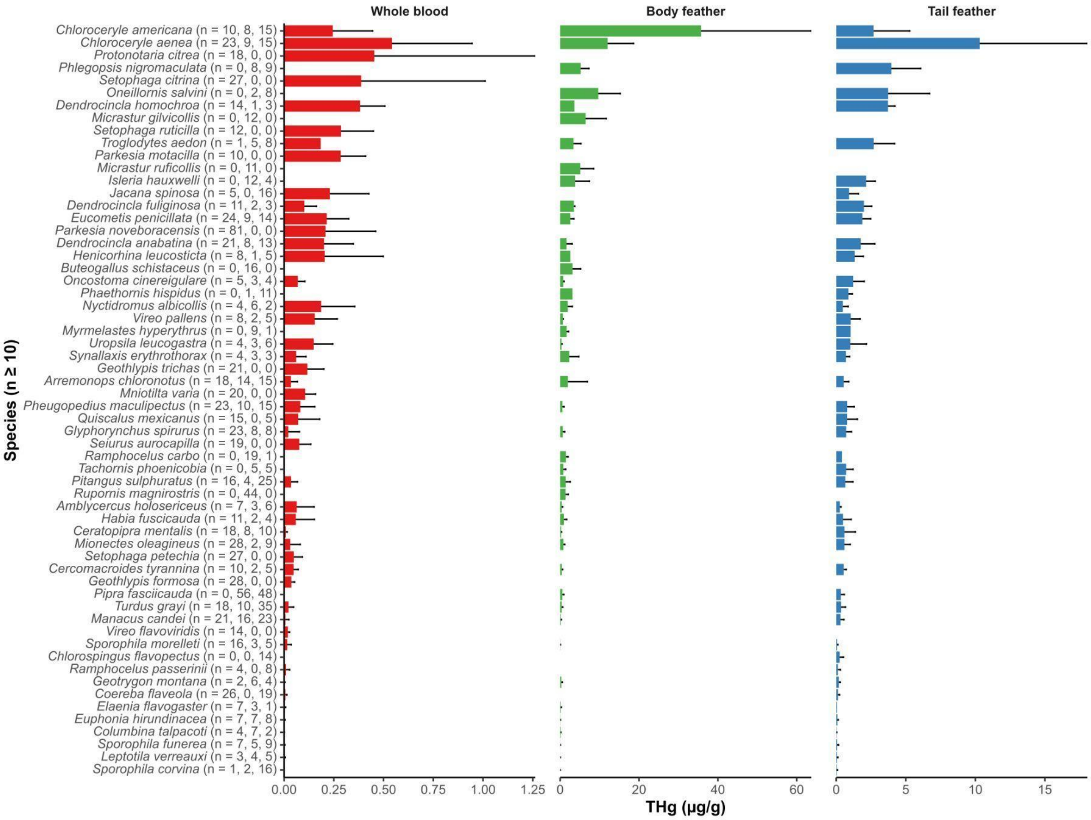

Figure S5. Arithmetic mean ± standard deviation total mercury (THg) concentrations (μg/g) among Neotropical bird species sampled across Central America, South America, and the West Indies from 2007–2023. Species are arranged by the maximum percentile rank of mean THg concentrations among tissue types. Species with fewer than 10 samples are excluded.

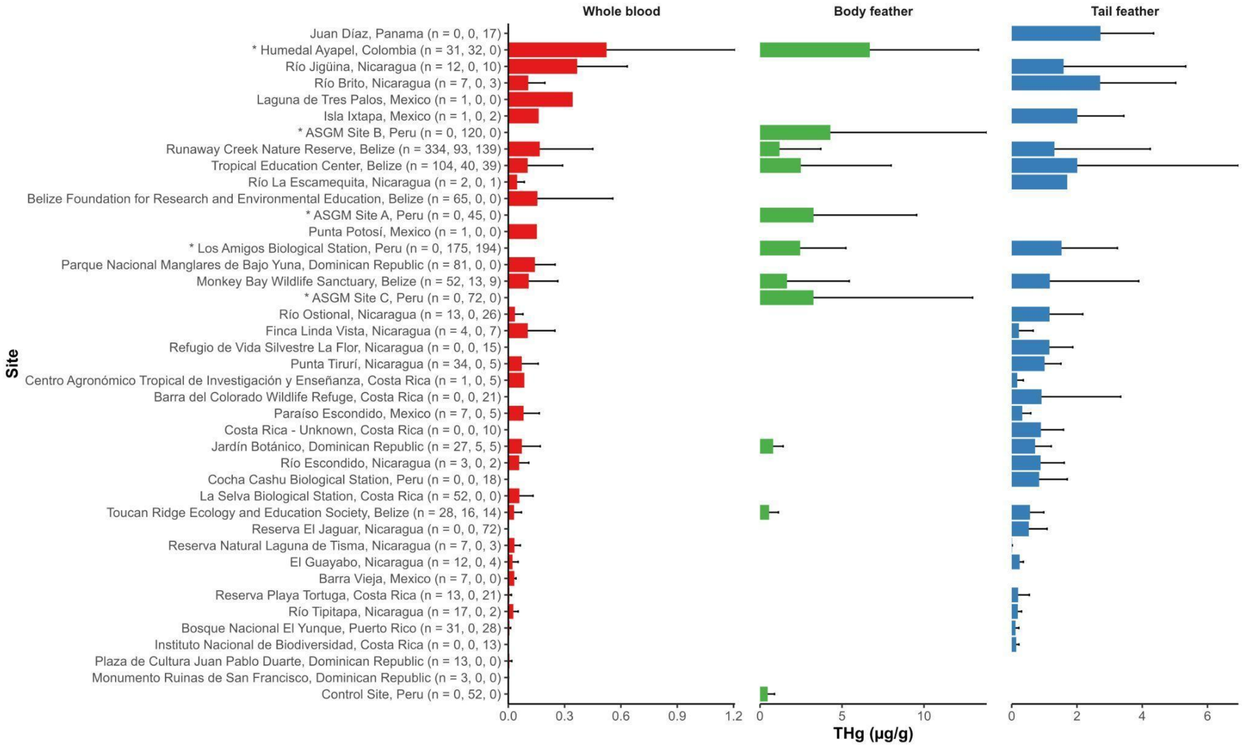

Figure S6. Arithmetic mean ± standard deviation total mercury (THg) concentrations (µg/g) among sites sampled across Central America, South America, and the West Indies from 2007–2023. Sites are arranged by the maximum percentile rank of mean THg concentrations among tissue types. An asterisks (*) indicates that there is artisanal gold mining present within a 7 km radius of the site.

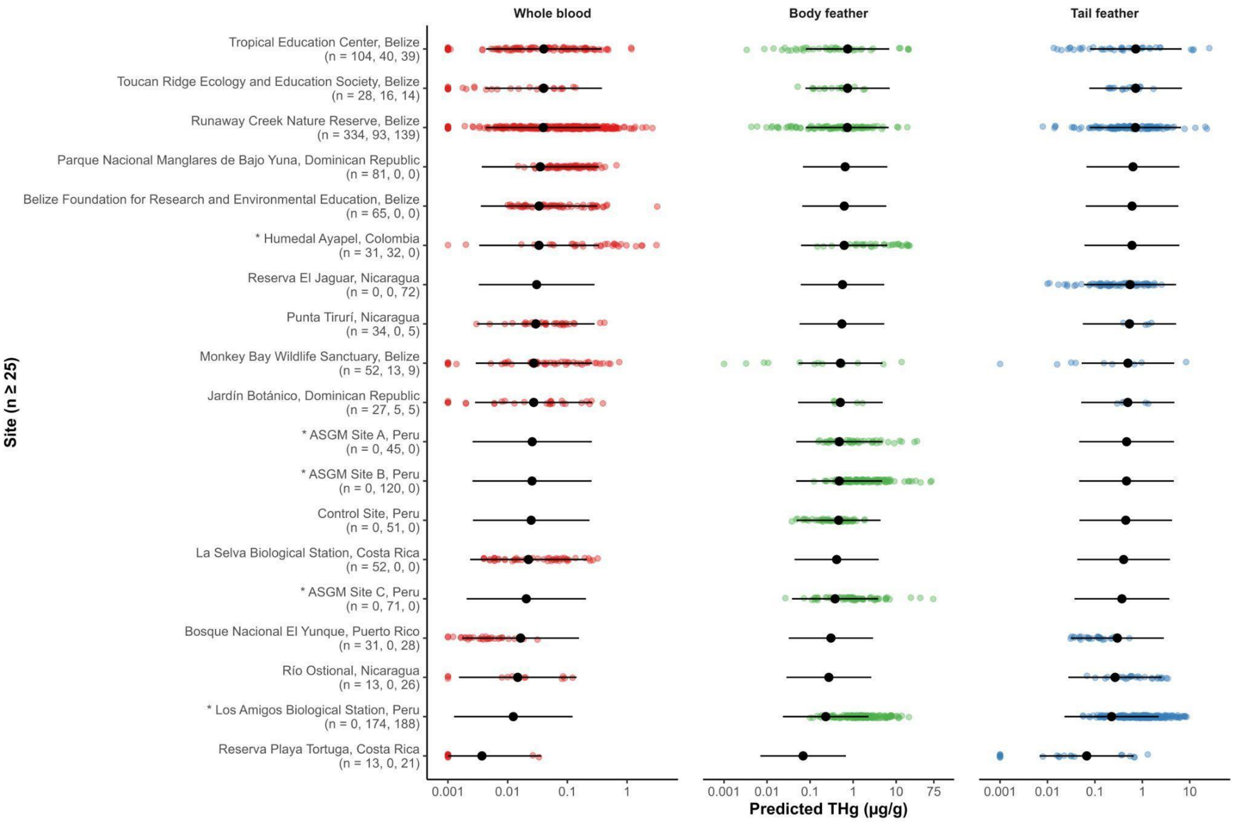

Figure S7. Total mercury (THg) concentrations (µg/g) overlaid with back-transformed predicted means ± 95% confidence intervals among sites sampled across Central America, South America, and the West Indies from 2007–2023. Sites with fewer than 25 samples are excluded. An asterisks (*) indicates that there is artisanal gold mining present within a 7 km radius of the site.

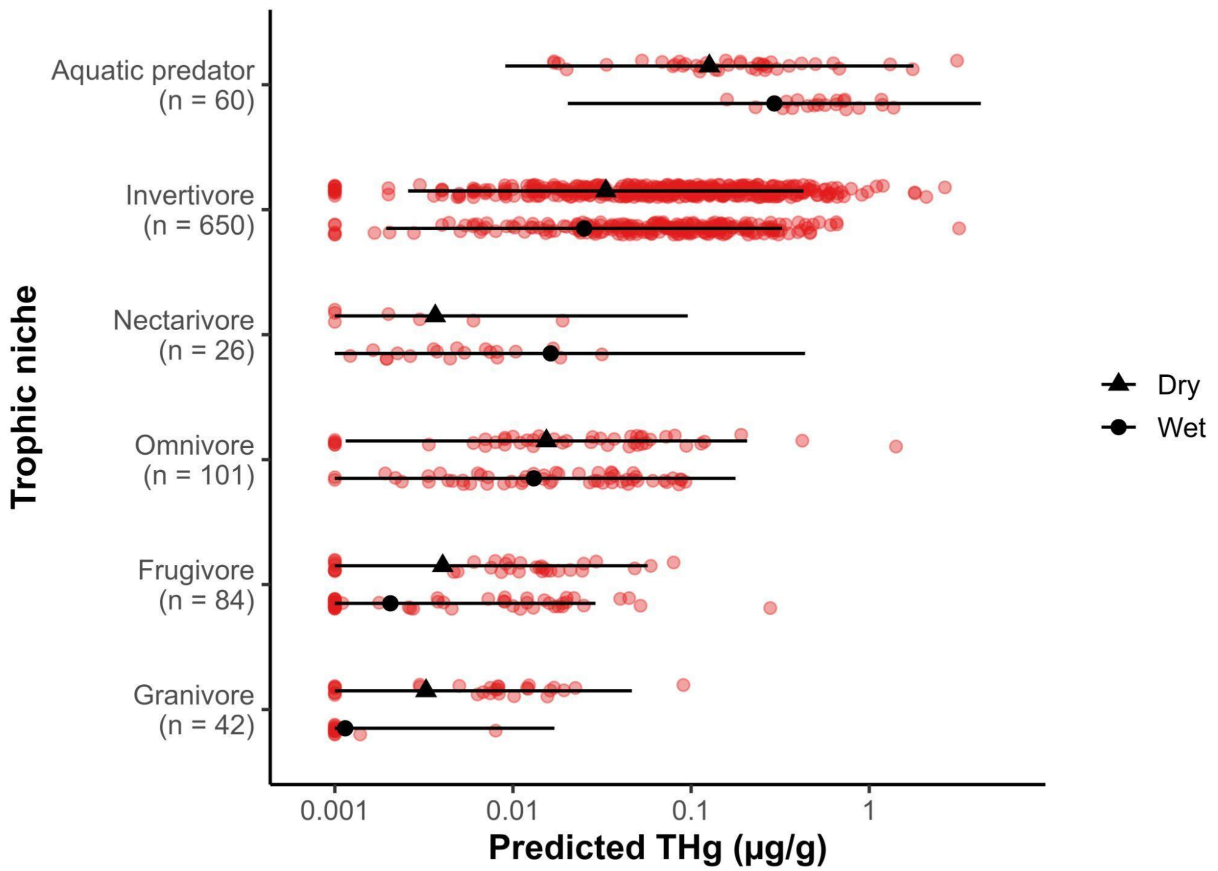

Figure S8. Whole blood total mercury (THg) concentrations (µg/g) overlaid with back-transformed predicted means ± 95% confidence intervals among Neotropical bird trophic niches and seasons sampled across Central America, South America, and the West Indies from 2007–2023. Bird THg concentrations differed between seasons (p = 0.001), and seasonal relationships varied by trophic niche ($p < 0.001$).

Table S1. Publications reporting Hg concentrations in bird taxa sampled in the Neotropics.

Study cited	Title	Countries sampled	Families sampled	Species sampled
Burger and Gochfield (1991)	Lead, mercury, and cadmium in feathers of tropical terns in Puerto Rico and Australia	Puerto Rico	1	4
Burger et al. (1992)	Heavy metal and selenium levels in young cattle egrets from nesting colonies in the northeastern United States, Puerto Rico, and Egypt	Puerto Rico	1	1
Hylander et al. (1994)	Mercury levels in Alto Pantanal: A screening study	Brazil	2	2
Alho and Vieira (1997)	Fish and wildlife resources in the Pantanal wetlands of Brazil and potential disturbances from the release of environmental contaminants	Brazil	5	5
Burger (1997)	Ecological effects and biomonitoring for mercury in tropical ecosystems	Costa Rica, Puerto Rico	3	3
Klekowski et al. (1999)	An association of mangrove mutation, scarlet ibis, and mercury contamination in Trinidad, West Indies	Trinidad and Tobago	2	2
Rimmer et al. (2005)	Mercury concentrations in Bicknell's thrush and other insectivorous passerines in montane forests of northeastern North America	Cuba, Dominican Republic, Haiti	1	1
Evers (2008)	Mercury in terrestrial birds of Belize	Belize	15	36
Shrum (2009)	Analysis of mercury and lead in birds of prey from gold mining areas of the Peruvian Amazon	Peru	1	14
Del Lama et al. (2011)	Sedentary nestlings of wood stork as monitors of mercury contamination in the gold mining region of the Brazilian Pantanal	Brazil	1	1
Albuja et al. (2012)	Niveles de mercurio en aves silvestres de tres regiones mineras del sur del Ecuador	Ecuador	14	22
Lane et al. (2013)	Pilot assessment of mercury exposure in selected biota from the lowlands of Nicaragua	Nicaragua	13	25
Olivero-Verbel et al. (2013)	Morphometric parameters and total mercury in eggs of snowy egret (*Egretta thula*) from Cartagena Bay and Totumo Marsh, north of Colombia	Colombia	1	1
Townsend et al. (2013)	Mercury concentrations in tropical resident and migrant songbirds on Hispaniola	Dominican Republic, Haiti	4	9
Guédron et al. (2017)	Mercury contamination level and speciation inventory in Lakes Titicaca & Uru-Uru (Bolivia): Current status and future trends	Bolivia	2	2
Burger et al. (2018)	Mercury, lead, cadmium, cobalt, arsenic and selenium in the blood of semipalmated sandpipers (Calidris pusilla) from Suriname, South America: Age-related differences in wintering site and comparisons with a stopover site in New Jersey, USA	Suriname	1	1
Sierra-Marquez et al. (2018)	Mercury levels in birds and small rodents from Las Orquideas National Natural Park, Colombia	Colombia	13	37

Evers and Burton (2020)	Mercury monitoring—Belize	Belize	4	6
Shanley et al. (2020)	Resolving a paradox—high mercury deposition, but low bioaccumulation in northeastern Puerto Rico	Puerto Rico	7	8
Dias dos Santos et al. (2021)	Mercury in birds (aquatic and scavenger) from the Western Amazon	Brazil	4	7
Buelvas-Soto et al. (2022)	Bioacumulación de mercurio y plomo en el pato *Dendrocygna autumnalis* en la subregión de la Mojana, Colombia	Colombia	1	1
Gerson et al. (2022)	Amazon forests capture high levels of atmospheric mercury pollution from artisanal gold mining	Peru	2	3
Mancuso et al. (2022)	Mercury toxicity risk and corticosterone levels across the breeding range of the Yellow-breasted Chat	Mexico	1	1
Hurtado et al. (2023)	Mercury and methylmercury concentration in the feathers of two species of Kingfishers *Megaceryle torquata* and *Chloroceryle amazona* in the Upper Paraguay Basin and Amazon Basin	Brazil	1	2
Oliveira et al. (2023)	Green Kingfishers as sentinel species for mercury contamination in Amazon	Brazil	1	1
		**Unique taxonomy across all publications:**	**44**	**171**

Families: Accipitridae, Alcedinidae, Anatidae, Anhingidae, Aramidae, Ardeidae, Calyptophilidae, Cardinalidae, Cathartidae, Ciconiidae, Columbidae, Cotingidae, Cuculidae, Falconidae, Fringillidae, Furnariidae, Grallariidae, Hirundinidae, Icteridae, Icteriidae, Jacanidae, Laridae, Mimidae, Nesospingidae, Oxyruncidae, Parulidae, Passerellidae, Phalacrocoracidae, Pipridae, Podicipedidae, Polioptilidae, Rallidae, Scolopacidae, Spindalidae, Strigidae, Thamnophilidae, Thraupidae, Threskiornithidae, Tityridae, Trochilidae, Troglodytidae, Turdidae, Tyrannidae, Vireonidae

Species: *Accipiter bicolor* (Bicolored Hawk), *Accipiter striatus* (Sharp-shinned Hawk), *Actitis macularius* (Spotted Sandpiper), *Adelomyia melanogenys* (Speckled Hummingbird), *Aglaiocercus coelestis* (Violet-tailed Sylph), *Amazilia tzacatl* (Rufous-tailed Hummingbird), *Amblycercus holosericeus* (Yellow-billed Cacique), *Anhinga anhinga* (Anhinga), *Anous stolidus* (Brown Noddy), *Aramus guarauna* (Limpkin), *Ardea alba* (Great Egret), *Ardea cocoi* (Cocoi Heron), *Arremon castaneiceps* (Olive Finch), *Attila spadiceus* (Bright-rumped Attila), *Basileuterus tristriatus* (Three-striped Warbler), *Boissonneaua flavescens* (Buff-tailed Coronet), *Bubulcus ibis* (Cattle Egret), *Buteo magnirostris* (Roadside Hawk), *Buteo platypterus* (Broad-winged Hawk), *Buteogallus urubitinga* (Great Black Hawk), *Butorides striata* (Striated Heron), *Calidris pusilla* (Semipalmated Sandpiper), *Calyptophilus frugivorus* (Eastern Chat-Tanager), *Calyptophilus tertius* (Western Chat-Tanager), *Campylorhamphus pusillus* (Brown-billed Scythebill), *Campylorhynchus rufinucha* (Rufous-naped Wren), *Caracara plancus* (Crested Caracara), *Catharus bicknelli* (Bicknell's Thrush), *Ceratopipra mentalis* (Red-capped Manakin), *Cercomacroides tyrannina* (Dusky Antbird), *Chloroceryle aenea* (American Pygmy Kingfisher), *Chloroceryle amazona* (Amazon Kingfisher), *Chloroceryle americana* (Green Kingfisher), *Chlorophanes spiza* (Green Honeycreeper), *Chlorospingus flavigularis* (Yellow-throated Chlorospingus), *Chlorothraupis stolzmanni* (Ochre-breasted Tanager), *Cinnycerthia olivascens* (Sharpe's Wren), *Cochlearius cochlearius* (Boat-billed Heron), *Coeligena coeligena* (Bronzy Inca), *Coeligena torquata* (Collared Inca), *Coeligena wilsoni* (Brown Inca), *Coereba flaveola* (Banaquit), *Colibri cyanotus* (Lesser Violetear), *Columbina buckleyi* (Ecuadorian Ground Dove), *Coragyps atratus* (Black Vulture), *Crotophaga sulcirostris* (Grove-billed Ani), *Cyanoloxia cyanoides* (Blue-black Grosbeak), *Daptrius ater* (Black Caracara), *Dendrocincla anabatina* (Tawny-winged Woodcreeper), *Dendrocincla homochroa* (Ruddy Woodcreeper), *Dendrocygna autumnalis* (Black-bellied Whistling-Duck), *Diglossa albilatera* (White-sided Flowerpiercer), *Doryfera ludovicae* (Green-fronted Lancebill), *Dumetella carolinensis* (Gray Catbird), *Egretta thula* (Snowy Egret), *Elaenia flavogaster* (Yellow-bellied Elaenia), *Empidonax flaviventris* (Yellow-bellied Flycatcher), *Eucometis penicillata* (Gray-headed Tanager), *Eudocimus ruber* (Scarlet Ibis), *Euphonia xanthogaster* (Orange-bellied Euphonia), *Fulica ardesiaca* (Slate-colored Coot), *Furnarius leucopus* (Pale-legged Hornero), *Geothlypis formosa* (Kentucky Warbler), *Geothlypis trichas* (Common Yellowthroat), *Geotrygon montana* (Ruddy Quail-Dove), *Glaucidium brasilianum* (Ferruginous Pygmy-Owl), *Glaucidium peruanum* (Peruvian Pygmy-Owl), *Grallaricula flavirostris* (Ochre-breasted Antpitta), *Granatellus sallaei* (Gray-throated Chat), *Habia cristata* (Crested Ant-Tanager), *Harpagus bidentatus* (Double-toothed Kite), *Heliangelus exortis* (Tourmaline Sunangel), *Heliodoxa imperatrix* (Empress Brilliant), *Heliodoxa rubinoides* (Fawn-breasted Brilliant), *Helmitheros vermivorum* (Worm-eating Warbler), *Henicorhina leucosticta* (White-breasted Wood-Wren), *Henicorhina negreti* (Munchique Wood-Wren), *Hylocichla mustelina* (Wood Thrush), *Icteria virens* (Yellow-breasted Chat), *Icterus galbula* (Baltimore Oriole), *Icterus pustulatus* (Streak-backed Oriole), *Icterus spurius* (Orchard Oriole), *Jacana spinosa* (Northern Jacana), *Leiothlypis peregrina* (Tennessee Warbler), *Leptopogon rufipectus* (Rufous-breasted Flycatcher), *Leucopternis kuhli* (White-browed Hawk), *Leucopternis melanops* (Black-faced Hawk), *Leucopternis semiplumbeus* (Semiplumbeous Hawk), *Manacus candei* (White-collared Manakin), *Manacus manacus* (White-bearded Manakin), *Margarops fuscatus* (Pearly-eyed Thrasher), *Margarornis squamiger* (Pearled Treerunner), *Mecocerculus calopterus* (Rufous-winged Tyrannulet), *Melopyrrha portoricensis* (Puerto

Rican Bullfinch), *Micrastur buckleyi* (Buckley's Forest-Falcon), *Micrastur gilvicollis* (Lined Forest-Falcon), *Micrastur mirandollei* (Slaty-backed Forest-Falcon), *Micrastur ruficollis* (Barred Forest-Falcon), *Micrastur semitorquatus* (Collared Forest-Falcon), *Microligea palustris* (Green-tailed Warbler), *Mionectes olivaceus* (Olive-streaked Flycatcher), *Mionectes striaticollis* (Streak-necked Flycatcher), *Mniotilta varia* (Black-and-white Warbler), *Myadestes ralloides* (Andean Solitaire), *Mycteria americana* (Wood Stork), *Myiarchus tyrannulus* (Brown-crested Flycatcher), *Myiobius sulphureipygius* (Sulphur-rumped Flycatcher), *Myiobius villosus* (Tawny-breasted Flycatcher), *Myioborus miniatus* (Slate-throated Redstart), *Myiozetetes similis* (Social Flycatcher), *Myrmotherula axillaris* (White-flanked Antwren), *Nannopterum brasilianum* (Neotropic Cormorant), *Nesospingus speculiferus* (Puerto Rican Tanager), *Nycticorax nycticorax* (Black-crowned Night-Heron), *Ochthoeca cinnamomeiventris* (Chestnut-bellied Chat-Tyrant), *Ocreatus underwoodii* (White-booted Racket-tail), *Oncostoma cinereigulare* (Northern Bentbill), *Onychoprion anaethetus* (Bridled Tern), *Onychoprion fuscatus* (Sooty Tern), *Pachyramphus aglaiae* (Rose-throated Becard), *Parkesia noveboracensis* (Northern Waterthrush), *Passerina ciris* (Painted Bunting), *Phaethornis syrmatophorus* (Tawny-bellied Hermit), *Pheugopedius maculipectus* (Spot-breasted Wren), *Phlegopsis nigromaculata* (Black-spotted Bare-eye), *Pipra fasciicauda* (Band-tailed Manakin), *Pitangus sulphuratus* (Great Kiskadee), *Pogonotriccus poecilotis* (Variegated Bristle-Tyrant), *Premnoplex brunnescens* (Spotted Barbtail), *Protonotaria citrea* (Prothonotary Warbler), *Pseudotriccus pelzelni* (Bronze-olive Pygmy-Tyrant), *Quiscalus mexicanus* (Great-tailed Grackle), *Ramphocaenus melanurus* (Long-billed Gnatwren), *Ramphocelus flammigerus* (Flame-rumped Tanager), *Rollandia rolland* (White-tufted Grebe), *Rostrhamus sociabilis* (Snail Kite), *Seiurus aurocapilla* (Ovenbird), *Setophaga citrina* (Hooded Warbler), *Setophaga magnolia* (Magnolia Warbler), *Setophaga petechia* (Yellow Warbler), *Setophaga ruticilla* (American Redstart), *Snowornis cryptolophus* (Olivaceous Piha), *Spindalis portoricensis* (Puerto Rican Spindalis), *Sporophila luctuosa* (Black-and-white Seedeater), *Sporophila morelleti* (Morelet's Seedeater), *Sterna dougallii* (Roseate Tern), *Synallaxis erythrothorax* (Rufous-breasted Spinetail), *Tachycineta albilinea* (Mangrove Swallow), *Tangara icterocephala* (Silver-throated Tanager), *Thalurania colombica* (Crowned Woodnymph), *Threnetes ruckeri* (Band-tailed Barbthroat), *Thripadectes flammulatus* (Flammulated Treehunter), *Thripadectes holostictus* (Striped Treehunter), *Thripadectes ignobilis* (Uniform Treehunter), *Thryophilus rufalbus* (Rufous-and-white Wren), *Todirostrum cinereum* (Common Tody-Flycatcher), *Tolmomyias sulphurescens* (Yellow-olive Flycatcher), *Turdus maculirostris* (Ecuadorian Thrush), *Turdus plumbeus* (Red-legged Thrush), *Turdus serranus* (Glossy-black Thrush), *Turdus swalesi* (La Selle Thrush), *Tyrannus melancholicus* (Tropical Kingbird), *Uropsila leucogastra* (White-bellied Wren), *Vermivora cyanoptera* (Blue-winged Warbler), *Vireo altiloquus* (Black-whiskered Vireo), *Vireo flavoviridis* (Yellow-green Vireo), *Vireo griseus* (White-eyed Vireo), *Vireo pallens* (Mangrove Vireo), *Volatinia jacarina* (Blue-black Grassquit), *Xenoligea montana* (White-winged Warbler), *Xiphorhynchus triangularis* (Olive-backed Woodcreeper)

Table S2. Total mercury (THg) concentrations (μg/g) among all 41 sites and nine countries sampled across Central America, South America, and the West Indies from 2007–2023. Sampling duration, number of species sampled, sample size (*n*), arithmetic mean ± standard deviation (SD), range, and coefficient of variation (CV) are summarized by country, site, dominant habitat, and tissue type. Sites are arranged alphabetically by country. An asterisks (*) indicates that there is artisanal gold mining present within a 7 km radius of the site, and a dash (–) indicates there are no data to report.

Country	Site (latitude, longitude)	Dominant habitats	Sampled tissue	Sampling duration	Species sampled	*n*	Arithmetic mean ± SD	Range	CV
Belize	—	—	Whole blood	2007–2021	86	583	0.143 ± 0.270	0.001–3.195	189.0%
			Body feather	2019–2021	52	162	1.487 ± 3.529	0.001–19.596	237.4%
			Tail feather	2007–2019	54	201	1.386 ± 3.321	0.001–25.841	239.5%
	Belize Foundation for Research & Environmental Education (16.55578, –88.70777)	Secondary forest	Whole blood	2010–2011	13	65	0.155 ± 0.402	0.010–3.195	258.9%
	Monkey Bay Wildlife Sanctuary (17.30258, –88.55478)	Riparian thickets	Whole blood	2010–2021	25	52	0.109 ± 0.155	0.001–0.741	142.3%
			Body feather	2021	10	13	1.646 ± 3.798	0.001–13.432	230.7%
			Tail feather	2021	7	9	1.167 ± 2.727	0.001–8.382	233.7%
	Runaway Creek Nature Reserve (17.313222, –88.460329)	Secondary forest; low, seasonally wet grassland; second-growth scrub; tropical	Whole blood	2007–2021	69	334	0.168 ± 0.282	0.001–2.659	168.4%
			Body feather	2019–2021	38	93	1.194 ± 2.513	0.004–18.237	210.5%
			Tail feather	2007–2021	46	139	1.309 ± 2.938	0.008–23.028	224.4%

		lowland evergreen forest							
	Toucan Ridge Ecology and Education Society (17.05231, –88.56773)	Secondary forest; tropical lowland evergreen forest	Whole blood	2021	14	28	0.031 ± 0.049	0.001–0.138	129.0%
			Body feather	2021	9	16	0.558 ± 0.562	0.052–1.878	100.9%
			Tail feather	2021	7	14	0.561 ± 0.423	0.189–1.717	75.4%
	Tropical Education Center (17.35812, –88.54162)	Secondary forest; pine-oak forest	Whole blood	2009–2021	42	104	0.103 ± 0.186	0.001–1.187	179.8%
			Body feather	2019–2021	19	40	2.487 ± 5.510	0.003–19.596	221.5%
			Tail feather	2007–2021	16	39	2.009 ± 4.939	0.014–25.841	245.8%
Colombia	—	—	Whole blood	2023	21	31	0.523 ± 0.684	0.001–3.112	130.7%
			Body feather	2023	22	32	6.695 ± 6.625	0.148–20.594	98.9%
	* Humedal Ayapel (8.32107, –75.12836)	Freshwater marshes	Whole blood	2023	21	31	0.523 ± 0.684	0.001–3.112	130.7%
			Body feather	2023	22	32	6.695 ± 6.625	0.148–20.594	98.9%
Costa Rica	—	—	Whole blood	2010–2013	21	66	0.049 ± 0.068	0.001–0.322	139.5%
			Tail feather	2010–2011	32	70	0.500 ± 1.393	0.001–11.291	278.6%
	Barra del Colorado Wildlife Refuge (10.59456, –83.52860)	Flooded tropical evergreen forest	Tail feather	2010	10	21	0.916 ± 2.425	0.021–11.291	264.6%
	Centro Agronómico Tropical de Investigación y Enseñanza (9.898825, –83.657081)	Secondary forest	Whole blood	2010	1	1	0.085	—	—
			Tail feather	2010	4	5	0.171 ± 0.184	0.064–0.495	107.7%
	Instituto Nacional de Biodiversidad (9.97349, –84.09309)	Secondary forest	Tail feather	2010	4	13	0.139 ± 0.082	0.033–0.352	58.9%
	La Selva Biological Station (10.43061, –84.00718)	Tropical lowland evergreen forest	Whole blood	2010–2013	11	52	0.059 ± 0.073	0.004–0.322	123.3%
	Reserva Playa Tortuga (9.07462, –83.65910)	Flooded tropical evergreen forest	Whole blood	2011	9	13	0.006 ± 0.011	0.004–0.034	192.9%
			Tail feather	2011	15	21	0.197 ± 0.344	0.001–1.299	175.2%
Dominican Republic	—	—	Whole blood	2017	35	124	0.109 ± 0.109	0.001–0.665	100.1%
			Body feather	2018	1	5	0.801 ± 0.609	0.359–1.653	76.1%
			Tail feather	2018	1	5	0.717 ± 0.495	0.295–1.337	69.0%
	Jardín Botánico (18.49434, –69.95351)	Secondary forest	Whole blood	2017	16	27	0.072 ± 0.098	0.001–0.394	135.3%
			Body feather	2018	1	5	0.801 ± 0.609	0.359–1.653	76.1%
			Tail feather	2018	1	5	0.717 ± 0.495	0.295–1.337	69.0%
	Parque Nacional Manglares de Bajo Yuna (19.21612, –69.63405)	Mangrove forest	Whole blood	2017	20	81	0.142 ± 0.107	0.015–0.665	75.5%

	Plaza de Cultura Juan Pablo Duarte (18.47138, –69.90839)	Secondary forest	Whole blood	2017	7	13	0.006 ± 0.013	0.001–0.048	215.9%
	Monumento Ruinas de San Francisco (18.47668, –69.88643)	Secondary forest	Whole blood	2017	2	3	0.001 ± 0.000	0.001–0.001	0.0%
Mexico	—	—	Whole blood	2013	9	17	0.085 ± 0.093	0.008–0.343	109.2%
			Tail feather	2013	3	7	0.807 ± 1.029	0.025–3.019	127.6%
	Barra Vieja (16.68783, –99.62450)	Secondary forest	Whole blood	2013	1	7	0.032 ± 0.009	0.018–0.047	27.7%
	Isla Ixtapa (17.67822, –101.65613)	Tropical deciduous forest	Whole blood	2013	1	1	0.162	—	—
			Tail feather	2013	1	2	2.011 ± 1.426	1.003–3.019	70.9%
	Laguna de Tres Palos (16.71292, –99.66055)	Freshwater marshes	Whole blood	2013	1	1	0.343	—	—
	Paraíso Escondido (17.08120, –100.48476)	Tropical deciduous forest	Whole blood	2013	5	7	0.081 ± 0.084	0.008–0.257	103.0%
			Tail feather	2013	2	5	0.325 ± 0.258	0.025–0.550	79.3%
	Punta Potosí (17.53366,–101 .44352)	Mangrove forest	Whole blood	2013	1	1	0.152	—	—
Nicaragua	—	—	Whole blood	2012–2014	37	111	0.087 ± 0.145	0.001–0.699	166.3%
			Tail feather	2010–2014	44	150	0.805 ± 1.242	0.001–12.070	154.2%
	El Guayabo (12.03192, –85.92570)	Freshwater marshes	Whole blood	2012	6	12	0.023 ± 0.03	0.006–0.114	131.1%
			Tail feather	2012	2	4	0.245 ± 0.113	0.112–0.367	46.2%
	Finca Linda Vista (13.14709, –85.95876)	Secondary forest	Whole blood	2014	3	4	0.104 ± 0.144	0.001–0.311	139.2%
			Tail feather	2014	4	7	0.221 ± 0.438	0.006–1.202	198.7%
	Punta Tirurí (11.1884, –85.48998)	Freshwater marshes; gallery forest	Whole blood	2012	15	34	0.072 ± 0.087	0.003–0.417	121.3%
			Tail feather	2012	3	5	1.009 ± 0.499	0.396–1.559	49.5%
	Refugio de Vida Silvestre La Flor (11.13978, –85.78932)	Tropical deciduous forest	Tail feather	2014	4	15	1.155 ± 0.718	0.063–2.407	62.2%
	Reserva El Jaguar (13.24397, –86.05295)	Secondary forest	Tail feather	2010	28	72	0.526 ± 0.559	0.010–2.547	106.3%
	Reserva Natural Laguna de Tisma (12.08867, –85.98433)	Freshwater marshes	Whole blood	2014	3	7	0.033 ± 0.031	0.004–0.086	95.9%
			Tail feather	2014	2	3	0.009 ± 0.007	0.001–0.014	77.5%
	Río Brito (11.34715, –85.97828)	Tropical deciduous forest	Whole blood	2014	6	7	0.106 ± 0.089	0.020–0.254	83.4%
			Tail feather	2014	3	3	2.711 ± 2.317	0.213–4.789	85.5%
	Río Escondido (11.25992, –85.87382)	Tropical deciduous forest	Whole blood	2012	3	3	0.058 ± 0.051	0.014–0.114	88.0%
			Tail feather	2012	2	2	0.886 ± 0.724	0.374–1.398	81.7%
	Río Jigüina (13.13153, –85.92920)	Tropical deciduous forest	Whole blood	2014	8	12	0.367 ± 0.267	0.011–0.699	72.6%
			Tail feather	2014	5	10	1.591 ± 3.740	0.010–12.070	235.1%
	Río La Escamequita (11.19233,	Tropical deciduous forest	Whole blood	2012	2	2	0.047 ± 0.038	0.020–0.074	81.2%
			Tail feather	2012	1	1	1.701	—	—

	–85.81776)								
	Río Ostional (11.10891, –85.76067)	Tropical deciduous forest	Whole blood	2014	9	13	0.036 ± 0.043	0.001–0.122	118.2%
			Tail feather	2014	8	26	1.159 ± 1.019	0.068–3.446	87.9%
	Río Tipitapa (12.20296, –85.10172)	Tropical deciduous forest	Whole blood	2012	9	17	0.027 ± 0.025	0.003–0.073	95.1%
			Tail feather	2012	1	2	0.187 ± 0.116	0.105–0.269	62.0%
Panama	—	—	Tail feather	2011	6	17	2.720 ± 1.627	0.414–5.738	59.8%
	Juan Díaz (9.01956, –79.44658)	Flooded tropical evergreen forest	Tail feather	2011	6	17	2.720 ± 1.627	0.414–5.738	59.8%
Peru	—	—	Body feather	2009–2019	128	464	2.911 ± 6.762	0.027–72.795	232.3%
			Tail feather	2017–2019	75	212	1.469 ± 1.666	0.056–8.606	113.4%
	Cocha Cashu Biological Station (–11.88829, –71.40757)	Flooded tropical evergreen forest	Tail feather	2019	14	18	0.843 ± 0.860	0.061–3.411	102.1%
	* Los Amigos Biological Station (–12.56940, –70.10009)	Tropical lowland evergreen forest; flooded tropical evergreen forest	Body feather	2009–2019	45	175	2.452 ± 2.783	0.100–19.887	113.5%
			Tail feather	2017–2019	71	194	1.527 ± 1.711	0.056–8.606	112.0%
	* Reserva Nacional Tambopata - Azul (–13.04653, –69.92719)	Tropical lowland evergreen forest	Body feather	2019	27	45	3.266 ± 6.287	0.155–30.681	192.5%
	* Laberinto (–12.68105, –69.62522)	Secondary forest	Body feather	2019	50	120	4.295 ± 9.528	0.123–66.279	221.9%
	* Inambari (–12.91890, –70.24214)	Secondary forest	Body feather	2019	35	72	3.262 ± 9.701	0.027–72.795	297.4%
	Reserva Nacional Tambopata - La Torre (–12.85130, –69.29888)	Tropical lowland evergreen forest	Body feather	2019	27	52	0.469 ± 0.417	0.038–1.889	88.9%
Puerto Rico	—	—	Whole blood	2010	8	31	0.006 ± 0.006	0.001–0.032	104.6%
			Tail feather	2010	6	28	0.118 ± 0.101	0.031–0.533	85.8%
	El Yunque National Forest (18.30090, –65.78775)	Elfin forest	Whole blood	2010	8	31	0.006 ± 0.006	0.001–0.032	104.6%
			Tail feather	2010	6	28	0.118 ± 0.101	0.031–0.533	85.8%

Table S3. Taxonomic, trophic niche, primary habitat association, and migratory status classifications assigned to all 317 Neotropical bird species sampled across Central America, South America, and the West Indies from 2007–2023. Species are arranged alphabetically by order and family. A dash (–) indicates there are no data to report.

Order	Family	Common name	Latin name	Trophic level	Trophic niche	Primary habitat	Migratory status
Accipitriformes	Accipitridae (Hawks, Eagles, and Kites)	Bicolored Hawk	*Accipiter bicolor*	Carnivore	Terrestrial vertivore	Tropical deciduous forest	Resident
		Black-faced Hawk	*Leucopternis*	Carnivore	Terrestrial vertivore	Tropical lowland	Resident

			melanops			evergreen forest	
		Great Black Hawk	*Buteogallus urubitinga*	Carnivore	Terrestrial vertivore	Tropical deciduous forest	Resident
		Ornate Hawk-Eagle	*Spizaetus ornatus*	Carnivore	Terrestrial vertivore	Tropical lowland evergreen forest	Resident
		Roadside Hawk	*Rupornis magnirostris*	Carnivore	Terrestrial vertivore	Tropical lowland evergreen forest	Resident
		Slate-colored Hawk	*Buteogallus schistaceus*	Carnivore	Terrestrial vertivore	Flooded tropical evergreen forest	Resident
		White-browed Hawk	*Leucopternis kuhli*	Carnivore	Terrestrial vertivore	Tropical lowland evergreen forest	Resident
Anseriformes	Anatidae (Ducks, Geese, and Waterfowl)	West Indian Whistling-Duck	*Dendrocygna arborea*	Herbivore	Omnivore	Freshwater marshes	Resident
Caprimulgiformes	Apodidae (Swifts)	Antillean Palm-Swift	*Tachornis phoenicobia*	Carnivore	Invertivore	Low, seasonally wet grassland	Resident
	Caprimulgidae (Nightjars and Allies)	Common Pauraque	*Nyctidromus albicollis*	Carnivore	Invertivore	Tropical lowland evergreen forest	Resident
		Ocellated Poorwill	*Nyctiphrynus ocellatus*	Carnivore	Invertivore	Tropical lowland evergreen forest	Resident
	Trochilidae (Hummingbirds)	Blue-tailed Emerald	*Chlorostilbon mellisugus*	Herbivore	Nectarivore	Montane evergreen forest	Resident
		Fork-tailed Woodnymph	*Thalurania furcata*	Herbivore	Nectarivore	Tropical lowland evergreen forest	Resident
		Great-billed Hermit	*Phaethornis malaris*	Herbivore	Nectarivore	Tropical lowland evergreen forest	Resident
		Pale-tailed Barbthroat	*Threnetes leucurus*	Herbivore	Nectarivore	Tropical lowland evergreen forest	Resident
		Reddish Hermit	*Phaethornis ruber*	Herbivore	Nectarivore	Tropical lowland evergreen forest	Resident
		Rufous-breasted Hermit	*Glaucis hirsutus*	Herbivore	Nectarivore	Tropical lowland evergreen forest	Resident
		Sapphire-spangled Emerald	*Chionomesa lactea*	Herbivore	Nectarivore	Tropical lowland evergreen forest	Resident
		White-bearded Hermit	*Phaethornis hispidus*	Herbivore	Nectarivore	Flooded tropical evergreen forest	Resident
Charadriiformes	Jacanidae (Jacanas)	Northern Jacana	*Jacana spinosa*	Carnivore	Aquatic predator	Freshwater marshes	Resident
		Wattled Jacana	*Jacana jacana*	Omnivore	Omnivore	Freshwater marshes	Resident
	Laridae (Gulls, Terns, and Skimmers)	Laughing Gull	*Leucophaeus atricilla*	Carnivore	Aquatic predator	Coastal waters	Partial migrant
	Scolopacidae (Sandpipers and Allies)	Least Sandpiper	*Calidris minutilla*	Carnivore	Aquatic predator	Freshwater marshes	Full migrant
		Solitary Sandpiper	*Tringa solitaria*	Carnivore	Aquatic predator	Freshwater lakes and ponds	Full migrant
		Spotted Sandpiper	*Actitis macularius*	Carnivore	Aquatic predator	Riverine sand beaches	Full migrant
Columbiformes	Columbidae (Pigeons and Doves)	Common Ground Dove	*Columbina passerina*	Herbivore	Omnivore	Arid lowland scrub	Resident
		Gray-fronted Dove	*Leptotila rufaxilla*	Herbivore	Granivore	River-edge forest	Resident
		Gray-headed Dove	*Leptotila plumbeiceps*	Herbivore	Granivore	Tropical lowland evergreen forest	Resident
		Inca Dove	*Columbina inca*	Herbivore	Granivore	Second-growth scrub	Resident
		Mourning Dove	*Zenaida macroura*	Herbivore	Granivore	Pastures/agricultural lands	Resident
		Rock Pigeon	*Columba livia*	Herbivore	Granivore	Second-growth scrub	Resident

		Ruddy Ground Dove	*Columbina talpacoti*	Herbivore	Granivore	Second-growth scrub	Resident
		Ruddy Quail-Dove	*Geotrygon montana*	Herbivore	Omnivore	Tropical lowland evergreen forest	Resident
		White-tipped Dove	*Leptotila verreauxi*	Herbivore	Granivore	Tropical deciduous forest	Resident
Coraciiformes	Alcedinidae (Kingfishers)	Amazon Kingfisher	*Chloroceryle amazona*	Carnivore	Aquatic predator	Rivers	Resident
		American Pygmy Kingfisher	*Chloroceryle aenea*	Carnivore	Aquatic predator	Streams	Resident
		Green Kingfisher	*Chloroceryle americana*	Carnivore	Aquatic predator	Streams	Resident
		Green-and-rufous Kingfisher	*Chloroceryle inda*	Carnivore	Aquatic predator	Streams	Resident
		Ringed Kingfisher	*Megaceryle torquata*	Carnivore	Aquatic predator	Rivers	Resident
	Momotidae (Motmots)	Amazonian Motmot	*Momotus momota*	Carnivore	Omnivore	Tropical lowland evergreen forest	Resident
		Lesson's Motmot	*Momotus lessonii*	Carnivore	Omnivore	Tropical lowland evergreen forest	Resident
		Rufous Motmot	*Baryphthengus martii*	Carnivore	Omnivore	Tropical lowland evergreen forest	Resident
	Todidae (Todies)	Broad-billed Tody	*Todus subulatus*	Carnivore	Invertivore	Tropical deciduous forest	Resident
Cuculiformes	Cuculidae (Cuckoos)	Groove-billed Ani	*Crotophaga sulcirostris*	Carnivore	Invertivore	Second-growth scrub	Resident
		Hispaniolan Lizard-Cuckoo	*Coccyzus longirostris*	Carnivore	Invertivore	Tropical deciduous forest	Resident
		Little Cuckoo	*Coccycua minuta*	Carnivore	Invertivore	Tropical lowland evergreen forest	Resident
		Smooth-billed Ani	*Crotophaga ani*	Carnivore	Omnivore	Second-growth scrub	Resident
Falconiformes	Falconidae (Falcons and Caracaras)	Barred Forest-Falcon	*Micrastur ruficollis*	Carnivore	Terrestrial vertivore	Tropical lowland evergreen forest	Resident
		Bat Falcon	*Falco rufigularis*	Carnivore	Terrestrial vertivore	Tropical lowland evergreen forest	Resident
		Black Caracara	*Daptrius ater*	Carnivore	Omnivore	River-edge forest	Resident
		Buckley's Forest-Falcon	*Micrastur buckleyi*	Carnivore	Terrestrial vertivore	Tropical lowland evergreen forest	Resident
		Collared Forest-Falcon	*Micrastur semitorquatus*	Carnivore	Terrestrial vertivore	Tropical lowland evergreen forest	Resident
		Lined Forest-Falcon	*Micrastur gilvicollis*	Carnivore	Terrestrial vertivore	Tropical lowland evergreen forest	Resident
		Slaty-backed Forest-Falcon	*Micrastur mirandollei*	Carnivore	Terrestrial vertivore	Tropical lowland evergreen forest	Resident
Galbuliformes	Bucconidae (Puffbirds)	Black-fronted Nunbird	*Monasa nigrifrons*	Carnivore	Invertivore	Flooded tropical evergreen forest	Resident
		Chestnut-capped Puffbird	*Bucco macrodactylus*	Carnivore	Invertivore	River-edge forest	Resident
		Semicollared Puffbird	*Malacoptila semicincta*	Carnivore	Invertivore	Tropical lowland evergreen forest	Resident
		White-whiskered Puffbird	*Malacoptila panamensis*	Carnivore	Invertivore	Tropical lowland evergreen forest	Resident
	Galbulidae (Jacamars)	Bluish-fronted Jacamar	*Galbula cyanescens*	Carnivore	Invertivore	Tropical lowland evergreen forest	Resident
		Rufous-tailed Jacamar	*Galbula ruficauda*	Carnivore	Invertivore	Tropical lowland evergreen forest	Resident
Passeriformes	Cardinalidae (Cardinals and Allies)	Blue Bunting	*Cyanocompsa parellina*	Herbivore	Frugivore	Tropical deciduous forest	Resident
		Blue-black Grosbeak	*Cyanoloxia*	Herbivore	Granivore	Tropical lowland	Resident

		cyanoides			evergreen forest	
	Gray-throated Chat	*Granatellus sallaei*	Carnivore	Invertivore	Tropical deciduous forest	Resident
	Hepatic Tanager	*Piranga flava*	Carnivore	Invertivore	Pine-Oak forest	Resident
	Painted Bunting	*Passerina ciris*	Herbivore	Granivore	Second-growth scrub	Partial migrant
	Red-crowned Ant-Tanager	*Habia rubica*	Carnivore	Invertivore	Tropical lowland evergreen forest	Resident
	Red-throated Ant-Tanager	*Habia fuscicauda*	Carnivore	Invertivore	Tropical lowland evergreen forest	Resident
Conopophagidae (Gnateaters)	Ash-thoated Gnateater	*Conopophaga peruviana*	Carnivore	Invertivore	Tropical lowland evergreen forest	Resident
Corvidae (Crows, Jays, and Magpies)	Brown Jay	*Psilorhinus morio*	Omnivore	Omnivore	Gallery forest	Resident
	Purplish Jay	*Cyanocorax cyanomelas*	Omnivore	Omnivore	Tropical deciduous forest	Resident
	White-throated Magpie-Jay	*Calocitta formosa*	Omnivore	Omnivore	Tropical deciduous forest	Resident
Dulidae (Palmchat)	Palmchat	*Dulus dominicus*	Herbivore	Frugivore	Secondary forest	Resident
Formicariidae (Antthrushes)	Black-faced Antthrush	*Formicarius analis*	Carnivore	Invertivore	Tropical lowland evergreen forest	Resident
	Rufous-fronted Antthrush	*Formicarius rufifrons*	Carnivore	Invertivore	River-edge forest	Resident
Fringillidae (Finches, Euphonias, and Allies)	Olive-backed Euphonia	*Euphonia gouldi*	Herbivore	Frugivore	Tropical lowland evergreen forest	Resident
	Orange-bellied Euphonia	*Euphonia xanthogaster*	Herbivore	Frugivore	Montane evergreen forest	Resident
	Yellow-throated Euphonia	*Euphonia hirundinacea*	Herbivore	Frugivore	Tropical lowland evergreen forest	Resident
Furnariidae (Ovenbirds and Woodcreepers)	Black-banded Woodcreeper	*Dendrocolaptes picumnus*	Carnivore	Invertivore	Tropical lowland evergreen forest	Resident
	Buff-throated Foliage-gleaner	*Automolus ochrolaemus*	Carnivore	Invertivore	Tropical lowland evergreen forest	Resident
	Buff-throated Woodcreeper	*Xiphorhynchus guttatus*	Carnivore	Invertivore	Tropical lowland evergreen forest	Resident
	Chestnut-crowned Foliage-gleaner	*Automolus rufipileatus*	Carnivore	Invertivore	River-edge forest	Resident
	Chestnut-winged Hookbill	*Ancistrops strigilatus*	Carnivore	Invertivore	Tropical lowland evergreen forest	Resident
	Cinnamon-rumped Foliage-gleaner	*Philydor pyrrhodes*	Carnivore	Invertivore	Tropical lowland evergreen forest	Resident
	Cocoa Woodcreeper	*Xiphorhynchus susurrans*	Carnivore	Invertivore	Tropical lowland evergreen forest	Resident
	Elegant Woodcreeper	*Xiphorhynchus elegans*	Carnivore	Invertivore	Tropical lowland evergreen forest	Resident
	Ivory-billed Woodcreeper	*Xiphorhynchus flavigaster*	Carnivore	Invertivore	Tropical lowland evergreen forest	Resident
	Long-tailed Woodcreeper	*Deconychura longicauda*	Carnivore	Invertivore	Tropical lowland evergreen forest	Resident
	Northern Barred-Woodcreeper	*Dendrocolaptes sanctithomae*	Carnivore	Invertivore	Tropical lowland evergreen forest	Resident
	Ocellated Woodcreeper	*Xiphorhynchus ocellatus*	Carnivore	Invertivore	Tropical lowland evergreen forest	Resident
	Olivaceous Woodcreeper	*Sittasomus griseicapillus*	Carnivore	Invertivore	Tropical lowland evergreen forest	Resident
	Olive-backed Foliage-gleaner	*Automolus infuscatus*	Carnivore	Invertivore	Tropical lowland evergreen forest	Resident
	Pale-legged Hornero	*Furnarius leucopus*	Carnivore	Invertivore	Second-growth	Resident

					scrub	
	Plain Xenops	*Xenops minutus*	Carnivore	Invertivore	Tropical lowland evergreen forest	Resident
	Plain-brown Woodcreeper	*Dendrocincla fuliginosa*	Carnivore	Invertivore	Tropical lowland evergreen forest	Resident
	Plain-crowned Spinetail	*Synallaxis gujanensis*	Carnivore	Invertivore	River-edge forest	Resident
	Red-billed Scythebill	*Campylorhamphus trochilirostris*	Carnivore	Invertivore	Tropical lowland evergreen forest	Resident
	Ruddy Treerunner	*Margarornis rubiginosus*	Carnivore	Invertivore	Montane evergreen forest	Resident
	Ruddy Woodcreeper	*Dendrocincla homochroa*	Carnivore	Invertivore	Tropical lowland evergreen forest	Resident
	Rufous-breasted Spinetail	*Synallaxis erythrothorax*	Carnivore	Invertivore	Secondary forest	Resident
	Spot-crowned Woodcreeper	*Lepidocolaptes affinis*	Carnivore	Invertivore	Montane evergreen forest	Resident
	Straight-billed Woodcreeper	*Dendroplex picus*	Carnivore	Invertivore	River-edge forest	Resident
	Streak-headed Woodcreeper	*Lepidocolaptes souleyetii*	Carnivore	Invertivore	Tropical deciduous forest	Resident
	Striped Woodcreeper	*Xiphorhynchus obsoletus*	Carnivore	Invertivore	Flooded tropical evergreen forest	Resident
	Tawny-winged Woodcreeper	*Dendrocincla anabatina*	Carnivore	Invertivore	Tropical lowland evergreen forest	Resident
	Wedge-billed Woodcreeper	*Glyphorynchus spirurus*	Carnivore	Invertivore	Tropical lowland evergreen forest	Resident
	White-chinned Woodcreeper	*Dendrocincla merula*	Carnivore	Invertivore	Tropical lowland evergreen forest	Resident
	Yellow-chinned Spinetail	*Certhiaxis cinnamomeus*	Carnivore	Invertivore	Freshwater marshes	Resident
Hirundinidae (Swallows)	Barn Swallow	*Hirundo rustica*	Carnivore	Invertivore	Pastures/agricultural lands	Partial migrant
	Brown-chested Martin	*Progne tapera*	Carnivore	Invertivore	Low, seasonally wet grassland	Resident
	Mangrove Swallow	*Tachycineta albilinea*	Carnivore	Invertivore	Mangrove forest	Resident
	Northern Rough-winged Swallow	*Stelgidopteryx serripennis*	Carnivore	Invertivore	Second-growth scrub	Partial migrant
	Southern Rough-winged Swallow	*Stelgidopteryx ruficollis*	Carnivore	Invertivore	Second-growth scrub	Resident
	White-banded Swallow	*Atticora fasciata*	Carnivore	Invertivore	Rivers	Resident
Icteridae (Troupials and Allies)	Baltimore Oriole	*Icterus galbula*	Omnivore	Invertivore	Secondary forest	Full migrant
	Black-cowled Oriole	*Icterus prosthemelas*	Omnivore	Omnivore	Tropical lowland evergreen forest	Resident
	Great-tailed Grackle	*Quiscalus mexicanus*	Omnivore	Omnivore	Second-growth scrub	Resident
	Melodious Blackbird	*Dives dives*	Omnivore	Omnivore	Tropical lowland evergreen forest	Resident
	Orchard Oriole	*Icterus spurius*	Carnivore	Invertivore	Secondary forest	Partial migrant
	Streak-backed Oriole	*Icterus pustulatus*	Omnivore	Invertivore	Tropical deciduous forest	Resident
	Yellow-billed Cacique	*Amblycercus holosericeus*	Carnivore	Invertivore	Montane evergreen forest	Resident
	Yellow-breasted Chat	*Icteria virens*	Carnivore	Invertivore	Secondary forest	Partial migrant

Mimidae (Mockingbirds and Thrashers)	Gray Catbird	*Dumetella carolinensis*	Omnivore	Invertivore	Tropical lowland evergreen forest	Full migrant
	Northern Mockingbird	*Mimus polyglottos*	Omnivore	Omnivore	Arid lowland scrub	Resident
	Pearly-eyed Thrasher	*Margarops fuscatus*	Omnivore	Omnivore	Arid lowland scrub	Resident
Nesospingidae (Puerto Rican Tanager)	Puerto Rican Tanager	*Nesospingus speculiferus*	Carnivore	Invertivore	Montane evergreen forest	Resident
Oxyruncidae (Sharpbill, Royal Flycatcher, and Allies)	Royal Flycatcher	*Onychorhynchus coronatus*	Carnivore	Invertivore	Tropical lowland evergreen forest	Resident
	Ruddy-tailed Flycatcher	*Terenotriccus erythrurus*	Carnivore	Invertivore	Tropical lowland evergreen forest	Resident
Parulidae (New World Warblers)	American Redstart	*Setophaga ruticilla*	Carnivore	Invertivore	Tropical lowland evergreen forest	Full migrant
	Black-and-white Warbler	*Mniotilta varia*	Carnivore	Invertivore	Tropical lowland evergreen forest	Full migrant
	Cape May Warbler	*Setophaga tigrina*	Omnivore	Invertivore	Tropical lowland evergreen forest	Full migrant
	Common Yellowthroat	*Geothlypis trichas*	Carnivore	Invertivore	Riparian thickets	Partial migrant
	Connecticut Warbler	*Oporornis agilis*	Carnivore	Invertivore	River-edge forest	Full migrant
	Flame-throated Warbler	*Oreothlypis gutturalis*	Carnivore	Invertivore	Montane evergreen forest	Resident
	Golden-crowned Warbler	*Basileuterus culicivorus*	Carnivore	Invertivore	Tropical lowland evergreen forest	Resident
	Hooded Warbler	*Setophaga citrina*	Carnivore	Invertivore	Tropical lowland evergreen forest	Full migrant
	Kentucky Warbler	*Geothlypis formosa*	Carnivore	Invertivore	Tropical lowland evergreen forest	Full migrant
	Louisiana Waterthrush	*Parkesia motacilla*	Carnivore	Invertivore	Tropical lowland evergreen forest	Full migrant
	Magnolia Warbler	*Setophaga magnolia*	Carnivore	Invertivore	Tropical lowland evergreen forest	Full migrant
	Mourning Warbler	*Geothlypis philadelphia*	Carnivore	Invertivore	Tropical lowland evergreen forest	Full migrant
	Northern Parula	*Setophaga americana*	Carnivore	Invertivore	Tropical lowland evergreen forest	Full migrant
	Northern Waterthrush	*Parkesia noveboracensis*	Carnivore	Invertivore	Tropical lowland evergreen forest	Full migrant
	Ovenbird	*Seiurus aurocapilla*	Carnivore	Invertivore	Tropical lowland evergreen forest	Full migrant
	Prairie Warbler	*Setophaga discolor*	Carnivore	Invertivore	Arid lowland scrub	Full migrant
	Prothonotary Warbler	*Protonotaria citrea*	Carnivore	Invertivore	Mangrove forest	Full migrant
	Rufous-capped Warbler	*Basileuterus rufifrons*	Carnivore	Invertivore	Tropical deciduous forest	Resident
	Swainson's Warbler	*Limnothlypis swainsonii*	Carnivore	Invertivore	Tropical lowland evergreen forest	Full migrant
	Tennessee Warbler	*Leiothlypis peregrina*	Carnivore	Invertivore	Secondary forest	Full migrant
	Worm-eating Warbler	*Helmitheros vermivorum*	Carnivore	Invertivore	Tropical lowland evergreen forest	Full migrant
	Yellow Warbler	*Setophaga petechia*	Carnivore	Invertivore	Gallery forest	Partial migrant
Passerellidae (New World Sparrows)	Chestnut-capped Brush-Finch	*Arremon brunneinucha*	Omnivore	Invertivore	Montane evergreen forest	Resident
	Common Chlorospingus	*Chlorospingus flavopectus*	Carnivore	Invertivore	Montane evergreen forest	Resident

	Green-backed Sparrow	*Arremonops chloronotus*	Herbivore	Omnivore	Tropical lowland evergreen forest	Resident
	Olive Sparrow	*Arremonops rufivirgatus*	Omnivore	Omnivore	Tropical deciduous forest	Resident
	Orange-billed Sparrow	*Arremon aurantiirostris*	Herbivore	Omnivore	Tropical lowland evergreen forest	Resident
	Pectoral Sparrow	*Arremon taciturnus*	Herbivore	Omnivore	Tropical lowland evergreen forest	Resident
	Rufous-collared Sparrow	*Zonotrichia capensis*	Herbivore	Omnivore	Arid lowland scrub	Resident
	White-naped Brush-Finch	*Atlapetes albinucha*	Omnivore	Omnivore	Montane evergreen forest	Resident
	Yellow-browed Sparrow	*Ammodramus aurifrons*	Herbivore	Granivore	River island scrub	Resident
Phaenicophilidae (Hispaniolan Tanagers)	Black-crowned Palm-Tanager	*Phaenicophilus palmarum*	Carnivore	Invertivore	Secondary forest	Resident
Pipridae (Manakins)	Band-tailed Manakin	*Pipra fasciicauda*	Herbivore	Frugivore	Flooded tropical evergreen forest	Resident
	Blue-crowned Manakin	*Lepidothrix coronata*	Herbivore	Frugivore	Tropical lowland evergreen forest	Resident
	Fiery-capped Manakin	*Machaeropterus pyrocephalus*	Herbivore	Frugivore	Tropical lowland evergreen forest	Resident
	Red-capped Manakin	*Ceratopipra mentalis*	Herbivore	Frugivore	Tropical lowland evergreen forest	Resident
	Round-tailed Manakin	*Ceratopipra chloromeros*	Herbivore	Frugivore	Tropical lowland evergreen forest	Resident
	White-collared Manakin	*Manacus candei*	Herbivore	Frugivore	Tropical lowland evergreen forest	Resident
	White-crowned Manakin	*Manacus candei*	Herbivore	Frugivore	Tropical lowland evergreen forest	Resident
Polioptilidae (Gnatcatchers)	Long-billed Gnatwren	*Ramphocaenus melanurus*	Carnivore	Invertivore	Tropical lowland evergreen forest	Resident
Spindalidae (Spindalises)	Puerto Rican Spindalis	*Spindalis portoricensis*	Herbivore	Frugivore	Secondary forest	Resident
Thamnophilidae (Typical Antbirds)	Band-tailed Antbird	*Hypocnemoides maculicauda*	Carnivore	Invertivore	Flooded tropical evergreen forest	Resident
	Barred Antshrike	*Thamnophilus doliatus*	Carnivore	Invertivore	Second-growth scrub	Resident
	Black-crowned Antshrike	*Thamnophilus atrinucha*	Carnivore	Invertivore	Tropical lowland evergreen forest	Resident
	Black-faced Antbird	*Myrmoborus myotherinus*	Carnivore	Invertivore	Tropical lowland evergreen forest	Resident
	Black-hooded Antshrike	*Thamnophilus bridgesi*	Carnivore	Invertivore	Tropical lowland evergreen forest	Resident
	Black-spotted Bare-eye	*Phlegopsis nigromaculata*	Carnivore	Invertivore	Tropical lowland evergreen forest	Resident
	Bluish-slate Antshrike	*Thamnomanes schistogynus*	Carnivore	Invertivore	Tropical lowland evergreen forest	Resident
	Checker-throated Stipplethroat	*Epinecrophylla fulviventris*	Carnivore	Invertivore	Tropical lowland evergreen forest	Resident
	Chestnut-tailed Antbird	*Sciaphylax hemimelaena*	Carnivore	Invertivore	Tropical lowland evergreen forest	Resident
	Common Scale-backed Antbird	*Willisornis poecilinotus*	Carnivore	Invertivore	Tropical lowland evergreen forest	Resident
	Dot-winged Antwren	*Microrhopias quixensis*	Carnivore	Invertivore	Tropical lowland evergreen forest	Resident
	Dusky Antbird	*Cercomacroides tyrannina*	Carnivore	Invertivore	Tropical lowland evergreen forest	Resident
	Dusky-throated	*Thamnomanes*	Carnivore	Invertivore	Tropical lowland	Resident

	Antshrike	*ardesiacus*			evergreen forest	
	Gray Antwren	*Myrmotherula menetriesii*	Carnivore	Invertivore	Tropical lowland evergreen forest	Resident
	Great Antshrike	*Taraba major*	Carnivore	Invertivore	Tropical lowland evergreen forest	Resident
	Ihering's Antwren	*Myrmotherula iheringi*	Carnivore	Invertivore	Tropical lowland evergreen forest	Resident
	Long-winged Antwren	*Myrmotherula longipennis*	Carnivore	Invertivore	Tropical lowland evergreen forest	Resident
	Plain-throated Antwren	*Isleria hauxwelli*	Carnivore	Invertivore	Tropical lowland evergreen forest	Resident
	Plain-winged Antshrike	*Thamnophilus schistaceus*	Carnivore	Invertivore	Tropical lowland evergreen forest	Resident
	Plumbeous Antbird	*Myrmelastes hyperythrus*	Carnivore	Invertivore	Flooded tropical evergreen forest	Resident
	Rufous-backed Stipplethroat	*Epinecrophylla haematonota*	Carnivore	Invertivore	Tropical lowland evergreen forest	Resident
	Slaty Antwren	*Myrmotherula schisticolor*	Carnivore	Invertivore	Montane evergreen forest	Resident
	Sooty Antbird	*Hafferia fortis*	Carnivore	Invertivore	Tropical lowland evergreen forest	Resident
	Spot-winged Antshrike	*Pygiptila stellaris*	Carnivore	Invertivore	Tropical lowland evergreen forest	Resident
	Streak-crowned Antvireo	*Dysithamnus striaticeps*	Carnivore	Invertivore	Tropical lowland evergreen forest	Resident
	White-browed Antbird	*Myrmoborus leucophrys*	Carnivore	Invertivore	Tropical lowland evergreen forest	Resident
	White-flanked Antwren	*Myrmotherula axillaris*	Carnivore	Invertivore	Tropical lowland evergreen forest	Resident
	White-fringed Antwren	*Formicivora grisea*	Carnivore	Invertivore	Tropical deciduous forest	Resident
	White-shouldered Antbird	*Akletos melanoceps*	Carnivore	Invertivore	Flooded tropical evergreen forest	Resident
	White-shouldered Antshrike	*Thamnophilus aethiops*	Carnivore	Invertivore	Tropical lowland evergreen forest	Resident
	White-throated Antbird	*Oneillornis salvini*	Carnivore	Invertivore	Tropical lowland evergreen forest	Resident
Thraupidae (Tanagers and Allies)	Bananaquit	*Coereba flaveola*	Herbivore	Nectarivore	Tropical lowland evergreen forest	Resident
	Black-billed Seed-Finch	*Sporophila atrirostris*	Herbivore	Granivore	Riparian thickets	Resident
	Black-headed Saltator	*Saltator atriceps*	Herbivore	Omnivore	Tropical lowland evergreen forest	Resident
	Blue-black Grassquit	*Volatinia jacarina*	Herbivore	Granivore	Second-growth scrub	Resident
	Blue-gray Tanager	*Thraupis episcopus*	Omnivore	Omnivore	Tropical lowland evergreen forest	Resident
	Buff-throated Saltator	*Saltator maximus*	Herbivore	Frugivore	Tropical lowland evergreen forest	Resident
	Chestnut-bellied Seed-Finch	*Sporophila angolensis*	Omnivore	Granivore	Second-growth scrub	Resident
	Chestnut-bellied Seedeater	*Sporophila castaneiventris*	Herbivore	Granivore	Second-growth scrub	Resident
	Crimson-collared Tanager	*Ramphocelus sanguinolentus*	Omnivore	Invertivore	Tropical lowland evergreen forest	Resident
	Golden-hooded Tanager	*Stilpnia larvata*	Omnivore	Frugivore	Tropical lowland evergreen forest	Resident
	Gray-headed Tanager	*Eucometis penicillata*	Carnivore	Invertivore	Tropical lowland evergreen forest	Resident

	Grayish Saltator	*Saltator coerulescens*	Herbivore	Omnivore	Second-growth scrub	Resident
	Green-and-gold Tanager	*Tangara schrankii*	Herbivore	Frugivore	Tropical lowland evergreen forest	Resident
	Morelet's Seedeater	*Sporophila morelleti*	Herbivore	Granivore	Second-growth scrub	Resident
	Puerto Rican Bullfinch	*Melopyrrha portoricensis*	Herbivore	Omnivore	Montane evergreen forest	Resident
	Red-legged Honeycreeper	*Cyanerpes cyaneus*	Omnivore	Omnivore	Tropical lowland evergreen forest	Resident
	Scarlet-rumped Tanager	*Ramphocelus passerinii*	Omnivore	Omnivore	Tropical lowland evergreen forest	Resident
	Silver-beaked Tanager	*Ramphocelus carbo*	Omnivore	Omnivore	Secondary forest	Resident
	Slate-colored Seedeater	*Sporophila schistacea*	Herbivore	Granivore	Tropical lowland evergreen forest	Resident
	Thick-billed Seed-Finch	*Sporophila funerea*	Herbivore	Granivore	Second-growth scrub	Resident
	Variable Seedeater	*Sporophila corvina*	Herbivore	Granivore	Second-growth scrub	Resident
	White-shouldered Tanager	*Loriotus luctuosus*	Carnivore	Invertivore	Tropical lowland evergreen forest	Resident
	White-winged Shrike-Tanager	*Lanio versicolor*	Carnivore	Invertivore	Tropical lowland evergreen forest	Resident
Tityridae (Tityras and Allies)	Cinereous Mourner	*Laniocera hypopyrra*	Carnivore	Invertivore	Tropical lowland evergreen forest	Resident
	Northern Schiffornis	*Schiffornis veraepacis*	Omnivore	Omnivore	Tropical lowland evergreen forest	Resident
	Pink-throated Becard	*Pachyramphus minor*	Omnivore	Omnivore	Tropical lowland evergreen forest	Resident
	Rose-throated Becard	*Pachyramphus aglaiae*	Omnivore	Omnivore	Gallery forest	Resident
	Varzea Schiffornis	*Schiffornis major*	Omnivore	Frugivore	Flooded tropical evergreen forest	Resident
Troglodytidae (Wrens)	Bicolored Wren	*Campylorhynchus griseus*	Carnivore	Invertivore	Arid lowland scrub	Resident
	Cabanis's Wren	*Cantorchilus modestus*	Carnivore	Invertivore	Tropical deciduous forest	Resident
	House Wren	*Troglodytes aedon*	Carnivore	Invertivore	Second-growth scrub	Resident
	Moustached Wren	*Pheugopedius genibarbis*	Carnivore	Invertivore	Tropical lowland evergreen forest	Resident
	Musician Wren	*Cyphorhinus arada*	Carnivore	Invertivore	Tropical lowland evergreen forest	Resident
	Riverside Wren	*Cantorchilus semibadius*	Carnivore	Invertivore	Tropical lowland evergreen forest	Resident
	Rufous-and-white Wren	*Thryophilus rufalbus*	Carnivore	Invertivore	Tropical lowland evergreen forest	Resident
	Rufous-naped Wren	*Campylorhynchus rufinucha*	Carnivore	Invertivore	Tropical deciduous forest	Resident
	Scaly-breasted Wren	*Microcerculus marginatus*	Carnivore	Invertivore	Tropical lowland evergreen forest	Resident
	Spot-breasted Wren	*Pheugopedius maculipectus*	Carnivore	Invertivore	Tropical lowland evergreen forest	Resident
	Stripe-breasted Wren	*Cantorchilus thoracicus*	Carnivore	Invertivore	Tropical lowland evergreen forest	Resident
	White-bellied Wren	*Uropsila leucogastra*	Carnivore	Invertivore	Tropical deciduous forest	Resident
	White-breasted Wood-Wren	*Henicorhina leucosticta*	Carnivore	Invertivore	Tropical lowland evergreen forest	Resident

Turdidae (Thrushes and Allies)	Black-billed Thrush	*Turdus ignobilis*	Omnivore	Omnivore	River-edge forest	Resident
	Black-headed Nightingale-Thrush	*Catharus mexicanus*	Omnivore	Invertivore	Montane evergreen forest	Resident
	Clay-colored Thrush	*Turdus grayi*	Omnivore	Omnivore	Tropical lowland evergreen forest	Resident
	Hauxwell's Thrush	*Turdus hauxwelli*	Omnivore	Invertivore	Flooded tropical evergreen forest	Resident
	Red-legged Thrush	*Turdus plumbeus*	Carnivore	Omnivore	Tropical deciduous forest	Resident
	Slate-colored Solitaire	*Myadestes unicolor*	Herbivore	Frugivore	Montane evergreen forest	Resident
	Swainson's Thrush	*Catharus ustulatus*	Omnivore	Invertivore	Montane evergreen forest	Full migrant
	Veery	*Catharus fuscescens*	Carnivore	Invertivore	Tropical lowland evergreen forest	Full migrant
	White-necked Thrush	*Turdus albicollis*	Omnivore	Invertivore	Tropical lowland evergreen forest	Resident
	White-throated Thrush	*Turdus assimilis*	Omnivore	Invertivore	Montane evergreen forest	Resident
	Wood Thrush	*Hylocichla mustelina*	Omnivore	Invertivore	Tropical lowland evergreen forest	Full migrant
Tyrannidae (Tyrant Flycatchers)	Acadian Flycatcher	*Empidonax virescens*	Carnivore	Invertivore	Tropical lowland evergreen forest	Full migrant
	Black Phoebe	*Sayornis nigricans*	Carnivore	Invertivore	Riparian thickets	Resident
	Bran-colored Flycatcher	*Myiophobus fasciatus*	Carnivore	Invertivore	Second-growth scrub	Resident
	Bright-rumped Attila	*Attila spadiceus*	Carnivore	Invertivore	Tropical lowland evergreen forest	Resident
	Brown-crested Flycatcher	*Myiarchus tyrannulus*	Carnivore	Invertivore	Tropical deciduous forest	Resident
	Common Tody-Flycatcher	*Todirostrum cinereum*	Carnivore	Invertivore	Tropical lowland evergreen forest	Resident
	Dusky-capped Flycatcher	*Myiarchus tuberculifer*	Carnivore	Invertivore	Montane evergreen forest	Resident
	Eastern Wood-Pewee	*Contopus virens*	Carnivore	Invertivore	Tropical lowland evergreen forest	Full migrant
	Eye-ringed Flatbill	*Rhynchocyclus brevirostris*	Omnivore	Invertivore	Tropical lowland evergreen forest	Resident
	Flammulated Pygmy-Tyrant	*Hemitriccus flammulatus*	Carnivore	Invertivore	Tropical lowland evergreen forest	Resident
	Golden-crowned Spadebill	*Platyrinchus coronatus*	Carnivore	Invertivore	Tropical lowland evergreen forest	Resident
	Gray Kingbird	*Tyrannus dominicensis*	Carnivore	Omnivore	Second-growth scrub	Partial migrant
	Great Kiskadee	*Pitangus sulphuratus*	Carnivore	Omnivore	Secondary forest	Resident
	Greenish Elaenia	*Myiopagis viridicata*	Omnivore	Invertivore	Tropical lowland evergreen forest	Resident
	Hispaniolan Pewee	*Contopus hispaniolensis*	Carnivore	Invertivore	Pine forest	Resident
	Lesser Elaenia	*Elaenia chiriquensis*	Omnivore	Omnivore	Cerrado	Resident
	Mountain Elaenia	*Elaenia frantzii*	Herbivore	Frugivore	Montane evergreen forest	Resident
	Northern Beardless-Tyrannulet	*Camptostoma imberbe*	Carnivore	Invertivore	Gallery forest	Resident
	Northern Bentbill	*Oncostoma cinereigulare*	Carnivore	Invertivore	Tropical lowland evergreen forest	Resident
	Ochre-bellied	*Mionectes*	Herbivore	Frugivore	Tropical lowland	Resident

		Flycatcher	*oleagineus*			evergreen forest	
		Olivaceous Flatbill	*Rhynchocyclus olivaceus*	Carnivore	Invertivore	Tropical lowland evergreen forest	Resident
		Pied Water-Tyrant	*Fluvicola pica*	Carnivore	Invertivore	Freshwater marshes	Resident
		Ringed Antpipit	*Corythopis torquatus*	Carnivore	Invertivore	Tropical lowland evergreen forest	Resident
		Rusty-margined Flycatcher	*Myiozetetes cayanensis*	Carnivore	Invertivore	Second-growth scrub	Resident
		Sepia-capped Flycatcher	*Leptopogon amaurocephalus*	Carnivore	Invertivore	Tropical lowland evergreen forest	Resident
		Slate-headed Tody-flycatcher	*Poecilotriccus sylvia*	Carnivore	Invertivore	Tropical lowland evergreen forest	Resident
		Slender-footed Tyrannulet	*Zimmerius gracilipes*	Carnivore	Invertivore	Tropical lowland evergreen forest	Resident
		Social Flycatcher	*Myiozetetes similis*	Carnivore	Invertivore	Tropical lowland evergreen forest	Resident
		Southern Mouse-colored Tyrannulet	*Nesotriccus murina*	Omnivore	Invertivore	Arid lowland scrub	Resident
		Stub-tailed Spadebill	*Platyrinchus cancrominus*	Carnivore	Invertivore	Tropical lowland evergreen forest	Resident
		Sulphur-bellied Flycatcher	*Myiodynastes luteiventris*	Omnivore	Omnivore	River-edge forest	Partial migrant
		Tropical Kingbird	*Tyrannus melancholicus*	Carnivore	Invertivore	Secondary forest	Resident
		Tropical Pewee	*Contopus cinereus*	Carnivore	Invertivore	Tropical lowland evergreen forest	Resident
		Western Wood-Pewee	*Contopus sordidulus*	Carnivore	Invertivore	Montane evergreen forest	Partial migrant
		White-bellied Tody-Tyrant	*Hemitriccus griseipectus*	Carnivore	Invertivore	Tropical lowland evergreen forest	Resident
		Wing-barred Piprites	*Piprites chloris*	Carnivore	Invertivore	Tropical lowland evergreen forest	Resident
		Yellow-bellied Elaenia	*Elaenia flavogaster*	Omnivore	Invertivore	Second-growth scrub	Resident
		Yellow-bellied Flycatcher	*Empidonax flaviventris*	Carnivore	Invertivore	Tropical lowland evergreen forest	Full migrant
		Yellow-crowned Tyrannulet	*Tyrannulus elatus*	Omnivore	Invertivore	River-edge forest	Resident
		Yellow-olive Flycatcher	*Tolmomyias sulphurescens*	Carnivore	Invertivore	Tropical lowland evergreen forest	Resident
	Vireonidae (Vireos, Shrike-Babblers, and Erpornis)	Black-whiskered Vireo	*Vireo altiloquus*	Omnivore	Invertivore	Tropical deciduous forest	Partial migrant
		Lesser Greenlet	*Pachysylvia decurtata*	Carnivore	Invertivore	Tropical lowland evergreen forest	Resident
		Mangrove Vireo	*Vireo pallens*	Carnivore	Invertivore	Tropical deciduous forest	Resident
		Red-eyed Vireo	*Vireo olivaceus*	Omnivore	Invertivore	Tropical lowland evergreen forest	Full migrant
		White-eyed Vireo	*Vireo griseus*	Carnivore	Invertivore	Tropical lowland evergreen forest	Partial migrant
		Yellow-green Vireo	*Vireo flavoviridis*	Omnivore	Invertivore	Tropical lowland evergreen forest	Partial migrant
		Yellow-winged Vireo	*Vireo carmioli*	Carnivore	Invertivore	Montane evergreen forest	Resident
Pelecaniformes	Ardeidae (Herons, Egrets, and Bitterns)	Cattle Egret	*Bubulcus ibis*	Carnivore	Omnivore	Pastures/agricultural lands	Resident
		Green Heron	*Butorides virescens*	Carnivore	Aquatic predator	Freshwater lakes and ponds	Resident

Piciformes	Capitonidae (New World Barbets)	Gilded Barbet	*Capito auratus*	Herbivore	Frugivore	Tropical lowland evergreen forest	Resident
	Picidae (Woodpeckers)	Golden-cheeked Woodpecker	*Melanerpes chrysogenys*	Carnivore	Invertivore	Tropical deciduous forest	Resident
		Golden-fronted Woodpecker	*Melanerpes aurifrons*	Omnivore	Omnivore	Arid lowland scrub	Resident
		Hispaniolan Woodpecker	*Melanerpes striatus*	Omnivore	Invertivore	Tropical lowland evergreen forest	Resident
		Hoffmann's Woodpecker	*Melanerpes hoffmannii*	Omnivore	Invertivore	Tropical deciduous forest	Resident
		Little Woodpecker	*Dryobates passerinus*	Carnivore	Invertivore	River-edge forest	Resident
		Rufous-breasted Piculet	*Picumnus rufiventris*	Carnivore	Invertivore	River-edge forest	Resident
		Smoky-brown Woodpecker	*Dryobates fumigatus*	Carnivore	Invertivore	Montane evergreen forest	Resident
		Spot-breasted Woodpecker	*Colaptes punctigula*	Carnivore	Invertivore	River-edge forest	Resident
		Yucatan Woodpecker	*Melanerpes pygmaeus*	Omnivore	Invertivore	Arid lowland scrub	Resident
	Ramphastidae (Toucans)	Curl-crested Aracari	*Pteroglossus beauharnaisii*	Herbivore	Frugivore	Tropical lowland evergreen forest	Resident
		Ivory-billed Aracari	*Pteroglossus azara*	Herbivore	Frugivore	Tropical lowland evergreen forest	Resident
		Lettered Aracari	*Pteroglossus inscriptus*	Herbivore	Frugivore	Flooded tropical evergreen forest	Resident
Psittaciformes	Psittacidae (New World and African Parrots)	Hispaniolan Parakeet	*Psittacara chloropterus*	Herbivore	Frugivore	Montane evergreen forest	Resident
Strigiformes	Strigidae (Owls)	Burrowing Owl	*Athene cunicularia*	Carnivore	Terrestrial vertivore	Northern temperate grassland	Resident
		Tawny-bellied Screech-Owl	*Megascops watsonii*	Carnivore	Invertivore	Tropical lowland evergreen forest	Resident
		Tropical Screech-Owl	*Megascops choliba*	Carnivore	Invertivore	Secondary forest	Resident
Suliformes	Sulidae (Boobies and Gannets)	Brown Booby	*Sula leucogaster*	Carnivore	Aquatic predator	Coastal waters	Resident
Tinamiformes	Tinamidae (Tinamous)	Little Tinamou	*Crypturellus soui*	Herbivore	Omnivore	Tropical lowland evergreen forest	Resident
Trogoniformes	Trogonidae (Trogons)	Black-tailed Trogon	*Trogon melanurus*	Herbivore	Frugivore	Tropical lowland evergreen forest	Resident

Table S4. Linear mixed-effects model selection results examining the influence of biotic and abiotic factors on natural log-transformed total mercury (THg) concentrations (µg/g) in Neotropical birds sampled across Central America, South America, and the West Indies from 2007–2023. A nested sampling site/station term, a nested family/species/individual term, and year are random effects in all models.

Model	ΔAIC_C	w_i	–logL	R^2	K
Functional trait model					
Tissue + Trophic niche + ASGM presence + Primary habitat + (1 | Site/Station) + (1 | Family/Species/Band #) + (1 | Year)	0.00	0.68	–2941.0	0.92	22
Tissue + Trophic niche + ASGM presence + Primary habitat + Migratory status + (1 | Site/Station) + (1 | Family/Species/Band #) + (1 | Year)	2.32	0.21	–2940.2	0.91	24

Tissue + Trophic niche + ASGM presence + (1 | Site/Station) + (1 | Family/Species/Band #) + (1 | Year)	4.05	0.09	–2948.2	0.92	17
Tissue + Trophic niche + ASGM presence + Migratory status + (1 | Site/Station) + (1 | Family/Species/Band #) + (1 | Year)	6.97	0.02	–2947.6	0.92	19
All other candidate models (n = 28)	> 14	< 0.01	—	≤ 0.89	—
Temporal model					
Season + Trophic niche + Season*Trophic niche + (1 | Site/Station) + (1 | Family/Species/Band #) + (1 | Year)	0.0	99.9	–1256.9	0.95	19
Season + Trophic niche + (1 | Site/Station) + (1 | Family/Species/Band #) + (1 | Year)	30.6	< 0.01	–1277.3	0.95	14
Season + (1 | Family/Species/Band #) + (1 | Year)	31.9	< 0.01	–1279.0	0.95	13
Season + (1 | Site/Station) + (1 | Family/Species/Band #) + (1 | Year)	76.5	< 0.01	–1305.4	0.95	9
(1 | Site/Station) + (1 | Family/Species/Band #) + (1 | Year)	78.2	< 0.01	–1307.3	0.95	8

Table S5. Back-transformed coefficient estimates, 95% confidence intervals, standard deviations, and *p*-values for biotic and abiotic factors affecting total mercury (THg) concentrations (µg/g) in Neotropical birds sampled across Central America, South America, and the West Indies from 2007–2023. Covariate effects highlighted in bold and non-overlapping letters indicate statistically significant differences ($p < 0.05$) among groups based on analysis of variance (ANOVA) and Tukey pairwise comparisons.

	Model summary			ANOVA	
**Covariate - Tukey pairwise comparisons**	**Estimate (95% CI)**	***p*-value**	**SD (95% CI)**	χ^2	***p*-value**
Top-performing functional trait model					
Intercept	0.13 (0.06, 0.26)	< 0.001			
**Fixed effects**					
**Tissue type (reference: Whole blood - b)**				**3358.75**	**< 0.001**
**Body feather - a**	**18.56 (16.30, 21.12)**	**< 0.001**			
**Tail feather - a**	**18.08 (16.22, 20.16)**	**< 0.001**			
**Trophic niche (reference: Aquatic predator - ab)**				**85.59**	**< 0.001**
Terrestrial vertivore - a	1.88 (0.48, 7.44)	0.349			
Invertivore - a	0.58 (0.19, 1.77)	0.349			
Nectarivore - abc	0.33 (0.08, 1.39)	0.134			
**Omnivore - b**	**0.29 (0.09, 0.88)**	**0.029**			
**Frugivore - c**	**0.12 (0.04, 0.39)**	**< 0.001**			
**Granivore - c**	**0.08 (0.02, 0.28)**	**< 0.001**			
**Primary habitat association (reference: Aquatic - a)**				**14.47**	**0.013**
Lowland deciduous forest - ab	0.41 (0.15, 1.10)	0.08			
**Grassland/scrub - ab**	**0.31 (0.12, 0.78)**	**0.013**			
**Lowland evergreen forest - ab**	**0.30 (0.12, 0.75)**	**0.009**			
**Secondary forest - b**	**0.24 (0.09, 0.62)**	**0.003**			
**Montane evergreen forest - b**	**0.19 (0.07, 0.54)**	**0.002**			
**ASGM presence (reference: ASGM absent - b)**				**20.54**	**< 0.001**
**ASGM present - a**	**4.30 (2.29, 8.09)**	**< 0.001**			
**Random effects**					

**Sampling site**			**1.77 (1.52, 2.19)**	**321.52**	**< 0.001**
**Sampling station**			**1.27 (1.16, 1.47)**		
**Family**			**1.55 (1.34, 1.91)**	**639.34**	**< 0.001**
**Species**			**2.19 (2.01, 2.41)**		
**Band number**			**1.29 (1.20, 1.42)**		
**Year**			**1.21 (1.11, 1.41)**	**103.67**	**< 0.001**
Top-performing temporal model					
Intercept	0.13 (0.06, 0.29)	< 0.001			
**Fixed effects**					
Season (reference: Dry season)				3.65	0.056
**Wet season**	**2.32 (1.39, 3.84)**	**0.001**			
**Trophic niche (reference: Aquatic predator)**				**92.86**	**< 0.001**
**Invertivore**	**0.26 (0.12, 0.58)**	**< 0.001**			
**Omnivore**	**0.12 (0.05, 0.30)**	**< 0.001**			
**Nectarivore**	**0.03 (0.00, 0.26)**	**0.001**			
**Frugivore**	**0.03 (0.01, 0.09)**	**< 0.001**			
**Granivore**	**0.03 (0.01, 0.08)**	**< 0.001**			
**Season*Trophic niche (reference: Dry season - Aquatic predator)**				**41.19**	**< 0.001**
Wet season - Nectarivore	1.92 (0.63, 5.90)	0.248			
**Wet season - Omnivore**	**0.37 (0.19, 0.71)**	**0.003**			
**Wet season - Invertivore**	**0.33 (0.19, 0.53)**	**< 0.001**			
**Wet season - Frugivore**	**0.23 (0.12, 0.40)**	**< 0.001**			
**Wet season - Granivore**	**0.15 (0.06, 0.39)**	**< 0.001**			
**Random effects**					
**Sampling site**			**2.22 (1.69, 3.35)**	**641.02**	**< 0.001**
**Sampling station**			**1.58 (1.29, 2.30)**		
**Family**			**1.43 (1.18, 2.19)**	**407.83**	**< 0.001**
**Species**			**2.59 (2.26, 3.03)**		
**Band number**			**1.79 (1.57, 2.14)**		
**Year**			**1.24 (1.09, 1.69)**	**489.96**	**< 0.001**

Table S6. Total mercury (THg) concentrations (µg/g) among Neotropical bird trophic niches sampled across Central America, South America, and the West Indies from 2007–2023. Countries sampled, sampling duration, number of species sampled, sample size (*n*), arithmetic mean ± standard deviation (SD), range, and coefficient of variation (CV) are summarized by trophic niche and tissue type. A dash (–) indicates there are no data to report.

Trophic niche	Sampled tissue	Countries sampled	Sampling duration	Species sampled	*n*	Arithmetic mean ± SD	Range	CV
Aquatic predator	Whole blood	BZ, CO, CR, DR, MX, NI	2007–2023	11	60	0.431 ± 0.511	0.017–3.112	118.6%
	Body feather	BZ, CO, PE	2019–2023	4	22	23.958 ± 20.083	1.718–72.795	83.8%
	Tail feather	BZ, CR, MX, NI, PA	2007–2021	7	54	4.432 ± 5.812	0.102–25.841	131.1%
Frugivore	Whole blood	BZ, CR, DR, PR	2007–2021	9	84	0.015 ± 0.032	0.001–0.278	218.0%
	Body feather	BZ, PE	2019–2021	19	115	0.435 ± 0.344	0.010–1.24	79.2%

	Tail feather	BZ, CR, NI, PE, PR	2007–2021	15	120	0.322 ± 0.364	0.008–2.755	113.2%
Granivore	Whole blood	BZ, CO, DR, MX, NI	2008–2023	12	42	0.008 ± 0.014	0.001–0.091	179.3%
	Body feather	BZ, CO, PE	2019–2023	13	42	0.236 ± 0.359	0.001–1.462	152.0%
	Tail feather	BZ, CR, MX, NI, PE	2007–2021	9	44	0.076 ± 0.135	0.001–0.616	178.4%
Invertivore	Whole blood	BZ, CO, CR, DR, MX, NI, PR	2007–2023	105	650	0.146 ± 0.251	0.001–3.195	171.3%
	Body feather	BZ, CO, DR, PE	2018–2023	105	273	2.532 ± 3.587	0.008–28.494	141.7%
	Tail feather	BZ, CR, DR, NI, PA, PE, PR	2007–2021	112	310	1.430 ± 1.458	0.001–8.606	102.0%
Nectarivore	Whole blood	DR, PR	2010–2017	1	26	0.007 ± 0.007	0.001–0.032	108.5%
	Body feather	PE	2019	6	11	1.714 ± 1.004	0.072–3.490	58.6%
	Tail feather	PE, PR	2010–2019	6	38	0.544 ± 0.555	0.037–2.276	102.0%
Omnivore	Whole blood	BZ, CO, CR, DR, MX, NI, PR	2007–2023	22	101	0.049 ± 0.146	0.001–1.414	299.4%
	Body feather	BZ, CO, PE	2010–2023	22	93	1.658 ± 3.666	0.013–20.227	221.1%
	Tail feather	BZ, CR, MX, NI, PE, PR	2007–2021	28	121	0.464 ± 0.774	0.001–7.585	166.9%
Terrestrial vertivore	Body feather	PE	2009–2019	14	106	3.380 ± 3.282	0.380–19.887	97.1%

Table S7. Total mercury (THg) concentrations (μg/g) among Neotropical bird primary habitat associations sampled across Central America, South America, and the West Indies from 2007–2023. Countries sampled, sampling duration, number of species sampled, sample size (*n*), arithmetic mean ± standard deviation (SD), range, and coefficient of variation (CV) are summarized by habitat and tissue type. A dash (–) indicates there are no data to report.

Primary habitat	Sampled tissue	Countries sampled	Sampling duration	Species sampled	*n*	Arithmetic mean ± SD	Range	CV
Aquatic	Whole blood	BZ, CO, CR, DR, MX, NI	2007–2023	15	65	0.457 ± 0.514	0.017–3.112	112.5%
	Body feather	BZ, CO, PE	2019–2023	8	30	19.993 ± 18.761	1.220–72.795	93.8%
	Tail feather	BZ, CR, MX, NI, PA	2007–2021	7	54	4.432 ± 5.812	0.102–25.841	131.1%
Grassland/scrub	Whole blood	BZ, CO, CR, DR, MX, NI, PR	2007–2023	28	113	0.064 ± 0.104	0.001–0.699	163.3%
	Body feather	BZ, CO, DR, PE	2011–2023	26	72	2.175 ± 5.040	0.001–28.494	231.8%
	Tail feather	BZ, CR, DR, MX, NI, PA, PE, PR	2010–2021	18	66	0.589 ± 1.173	0.001–5.165	199.2%
Lowland deciduous forest	Whole blood	BZ, CO, DR, MX, NI, PR	2007–2023	17	44	0.119 ± 0.155	0.001–0.791	130.2%
	Body feather	BZ, CO, PE	2009–2023	10	19	2.949 ± 4.826	0.004–17.802	163.7%

	Tail feather	BZ, CR, NI, PR	2007–2021	13	29	0.566 ± 0.705	0.001–3.257	124.6%
Lowland evergreen forest	Whole blood	BZ, CO, CR, DR, MX, NI, PR	2007–2023	75	634	0.128 ± 0.249	0.001–3.195	193.9%
	Body feather	BZ, CO, PE	2009–2023	116	469	1.861 ± 2.498	0.006–19.887	134.2%
	Tail feather	BZ, CR, NI, PA, PE, PR	2007–2021	111	451	1.005 ± 1.317	0.001–8.606	131.0%
Montane evergreen forest	Whole blood	BZ, CR, NI, PR	2007–2021	8	26	0.032 ± 0.059	0.001–0.259	187.4%
	Body feather	BZ, PE	2019–2021	4	7	0.630 ± 0.445	0.008–1.101	70.6%
	Tail feather	BZ, CR, NI, PE, PR	2007–2021	16	43	0.465 ± 0.516	0.026–1.869	110.9%
Secondary forest	Whole blood	BZ, CO, CR, DR, MX, NI, PR	2007–2023	16	80	0.043 ± 0.050	0.001–0.306	117.1%
	Body feather	BZ, CO, PE	2010–2023	17	63	1.882 ± 3.025	0.038–19.466	160.8%
	Tail feather	BZ, CR, MX, NI, PA, PE, PR	2007–2021	10	41	0.695 ± 0.676	0.031–3.275	97.3%

Table S8. Total mercury (THg) concentrations (µg/g) among all 17 orders, 51 families, and 322 Neotropical resident and migratory bird species sampled across Central America, South America, and the West Indies from 2007–2023. Countries sampled, sampling duration, sample size (*n*), arithmetic mean ± standard deviation (SD), range, and coefficient of variation (CV) are summarized by order, family, species and tissue type. Species are arranged alphabetically by order and family. A dash (–) indicates there are no data to report.

Order	Family	Common name	Latin name	Sampled tissue	Countries sampled	Sampling duration	*n*	Arithmetic mean ± SD	Range	CV
All orders	—	—	—	Whole blood	BZ, CO, CR, DR, MX, NI, PR	2007–2023	963	0.133 ± 0.264	0.001–3.195	198.8%
				Body feather	BZ, CO, DR, PE	2009–2023	663	2.730 ± 6.185	0.001–72.795	226.5%
				Tail feather	BZ, CR, DR, MX, NI, PA, PE, PR	2007–2021	690	1.166 ± 2.208	0.001–25.841	189.3%
Accipitriformes	All families	—	—	Body feather	PE	2009–2019	71	2.407 ± 2.106	0.380–8.057	87.5%
	Accipitridae (Hawks, Eagles, and Kites)	All species	—	Body feather	PE	2009–2019	71	2.407 ± 2.106	0.380–8.057	87.5%
		Bicolored Hawk	*Accipiter bicolor*	Body feather	PE	2009–2010	2	7.621 ± 0.295	7.412–7.829	3.87%
		Black-faced Hawk	*Leucopternis melanops*	Body feather	PE	2011	1	6.019	—	—
		Great Black Hawk	*Buteogallus urubitinga*	Body feather	PE	2010–2011	2	2.862 ± 0.599	2.438–3.285	20.9%
		Ornate Hawk-Eagle	*Spizaetus ornatus*	Body feather	PE	2010	1	0.989	—	—
		Roadside Hawk	*Rupornis magnirostris*	Body feather	PE	2009–2019	44	1.36 ± 0.696	0.38–3.208	51.2%

		Slate-colored Hawk	*Buteogallus schistaceus*	Body feather	PE	2009–2011	16	3.153 ± 2.039	1.378–7.675	64.7%
		White-browed Hawk	*Leucopternis kuhli*	Body feather	PE	2009–2011	5	6.535 ± 1.922	3.251–8.057	29.4%
Anseriformes	All families	—	—	Whole blood	DR	2017	1	0.081	—	—
	Anatidae (Ducks, Geese, and Waterfowl)	All species	—	Whole blood	DR	2017	1	0.081	—	—
		West Indian Whistling-Duck	*Dendrocygna arborea*	Whole blood	DR	2017	1	0.081	—	—
Caprimulgiformes	All families	—	—	Whole blood	BZ, CO	2021–2023	4	0.186 ± 0.168	0.036–0.428	90.4%
				Body feather	BZ, CO, DR, PE	2018–2023	23	1.530 ± 1.020	0.072–3.490	66.7
				Tail feather	BZ, DR, PE	2018–2021	26	0.875 ± 0.504	0.206–2.276	57.61%
	Apodidae (Swifts)	All species	—	Body feather	DR	2018	5	0.801 ± 0.609	0.359–1.653	76.1%
				Tail feather	DR	2018	5	0.717 ± 0.495	0.295–1.337	69.0%
		Antillean Palm-Swift	*Tachornis phoenicobia*	Body feather	DR	2018	5	0.801 ± 0.609	0.359–1.653	76.1%
				Tail feather	DR	2018	5	0.717 ± 0.495	0.295–1.337	69.0%
	Caprimulgidae (Nightjars and Allies)	All species	—	Whole blood	BZ, CO	2021–2023	4	0.186 ± 0.168	0.036–0.428	90.4%
				Body feather	BZ, CO, PE	2019–2023	7	1.76 ± 1.15	0.452–3.353	65.4
				Tail feather	BZ, PE	2019–2021	2	0.475 ± 0.381	0.206–0.744	80.1%
		Common Pauraque	*Nyctidromus albicollis*	Whole blood	BZ, CO	2021–2023	4	0.186 ± 0.168	0.036–0.428	90.4%
				Body feather	BZ, CO, PE	2019–2023	6	1.929 ± 1.161	0.452–3.353	60.2%
				Tail feather	BZ, PE	2019–2021	2	0.475 ± 0.381	0.206–0.744	80.1%
		Ocellated Poorwill	*Nyctiphrynus ocellatus*	Body feather	PE	2019	1	0.746	—	—
	Trochilidae (Hummingbirds)	All species	—	Body feather	PE	2019	11	1.714 ± 1.004	0.072–3.49	58.6%
				Tail feather	PE	2018–2019	19	0.959 ± 0.508	0.314–2.276	53.0%
		Blue-tailed Emerald	*Chlorostilbon mellisugus*	Tail feather	PE	2018	1	0.314	—	—
		Fork-tailed Woodnymph	*Thalurania furcata*	Body feather	PE	2019	2	1.461 ± 0.227	1.301–1.621	15.5%
				Tail feather	PE	2018–2019	3	1.231 ± 0.764	0.659–2.099	62.1%
		Great-billed Hermit	*Phaethornis malaris*	Tail feather	PE	2018	1	2.276	—	—
		Pale-tailed Barbthroat	*Threnetes leucurus*	Body feather	PE	2019	1	1.492	—	—
				Tail feather	PE	2018	3	0.760 ± 0.276	0.543–1.071	36.4%
		Reddish Hermit	*Phaethornis ruber*	Body feather	PE	2019	1	2.378	—	—
		Rufous-breasted Hermit	*Glaucis hirsutus*	Body feather	PE	2019	4	2.085 ± 0.976	1.320–3.49	46.8%
		Sapphire-spangled Emerald	*Chionomesa lactea*	Body feather	PE	2019	2	0.309 ± 0.335	0.072–0.546	108.3%

		White-bearded Hermit	*Phaethornis hispidus*	Body feather	PE	2019	1	3.108	—	—
				Tail feather	PE	2018–2019	11	0.878 ± 0.277	0.406–1.264	31.6%
Charadriiformes	All families	—	—	Whole blood	BZ, CO, DR, MX, NI	2012–2023	16	0.350 ± 0.370	0.017–1.414	105.6%
				Body feather	CO, PE	2019–2023	3	18.271 ± 2.321	15.706–20.227	12.7%
				Tail feather	NI	2012–2014	16	0.919 ± 0.673	0.102–2.407	73.2%
	Jacanidae (Jacanas)	All species	—	Whole blood	CO, MX, NI	2012–2013	6	0.427 ± 0.514	0.017–1.414	120.4%
				Body feather	PE	2019–2023	3	18.271 ± 2.321	15.706–20.227	12.7%
				Tail feather	NI	2012–2014	16	0.919 ± 0.673	0.102–2.407	73.2%
		Northern Jacana	*Jacana spinosa*	Whole blood	MX, NI	2012–2013	5	0.230 ± 0.196	0.017–0.417	85.4%
				Tail feather	NI	2012–2014	16	0.919 ± 0.673	0.102–2.407	73.2%
		Wattled Jacana	*Jacana jacana*	Whole blood	CO	2023	1	1.414	—	—
				Body feather	CO, PE	2019–2023	3	18.271 ± 2.321	15.706–20.227	12.7%
	Laridae (Gulls, Terns, and Skimmers)	All species	—	Whole blood	DR	2017	1	0.260	—	—
		Laughing Gull	*Leucophaeus atricilla*	Whole blood	DR	2017	1	0.260	—	—
	Scolopacidae (Sandpipers and Allies)	All species	—	Whole blood	BZ, DR, NI	2014–2021	9	0.309 ± 0.290	0.018–0.741	93.754%
		Least Sandpiper	*Calidris minutilla*	Whole blood	NI	2014	1	0.018	—	—
		Solitary Sandpiper	*Tringa solitaria*	Whole blood	DR, NI	2014–2017	2	0.442 ± 0.265	0.254–0.629	60.1%
		Spotted Sandpiper	*Actitis macularius*	Whole blood	BZ, DR, NI	2014–2021	6	0.314 ± 0.31	0.079–0.741	99.0%
Columbiformes	All families	—	—	Whole blood	BZ, CO, DR, MX, PR	2009–2023	16	0.002 ± 0.002	0.001–0.008	93.2%
				Body feather	BZ, CO PE	2019–2023	23	0.132 ± 0.162	0.004–0.753	122.8%
				Tail feather	BZ, CR, MX, NI, PE	2010–2021	16	0.073 ± 0.091	0.001–0.334	125.0%
	Columbidae (Pigeons and Doves)	All species	—	Whole blood	BZ, CO, DR, MX, PR	2009–2023	16	0.002 ± 0.002	0.001–0.008	93.2%
				Body feather	BZ, CO PE	2019–2023	23	0.132 ± 0.162	0.004–0.753	122.8%
				Tail feather	BZ, CR, MX, NI, PE	2010–2021	16	0.073 ± 0.091	0.001–0.334	125.0%
		Common Ground Dove	*Columbina passerina*	Tail feather	NI	2014	2	0.014 ± 0.001	0.013–0.014	5.2%
		Gray-fronted Dove	*Leptotila rufaxilla*	Body feather	PE	2019	3	0.058 ± 0.024	0.038–0.084	41.2%
		Gray-headed Dove	*Leptotila plumbeiceps*	Body feather	BZ	2019–2021	3	0.034 ± 0.024	0.006–0.048	71.5%
				Tail feather	BZ	2021	1	0.014	—	—
		Inca Dove	*Columbina inca*	Whole blood	MX	2013	1	0.008	—	—
				Tail feather	MX	2013	2	0.048 ± 0.033	0.025–0.072	68.5%

		Mourning Dove	*Zenaida macroura*	Whole blood	DR	2017	2	0.002 ± 0.001	0.001–0.003	70.7%
		Rock Pigeon	*Columba livia*	Whole blood	DR	2017	4	0.002 ± 0.002	0.001–0.005	100.0%
		Ruddy Ground Dove	*Columbina talpacoti*	Whole blood	BZ, CO	2021–2023	4	0.001 ± 0.000	0.001–0.001	17.7%
				Body feather	CO, PE	2019–2023	7	0.122 ± 0.043	0.027–0.15	35.3%
				Tail feather	NI	2014	2	0.019 ± 0.014	0.009–0.029	74.4%
		Ruddy Quail-Dove	*Geotrygon montana*	Whole blood	PR	2010	2	0.003 ± 0.002	0.002–0.004	46.4%
				Body feather	PE	2019	6	0.306 ± 0.236	0.111–0.753	77.3%
				Tail feather	CR	2010–2018	4	0.187 ± 0.099	0.117–0.334	53.0%
		White-tipped Dove	*Leptotila verreauxi*	Whole blood	BZ	2009–2021	3	0.003 ± 0.003	0.001–0.007	114.2%
				Body feather	BZ	2019–2021	4	0.017 ± 0.012	0.004–0.031	74.2%
				Tail feather	BZ, CR	2010–2021	5	0.048 ± 0.071	0.001–0.173	148.6%
Coraciiformes	All families	—	—	Whole blood	BZ, CO, CR, DR, NI	2007–2023	40	0.518 ± 0.591	0.020–3.112	114.0%
				Body feather	BZ, CO, PE	2019–2023	30	17.970 ± 19.859	0.227–72.795	110.5%
				Tail feather	BZ, CR, NI, PA, PE	2007–2021	41	5.607 ± 6.305	0.147–25.841	112.4%
	Alcedinidae (Kingfishers)	All species	—	Whole blood	BZ, CO, CR, NI	2007–2023	38	0.542 ± 0.597	0.020–3.112	110.1%
				Body feather	BZ, CO, PE	2019–2023	22	23.958 ± 20.083	1.718–72.795	83.8%
				Tail feather	BZ, CR, NI, PA	2007–2021	36	6.128 ± 6.478	1.299–25.841	105.7%
		Amazon Kingfisher	*Chloroceryle amazona*	Whole blood	CO, NI	2014–2023	3	1.789 ± 1.307	0.499–3.112	73.1%
				Body feather	CO, PE	2019–2023	4	23.902 ± 5.854	17.7–30.681	24.5%
				Tail feather	NI	2014	1	12.070	—	—
		American Pygmy Kingfisher	*Chloroceryle aenea*	Whole blood	BZ, CR	2007–2021	23	0.542 ± 0.403	0.069–1.37	74.2%
				Body feather	BZ, PE	2019–2021	9	12.038 ± 6.563	1.718–19.596	54.5%
				Tail feather	BZ, CR, PA	2007–2021	15	10.314 ± 7.734	1.475–25.841	74.9%
		Green Kingfisher	*Chloroceryle americana*	Whole blood	BZ, CR, NI	2008–2021	10	0.245 ± 0.200	0.02–0.515	81.7%
				Body feather	BZ, PE	2019–2021	8	35.754 ± 27.925	6.216–72.795	78.1%
				Tail feather	BZ, CR, NI	2011–2021	15	2.682 ± 2.601	1.299–11.546	96.9%
		Green-and-rufous Kingfisher	*Chloroceryle inda*	Body feather	PE	2019	1	37.079	—	—
				Tail feather	PA	2011	1	1.730	—	—
		Ringed Kingfisher	*Megaceryle torquata*	Whole blood	NI	2014	2	0.156 ± 0.047	0.122–0.189	30.5%
				Tail feather	NI	2014	4	2.968 ± 0.587	2.113–3.446	19.8%
	Momotidae (Motmots)	All species	—	Whole blood	BZ	2021	1	0.036	—	—
				Body feather	BZ, PE	2019–2021	8	1.504 ± 1.12	0.227–3.656	74.5%

				Tail feather	CR, PE	2010–2021	5	1.856 ± 3.221	0.147–7.585	173.5%
		Amazonian Motmot	*Momotus momota*	Body feather	PE	2019	7	1.686 ± 1.074	0.602–3.656	63.7%
				Tail feather	PE	2018	2	0.626 ± 0.535	0.248–1.005	85.4%
		Lesson's Motmot	*Momotus lessonii*	Whole blood	BZ	2021	1	0.036	—	—
				Body feather	BZ	2021	1	0.227	—	—
				Tail feather	BZ, CR	2010	2	0.221 ± 0.105	0.147–0.296	47.5%
		Rufous Motmot	*Baryphthengus martii*	Tail feather	PE	2018	1	7.585	—	—
	Todidae (Todies)	All species	—	Whole blood	DR	2017	1	0.097	—	—
		Broad-billed Tody	*Todus subulatus*	Whole blood	DR	2017	1	0.097	—	—
Cuculiformes	All families	—	—	Whole blood	DR, NI	2012–2017	6	0.139 ± 0.102	0.007–0.269	73.4%
				Body feather	BZ, PE	2019–2021	10	0.913 ± 0.315	0.445–1.603	34.5%
	Cuculidae (Cuckoos)	All species	—	Whole blood	DR, NI	2012–2017	6	0.139 ± 0.102	0.007–0.269	73.4%
				Body feather	BZ, PE	2019–2021	10	0.913 ± 0.315	0.445–1.603	34.5%
		Groove-billed Ani	*Crotophaga sulcirostris*	Whole blood	NI	2012	2	0.034 ± 0.037	0.007–0.06	111.9%
				Body feather	BZ	2021	1	0.445	—	—
		Hispaniolan Lizard-Cuckoo	*Coccyzus longirostris*	Whole blood	DR	2017	4	0.192 ± 0.076	0.093–0.269	39.5%
		Little Cuckoo	*Coccycua minuta*	Body feather	PE	2019	1	0.829	—	—
		Smooth-billed Ani	*Crotophaga ani*	Body feather	PE	2019	8	0.982 ± 0.3	0.675–1.603	30.6%
Falconiformes	All families	—	—	Body feather	PE	2009–2019	36	5.153 ± 4.333	0.374–19.887	84.1%
	Falconidae (Falcons and Caracaras)	All species	—	Body feather	PE	2009–2019	36	5.153 ± 4.333	0.374–19.887	84.1%
		Barred Forest-Falcon	*Micrastur ruficollis*	Body feather	PE	2009–2019	11	5.098 ± 3.39	1.797–11.744	66.5%
		Bat Falcon	*Falco rufigularis*	Body feather	PE	2019	1	9.12	—	—
		Black Caracara	*Daptrius ater*	Body feather	PE	2010–2011	2	0.444 ± 0.099	0.374–0.515	22.4%
		Buckley's Forest-Falcon	*Micrastur buckleyi*	Body feather	PE	2010–2011	2	1.676 ± 0.208	1.528–1.823	12.4%
		Collared Forest-Falcon	*Micrastur semitorquatus*	Body feather	PE	2009–2010	4	1.928 ± 0.735	0.970–2.736	38.1%
		Lined Forest-Falcon	*Micrastur gilvicollis*	Body feather	PE	2009–2011	12	6.46 ± 5.219	1.139–19.887	80.8%
		Slaty-backed Forest-Falcon	*Micrastur mirandollei*	Body feather	PE	2009–2011	4	7.708 ± 4.779	1.900–13.593	62.0%
Galbuliformes	All families	—	—	Whole blood	BZ	2021	1	0.063	—	—
				Body feather	PE	201–2021	11	2.691 ± 2.384	0.154–5.776	88.6%
				Tail feather	BZ, CR, PE	2010–2021	5	0.903 ± 0.832	0.150–2.237	92.1%

	Bucconidae (Puffbirds)	All species	—	Body feather	PE	2019	3	0.353 ± 0.316	0.153–0.717	89.5%
				Tail feather	CR, PE	2010–2018	3	0.512 ± 0.503	0.150–1.086	98.3%
		Black-fronted Nunbird	*Monasa nigrifrons*	Body feather	PE	2019	2	0.171 ± 0.024	0.153–0.188	14.2%
				Tail feather	PE	2018	1	0.150	—	—
		Chestnut-capp ed Puffbird	*Bucco macrodactylus*	Body feather	PE	2019	1	0.717	—	—
		Semicollared Puffbird	*Malacoptila semicincta*	Tail feather	PE	2018	1	1.086	—	—
		White-whiske red Puffbird	*Malacoptila panamensis*	Tail feather	CR	2010	1	0.299	—	—
	Galbulidae (Jacamars)	All species	—	Whole blood	BZ	2021	1	0.063	—	—
				Body feather	BZ, PE	2019–2021	8	3.568 ± 2.206	0.933–5.776	61.8%
				Tail feather	BZ, PE	2018–2021	2	1.490 ± 1.056	0.744–2.237	70.8%
		Bluish-fronted Jacamar	*Galbula cyanescens*	Body feather	PE	2019	7	3.848 ± 2.223	0.933–5.776	57.8%
				Tail feather	PE	2018	1	2.237	—	—
		Rufous-tailed Jacamar	*Galbula ruficauda*	Whole blood	BZ	2021	1	0.063	—	—
				Body feather	BZ	2021	1	1.604	—	—
				Tail feather	BZ	2021	1	0.744	—	—
Passeriformes	All families	—	—	Whole blood	BZ, CO, CR, DR, MX, NI, PR	2007–2023	862	0.114 ± 0.225	0.001–3.195	197.3%
				Body feather	BZ,CO, PE	2018–2023	424	1.792 ± 3.112	0.001–28.494	173.7%
				Tail feather	BZ, CR, MX, NI, PA, PE, PR	2007–2021	575	0.910 ± 1.233	0.001–8.606	135.4%
	Cardinalidae (Cardinals and Allies)	All species	—	Whole blood	BZ, DR, NI	2008–2021	16	0.053 ± 0.083	0.001–0.337	156.4%
				Body feather	BZ, PE	2019–2021	7	0.660 ± 0.602	0.198–1.602	91.3%
				Tail feather	BZ, NI, PE	2007–2021	14	0.457 ± 0.371	0.040–1.360	81.2%
		Blue Bunting	*Cyanocompsa parellina*	Whole blood	BZ	2008–2021	2	0.026 ± 0.027	0.007–0.045	101.9%
				Body feather	BZ	2021	1	0.198	—	—
				Tail feather	BZ, NI	2010–2021	2	0.040	0.040–0.125	72.9%
		Blue-black Grosbeak	*Cyanoloxia cyanoides*	Tail feather	BZ, PE	2007–2018	3	0.500 ± 0.146	0.336–0.616	29.2%
		Gray-throated Chat	*Granatellus sallaei*	Whole blood	BZ	2021	1	0.14	—	—
				Body feather	BZ	2021	1	1.602	—	—
				Tail feather	BZ	2007–2021	2	0.849 ± 0.209	0.702–0.997	24.615
		Hepatic Tanager	*Piranga flava*	Body feather	PE	2019	1	0.238	—	—
		Painted Bunting	*Passerina ciris*	Whole blood	NI	2012	1	0.003	—	—
		Red-crowned Ant-Tanager	*Habia rubica*	Body feather	PE	2019	2	0.326 ± 0.052	0.289–0.363	16.0%

			Tail feather	PE	2018–2019	3	0.354 ± 0.164	0.165–0.457	46.3%
	Red-throated Ant-Tanager	*Habia fuscicauda*	Whole blood	BZ	2008–2021	11	0.059 ± 0.093	0.017–0.337	158.1%
			Body feather	BZ	2021	2	0.964 ± 0.698	0.471–1.458	72.3%
			Tail feather	BZ, NI	2010–2021	4	0.491 ± 0.581	0.135–1.36	118.3%
Conopophagidae (Gnateaters)	All species	—	Body feather	PE	2019	1	1.756	—	—
			Tail feather	PE	2018	3	1.831 ± 1.120	0.968–3.097	61.2%
	Ash-thoated Gnateater	*Conopophaga peruviana*	Body feather	PE	2019	1	1.756	—	—
			Tail feather	PE	2018	3	1.831 ± 1.120	0.968–3.097	61.2%
Corvidae (Crows, Jays, and Magpies)	All species	—	Whole blood	BZ, NI	2009–2014	2	0.020 ± 0.011	0.012–0.027	55.4%
			Body feather	PE	2019	1	0.844	—	—
			Tail feather	NI	2014	1	0.165	—	—
	Brown Jay	*Psilorhinus morio*	Whole blood	BZ	2009	1	0.027	—	—
	Purplish Jay	*Cyanocorax cyanomelas*	Body feather	PE	2019	1	0.844	—	—
	White-throated Magpie-Jay	*Calocitta formosa*	Whole blood	NI	2014	1	0.012	—	—
			Tail feather	NI	2014	1	0.165	—	—
Dulidae (Palmchat)	All species	—	Whole blood	DR	2017	3	0.001 ± 0.000	0.001–0.001	0.0%
	Palmchat	*Dulus dominicus*	Whole blood	DR	2017	3	0.001 ± 0.000	0.001–0.001	0.0%
Formicariidae (Antthrushes)	All species	—	Body feather	PE	2019	7	1.679 ± 1.376	0.558–4.078	81.9%
			Tail feather	PE	2018	1	5.999	—	—
	Black-faced Antthrush	*Formicarius analis*	Body feather	PE	2019	6	1.453 ± 1.357	0.558–4.078	93.4%
			Tail feather	PE	2018	1	5.999	—	—
	Rufous-fronted Antthrush	*Formicarius rufifrons*	Body feather	PE	2019	1	3.040	—	—
Fringillidae (Finches, Euphonias, and Allies)	All species	—	Whole blood	BZ	2008–2021	7	0.003 ± 0.003	0.001–0.008	109.6%
			Body feather	BZ, PE	2019–2021	8	0.174 ± 0.376	0.010–1.101	215.3%
			Tail feather	BZ, CR, NI	2010–2021	12	0.071 ± 0.052	0.023–0.217	72.3%
	Olive-backed Euphonia	*Euphonia gouldi*	Tail feather	NI	2010	4	0.048 ± 0.022	0.026–0.076	45.3%
	Orange-bellied Euphonia	*Euphonia xanthogaster*	Body feather	PE	2019	1	1.101	—	—
	Yellow-throated Euphonia	*Euphonia hirundinacea*	Whole blood	BZ	2008–2021	7	0.003 ± 0.003	0.001–0.008	109.6%
			Body feather	BZ	2021	7	0.042 ± 0.029	0.010–0.096	70.2%
			Tail feather	BZ, CR, NI	2010–2021	8	0.083 ± 0.059	0.023–0.217	71.1%
Furnariidae (Ovenbirds and Woodcreepers)	All species	—	Whole blood	BZ, CO, CR	2007–2023	87	0.164 ± 0.175	0.001–0.721	106.4%

		Body feather	BZ, CO, PE	2018–2023	66	3.591 ± 5.359	0.229–28.494	149.2%
		Tail feather	BZ, CR, NI, PA, PE	2007–2021	63	1.447 ± 0.997	0.245–4.641	68.9%
Black-banded Woodcreeper	*Dendrocolaptes picumnus*	Body feather	PE	2019	1	1.507	—	—
Buff-throated Foliage-gleaner	*Automolus ochrolaemus*	Tail feather	PE	2018	1	1.850	—	—
Buff-throated Woodcreeper	*Xiphorhynchus guttatus*	Body feather	PE	2019	4	2.412 ± 0.885	1.502–3.625	36.7%
		Tail feather	PE	2018	1	0.615	—	—
Chestnut-crowned Foliage-gleaner	*Automolus rufipileatus*	Body feather	PE	2018–2019	3	1.277 ± 1.092	0.599–2.536	85.5%
		Tail feather	PE	2018	2	1.029 ± 0.542	0.646–1.412	52.6%
Chestnut-winged Hookbill	*Ancistrops strigilatus*	Body feather	PE	2019	1	2.320	—	—
		Tail feather	PE	2018	1	0.316	—	—
Cinnamon-rumped Foliage-gleaner	*Philydor pyrrhodes*	Tail feather	PE	2018	1	0.652	—	—
Cocoa Woodcreeper	*Xiphorhynchus susurrans*	Whole blood	CR	2010	4	0.055 ± 0.022	0.032–0.084	39.8%
Elegant Woodcreeper	*Xiphorhynchus elegans*	Body feather	PE	2019	6	3.866 ± 2.34	1.247–7.106	60.5%
		Tail feather	PE	2019	1	3.411	—	—
Ivory-billed Woodcreeper	*Xiphorhynchus flavigaster*	Whole blood	BZ	2021	1	0.156	—	—
		Body feather	BZ	2021	1	1.972	—	—
		Tail feather	BZ	2021	1	1.622	—	—
Long-tailed Woodcreeper	*Deconychura longicauda*	Body feather	PE	2019	2	1.346 ± 0.175	1.222–1.47	13.0%
		Tail feather	PE	2018	2	0.924 ± 0.187	0.792–1.056	20.2%
Northern Barred-Woodcreeper	*Dendrocolaptes sanctithomae*	Whole blood	CR	2010–2011	2	0.295 ± 0.038	0.268–0.322	12.9%
Ocellated Woodcreeper	*Xiphorhynchus ocellatus*	Body feather	PE	2019	3	1.058 ± 0.474	0.539–1.467	44.8%
		Tail feather	PE	2018	3	1.354 ± 1.127	0.245–2.499	83.2%
Olivaceous Woodcreeper	*Sittasomus griseicapillus*	Whole blood	BZ	2008–2019	2	0.112 ± 0.045	0.080–0.144	40.5%
		Tail feather	BZ, NI, PA, PE	2010–2019	6	1.102 ± 0.311	0.671–1.524	28.2%
Olive-backed Foliage-gleaner	*Automolus infuscatus*	Body feather	PE	2019	1	4.263	—	—
Pale-legged Hornero	*Furnarius leucopus*	Body feather	CO, PE	2019–2023	5	16.631 ± 10.243	3.769–28.494	61.6%
Plain Xenops	*Xenops minutus*	Whole blood	BZ	2008	1	0.045	—	—
		Body feather	BZ, PE	2019–2021	3	1.467 ± 0.855	—	58.3%
		Tail feather	BZ, NI, PE	2010–2021	5	0.788 ± 0.424	0.257–1.213	53.7%
Plain-brown Woodcreeper	*Dendrocincla fuliginosa*	Whole blood	CR	2010–2013	11	0.102 ± 0.061	0.050–0.241	59.6%

			Body feather	PE	2019	2	3.477 ± 0.229	3.315–3.639	6.6%
			Tail feather	PE	2018	3	1.994 ± 0.555	1.483–2.585	27.8%
	Plain-crowned Spinetail	*Synallaxis gujanensis*	Body feather	PE	2019	1	0.829	—	—
	Red-billed Scythebill	*Campylorhamphus trochilirostris*	Tail feather	PE	2018	1	1.771	—	—
	Ruddy Treerunner	*Margarornis rubiginosus*	Tail feather	CR	2010	1	1.869	—	—
	Ruddy Woodcreeper	*Dendrocincla homochroa*	Whole blood	BZ	2007–2021	14	0.382 ± 0.125	0.222–0.598	32.6%
			Body feather	BZ	2021	1	3.632	—	—
			Tail feather	BZ	2007–2021	3	3.719 ± 0.503	3.310–4.281	13.5%
	Rufous-breasted Spinetail	*Synallaxis erythrothorax*	Whole blood	BZ	2007–2021	4	0.061 ± 0.049	0.010–0.128	81.4%
			Body feather	BZ	2021	3	2.308 ± 2.41	0.574–5.060	104.4%
			Tail feather	BZ	2007–2021	3	0.700 ± 0.254	0.458–0.965	36.3%
	Spot-crowned Woodcreeper	*Lepidocolaptes affinis*	Tail feather	CR	2010	1	1.617	—	—
	Straight-billed Woodcreeper	*Dendroplex picus*	Body feather	CO, PE	2019–2023	5	7.182 ± 8.078	0.682–19.466	112.5%
			Tail feather	PA, PE	2011–2019	3	1.861 ± 1.405	0.465–3.275	75.5%
	Streak-headed Woodcreeper	*Lepidocolaptes souleyetii*	Whole blood	BZ	2008	2	0.145 ± 0.017	0.133–0.157	11.8%
			Tail feather	CR	2011	1	0.360	—	—
	Striped Woodcreeper	*Xiphorhynchus obsoletus*	Body feather	PE	2019	1	2.119	—	—
	Tawny-winged Woodcreeper	*Dendrocincla anabatina*	Whole blood	BZ, CR	2007–2021	20	0.211 ± 0.144	0.060–0.607	68.2%
			Body feather	BZ	2021	8	1.618 ± 1.425	0.604–4.965	88.1%
			Tail feather	BZ, CR	2007–2021	13	1.761 ± 1.009	0.670–4.641	57.3%
	Wedge-billed Woodcreeper	*Glyphorynchus spirurus*	Whole blood	BZ, CR	2008–2011	23	0.021 ± 0.056	0.004–0.278	267.1%
			Body feather	PE	2018–2019	8	0.654 ± 0.502	0.229–1.568	76.7%
			Tail feather	PE	2018–2019	8	0.720 ± 0.378	0.359–1.451	52.5%
	White-chinned Woodcreeper	*Dendrocincla merula*	Body feather	PE	2019	4	4.316 ± 1.511	2.196–5.552	35.0%
			Tail feather	PE	2018	2	2.558 ± 0.832	1.970–3.146	32.5%
	Woodcreeper sp.	*Xiphorhynchus sp.*	Body feather	PE	2019	1	1.283	—	—
	Yellow-chinned Spinetail	*Certhiaxis cinnamomeus*	Whole blood	CO	2023	2	0.670 ± 0.072	0.619–0.721	10.8%
			Body feather	CO	2023	2	1.891 ± 0.949	1.22–2.562	50.2%
Hirundinidae (Swallows)	All species	—	Whole blood	BZ, CO, NI	2008–2023	7	0.107 ± 0.136	0.004–0.361	127.5%
			Body feather	CO, PE	2019–2023	4	4.370 ± 1.726	2.887–6.774	39.5%
	Barn Swallow	*Hirundo rustica*	Whole blood	NI	2014	3	0.007 ± 0.003	0.004–0.009	37.7%
	Brown-chested Martin	*Progne tapera*	Whole blood	CO	2023	1	0.361	—	—
			Body feather	CO	2023	1	6.774	—	—

	Mangrove Swallow	*Tachycineta albilinea*	Whole blood	NI	2012	1	0.020	—	—
	Northern Rough-winged Swallow	*Stelgidopteryx serripennis*	Whole blood	BZ	2008–2009	2	0.173 ± 0.000	0.173–0.173	0%
	Southern Rough-winged Swallow	*Stelgidopteryx ruficollis*	Body feather	PE	2019	1	4.427	—	—
	White-banded Swallow	*Atticora fasciata*	Body feather	PE	2019	2	3.14 ± 0.358	2.887–3.394	11.4%
Icteridae (Troupials and Allies)	All species	—	Whole blood	BZ, MX, NI	2007–2021	30	0.054 ± 0.088	0.007–0.421	163.9%
			Body feather	BZ	2021	3	0.409 ± 0.224	0.166–0.606	54.7%
			Tail feather	BZ, NI	2007–2021	13	0.464 ± 0.503	0.100–1.654	108.5%
	Baltimore Oriole	*Icterus galbula*	Whole blood	BZ, NI	2010–2012	3	0.010 ± 0.002	0.008–0.012	21.5%
	Black-cowled Oriole	*Icterus prosthemelas*	Whole blood	BZ	2021	1	0.007	—	—
			Tail feather	BZ	2021	1	0.232	—	—
	Great-tailed Grackle	*Quiscalus mexicanus*	Whole blood	MX, NI	2012–2014	15	0.071 ± 0.108	0.011–0.421	150.9%
			Tail feather	NI	2014	5	0.781 ± 0.738	0.100–1.654	94.6%
	Melodious Blackbird	*Dives dives*	Tail feather	NI	2010	1	0.313	—	—
	Orchard Oriole	*Icterus spurius*	Whole blood	NI	2012	3	0.017 ± 0.004	0.014–0.021	22.7%
	Streak-backed Oriole	*Icterus pustulatus*	Whole blood	NI	2012	1	0.015	—	—
	Yellow-billed Cacique	*Amblycercus holosericeus*	Whole blood	BZ	2007–2021	7	0.064 ± 0.087	0.016–0.259	136.2%
			Body feather	BZ	2021	3	0.409 ± 0.224	0.166–0.606	54.7%
			Tail feather	BZ	2007–2021	6	0.263 ± 0.081	0.140–0.362	30.9%
Mimidae (Mockingbirds and Thrashers)	All species	—	Whole blood	BZ, DR, PR	2007–2017	16	0.029 ± 0.018	0.003–0.053	63.1%
			Tail feather	PR	2010	1	0.124	—	—
	Gray Catbird	*Dumetella carolinensis*	Whole blood	BZ	2007–2009	9	0.022 ± 0.012	0.012–0.048	52.1%
	Northern Mockingbird	*Mimus polyglottos*	Whole blood	DR	2017	5	0.051 ± 0.003	0.045–0.053	6.6%
	Pearly-eyed Thrasher	*Margarops fuscatus*	Whole blood	DR, PR	2010–2017	2	0.005 ± 0.003	0.003–0.007	49.9%
			Tail feather	PR	2010	1	0.124	—	—
Nesospingidae (Puerto Rican Tanager)	All species	—	Whole blood	PR	2010	2	0.004 ± 0.003	0.002–0.006	79.8%
			Tail feather	PR	2010	2	0.110 ± 0.05	0.075–0.146	45.4%
	Puerto Rican Tanager	*Nesospingus speculiferus*	Whole blood	PR	2010	2	0.004 ± 0.003	0.002–0.006	79.8%
			Tail feather	PR	2010	2	0.110 ± 0.05	0.075–0.146	45.4%
Oxyruncidae (Sharpbill, Royal Flycatcher, and Allies)	All species	—	Whole blood	BZ, CR	2008–2021	5	0.246 ± 0.287	0.014–0.662	116.7%

			Body feather	BZ	2021	1	0.663	—	—
			Tail feather	BZ, PE	2018–2021	3	0.467 ± 0.234	0.331–0.738	50.2%
	Royal Flycatcher	*Onychorhynchus coronatus*	Whole blood	BZ	2008–2021	3	0.368 ± 0.328	0.014–0.662	89.1%
			Body feather	BZ	2021	1	0.663	—	—
			Tail feather	BZ	2021	1	0.333	—	—
	Ruddy-tailed Flycatcher	*Terenotriccus erythrurus*	Whole blood	CR	2011	2	0.063 ± 0.052	0.026–0.100	83.1%
			Tail feather	PE	2018–2019	2	0.534 ± 0.288	0.331–0.738	53.8%
Parulidae (New World	All species	—	Whole blood	BZ, CO, CR, DR, MX, NI	2007–2023	294	0.180 ± 0.329	0.001–3.195	183.1%
			Tail feather	CR, NI	2010	5	0.490 ± 0.276	0.177—0.811	56.3%
	American Redstart	*Setophaga ruticilla*	Whole blood	BZ, NI, DR	2007–2017	12	0.286 ± 0.163	0.100–0.665	56.8%
	Black-and-white Warbler	*Mniotilta varia*	Whole blood	BZ, DR, MX	2007–2021	20	0.106 ± 0.052	0.033–0.257	48.9%
	Cape May Warbler	*Setophaga tigrina*	Whole blood	DR	2017	2	0.004 ± 0.003	0.002–0.006	70.7%
	Common Yellowthroat	*Geothlypis trichas*	Whole blood	BZ, DR, MX, NI	2007–2021	21	0.117 ± 0.083	0.015–0.400	70.6%
	Connecticut Warbler	*Oporornis agilis*	Whole blood	CR	2010	1	0.065	—	—
	Flame-throated Warbler	*Oreothlypis gutturalis*	Tail feather	CR	2010	1	0.177	—	—
	Golden-crowned Warbler	*Basileuterus culicivorus*	Tail feather	NI	2010	3	0.511 ± 0.266	0.304–0.811	52.1%
	Hooded Warbler	*Setophaga citrina*	Whole blood	BZ, DR	2007–2021	27	0.388 ± 0.623	0.047–2.659	160.9%
	Kentucky Warbler	*Geothlypis formosa*	Whole blood	BZ, CR	2007–2021	28	0.037 ± 0.016	0.014–0.085	43.4%
	Louisiana Waterthrush	*Parkesia motacilla*	Whole blood	BZ, CR, DR	2010–2021	10	0.285 ± 0.124	0.098–0.468	43.7%
	Magnolia Warbler	*Setophaga magnolia*	Whole blood	BZ	2007–2009	7	0.096 ± 0.055	0.041–0.208	58.6%
	Mourning Warbler	*Geothlypis philadelphia*	Whole blood	BZ	2021	1	0.028	—	—
	Northern Parula	*Setophaga americana*	Whole blood	DR	2017	2	0.174 ± 0.025	0.156–0.191	14.3%
	Northern Waterthrush	*Parkesia noveboracensis*	Whole blood	BZ, CR, DR, MX, NI	2007–2021	81	0.208 ± 0.252	0.001–1.804	121.3%
	Ovenbird	*Seiurus aurocapilla*	Whole blood	BZ, DR	2007–2021	19	0.076 ± 0.058	0.021–0.202	75.7%
	Prairie Warbler	*Setophaga discolor*	Whole blood	DR	2017	4	0.144 ± 0.058	0.086–0.224	40.4%
	Prothonotary Warbler	*Protonotaria citrea*	Whole blood	BZ, CO, CR, DR, NI	2008–2023	18	0.453 ± 0.809	0.04–3.195	178.5%
	Rufous-capped Warbler	*Basileuterus rufifrons*	Tail feather	NI	2010	1	0.741	—	—
	Swainson's Warbler	*Limnothlypis swainsonii*	Whole blood	BZ, DR	2017–2021	2	0.136 ± 0.102	0.064–0.208	74.9%
	Tennessee Warbler	*Leiothlypis peregrina*	Whole blood	BZ, NI	2009–2012	3	0.014 ± 0.007	0.009–0.022	52.6%
	Worm-eating Warbler	*Helmitheros vermivorum*	Whole blood	BZ, DR	2007–2021	9	0.077 ± 0.05	0.019–0.167	64.6%

	Yellow Warbler	*Setophaga petechia*	Whole blood	BZ, DR, NI	2009–2017	27	0.049 ± 0.043	0.009–0.151	88.5%
Passerellidae (New World Sparrows)	All species	—	Whole blood	BZ	2008–2021	18	0.034 ± 0.033	0.001–0.119	96.0%
			Body feather	BZ, PE	2018–2021	20	1.609 ± 4.129	0.154–19.02	256.7%
			Tail feather	BZ, CR, NI, PE	2010–2021	36	0.448 ± 0.411	0.047–1.773	91.5%
	Chestnut-capped Brush-Finch	*Arremon brunneinucha*	Tail feather	CR, NI	2010	2	1.394 ± 0.537	1.014–1.773	38.5%
	Common Chlorospingus	*Chlorospingus flavopectus*	Tail feather	CR, NI	2010	14	0.250 ± 0.264	0.089–1.140	105.7%
	Green-backed Sparrow	*Arremonops chloronotus*	Whole blood	BZ	2008–2021	18	0.034 ± 0.033	0.001–0.119	96.0%
			Body feather	BZ	2021	14	1.952 ± 4.939	0.154–19.02	253.0%
			Tail feather	BZ	2019–2021	15	0.535 ± 0.366	0.594–1.232	68.4%
	Olive Sparrow	*Arremonops rufivirgatus*	Body feather	BZ	2021	1	0.784	—	—
			Tail feather	BZ	2021	1	0.705	—	—
	Orange-billed Sparrow	*Arremon aurantiirostris*	Body feather	BZ	2021	1	0.193	—	—
			Tail feather	BZ	2021	1	0.232	—	—
	Pectoral Sparrow	*Arremon taciturnus*	Body feather	PE	2018	1	0.470	—	—
			Tail feather	PE	2018	1	0.658	—	—
	Rufous-collared Sparrow	*Zonotrichia capensis*	Tail feather	CR	2010	1	0.047	—	—
	White-naped Brush-Finch	*Atlapetes albinucha*	Tail feather	NI	2010	1	0.194	—	—
	Yellow-browed Sparrow	*Ammodramus aurifrons*	Body feather	PE	2019	3	1.132 ± 0.495	0.563–1.462	43.7%
Phaenicophilidae (Hispaniolan Tanagers)	All species	—	Whole blood	DR	2017	4	0.011 ± 0.006	0.004–0.019	58.9%
	Black-crowned Palm-Tanager	*Phaenicophilus palmarum*	Whole blood	DR	2017	4	0.011 ± 0.006	0.004–0.019	58.9%
Pipridae (Manakins)	All species	—	Whole blood	BZ	2007–2021	39	0.008 ± 0.013	0.001–0.080	166.7%
			Body feather	BZ, PE	2019–2021	85	0.471 ± 0.345	0.013–1.240	73.3%
			Tail feather	BZ, CR, NI, PE	2007–2021	82	0.373 ± 0.383	0.008–2.755	102.7%
	Band-tailed Manakin	*Pipra fasciicauda*	Body feather	PE	2019	56	0.576 ± 0.329	0.113–1.24	57.1%
			Tail feather	PE	2017–2019	48	0.331 ± 0.248	0.056–1.054	75.0%
	Blue-crowned Manakin	*Lepidothrix coronata*	Body feather	PE	2019	2	0.973 ± 0.175	0.849–1.097	18.0%
			Tail feather	PE	2018	1	1.653	—	—
	Fiery-capped Manakin	*Machaeropterus pyrocephalus*	Body feather	PE	2019	2	0.671 ± 0.357	0.418–0.924	53.2%
	Red-capped Manakin	*Ceratopipra mentalis*	Whole blood	BZ	2007–2021	18	0.009 ± 0.006	0.001–0.024	67.1%
			Body feather	BZ	2019–2021	8	0.228 ± 0.125	0.017–0.391	55.1%

			Tail feather	BZ, CR, NI	2007–2021	10	0.593 ± 0.776	0.017–2.755	130.9%
	Round-tailed Manakin	*Ceratopipra chloromeros*	Body feather	PE	2019	1	0.248	—	—
	White-collared Manakin	*Manacus candei*	Whole blood	BZ	2007–2021	21	0.007 ± 0.017	0.001–0.080	253.5%
			Body feather	BZ	2021	15	0.157 ± 0.173	0.013–0.568	110.5%
			Tail feather	BZ, CR	2007–2021	23	0.31 ± 0.241	0.008–0.893	77.8%
	White-crowned Manakin	*Manacus candei*	Body feather	BZ	2019	1	0.071	—	—
Polioptilidae (Gnatcatchers)	All species	—	Whole blood	BZ	2007–2021	4	0.062 ± 0.054	0.016–0.141	88.1%
			Body feather	BZ	2021	1	0.427	—	—
			Tail feather	BZ	2007–2021	2	0.485 ± 0.384	0.213–0.756	79.3%
	Long-billed Gnatwren	*Ramphocaenus melanurus*	Whole blood	BZ	2007–2009	4	0.062 ± 0.054	0.016–0.141	88.1%
			Body feather	BZ	2021	1	0.427	—	—
			Tail feather	BZ	2007–2021	2	0.485 ± 0.384	0.031–0.036	79.3%
Spindalidae (Spindalises)	All species	—	Whole blood	PR	2010	2	0.001 ± 0.000	0.001–0.001	0.0%
			Tail feather	PR	2010	2	0.034 ± 0.004	0.031–0.036	10.6%
	Puerto Rican Spindalis	*Spindalis portoricensis*	Whole blood	PR	2010	2	0.001 ± 0.000	0.001–0.001	0.0%
			Tail feather	PR	2010	2	0.034 ± 0.004	0.031–0.036	10.6%
Thamnophilidae (Typical Antbirds)	All species	—	Whole blood	BZ, CO, CR	2007–2023	18	0.124 ± 0.214	0.001–0.791	172.4%
			Body feather	BZ, CO, PE	2018–2023	61	3.170 ± 3.563	0.127–17.802	112.4%
			Tail feather	BZ, CR, NI, PE	2007–2021	75	2.346 ± 2.067	0.001–8.606	88.1%
	Antwren sp.	*Myrmotherula sp.*	Body feather	PE	2019	1	0.292	—	—
	Band-tailed Antbird	*Hypocnemoides maculicauda*	Tail feather	PE	2018	2	2.192 ± 1.437	1.176–3.208	65.5%
	Barred Antshrike	*Thamnophilus doliatus*	Whole blood	BZ, CR	2008–2021	4	0.058 ± 0.076	0.001–0.167	131.1%
			Body feather	BZ	2021	2	0.276 ± 0.211	0.127–0.426	76.4%
			Tail feather	BZ, CR	2011–2021	3	0.158 ± 0.158	0.001–0.317	100.2%
	Black-crowned Antshrike	*Thamnophilus atrinucha*	Whole blood	CR	2010	1	0.040	—	—
	Black-faced Antbird	*Myrmoborus myotherinus*	Body feather	PE	2019	1	2.216	—	—
			Tail feather	PE	2018	1	0.957	—	—
	Black-hooded Antshrike	*Thamnophilus bridgesi*	Tail feather	CR	2011	1	0.032	—	—
	Black-spotted Bare-eye	*Phlegopsis nigromaculata*	Body feather	PE	2018–2019	8	5.219 ± 2.045	2.548–7.465	39.2%
			Tail feather	PE	2018–2019	9	3.967 ± 2.106	0.930–6.737	53.1%
	Bluish-slate Antshrike	*Thamnomanes schistogynus*	Tail feather	PE	2018	1	2.739	—	—
	Checker-throated Stipplethroat	*Epinecrophylla fulviventris*	Tail feather	CR	2010	1	0.419	—	—

Chestnut-tailed Antbird	*Sciaphylax hemimelaena*	Body feather	PE	2019	1	0.810	—	—
		Tail feather	PE	2018–2019	4	1.848 ± 1.288	0.564–3.559	69.7%
Common Scale-backed Antbird	*Willisornis poecilinotus*	Tail feather	PE	2018	6	5.268 ± 2.414	2.328–8.038	45.8%
Dot-winged Antwren	*Microrhopias quixensis*	Body feather	BZ	2021	1	1.086	—	—
		Tail feather	BZ	2021	1	1.717	—	—
Dusky Antbird	*Cercomacroides tyrannina*	Whole blood	BZ	2007–2021	10	0.048 ± 0.023	0.013–0.095	48.046
		Body feather	BZ	2021	2	0.425 ± 0.211	0.275–0.574	49.706
		Tail feather	BZ	2007–2021	5	0.533 ± 0.183	0.224–0.697	34.224
Dusky-throated Antshrike	*Thamnomanes ardesiacus*	Body feather	PE	2019	4	2.761 ± 1.570	1.061–4.224	56.9%
		Tail feather	PE	2018–2019	3	1.224 ± 0.013	1.217–1.239	1.0%
Gray Antwren	*Myrmotherula menetriesii*	Tail feather	PE	2018	1	1.279	—	—
Great Antshrike	*Taraba major*	Whole blood	BZ	2021	1	0.09	—	—
		Body feather	BZ, PE	2019–2021	3	0.928 ± 0.54	0.307–1.283	58.2%%
		Tail feather	BZ, PE	2018–2021	2	1.968 ± 0.907	1.327–2.610	46.1%
Ihering's Antwren	*Myrmotherula iheringi*	Body feather	PE	2019	2	1.385 ± 0.528	1.012–1.758	38.1%
Long-winged Antwren	*Myrmotherula longipennis*	Body feather	PE	2019	3	3.186 ± 2.073	1.061–5.203	65.1%
		Tail feather	PE	2018–2019	5	1.513 ± 0.476	1.065–2.204	31.4%
Plain-throated Antwren	*Isleria hauxwelli*	Body feather	PE	2018–2019	12	3.807 ± 3.621	0.736–12.130	95.1%
		Tail feather	PE	2018–2019	4	2.154 ± 0.647	1.576–2.945	30.0%
Plain-winged Antshrike	*Thamnophilus schistaceus*	Body feather	PE	2019	2	0.426 ± 0.106	0.351–0.501	25.0%
		Tail feather	PE	2019	1	0.107	—	—
Plumbeous Antbird	*Myrmelastes hyperythrus*	Body feather	PE	2018–2019	9	1.645 ± 0.489	1.123–2.463	29.7%
		Tail feather	PE	2018	1	1.035	—	—
Rufous-backed Stipplethroat	*Epinecrophylla haematonota*	Tail feather	PE	2018	4	1.310 ± 0.531	0.845–1.980	40.5%
Slaty Antwren	*Myrmotherula schisticolor*	Tail feather	NI	2010	1	1.357	—	—
Sooty Antbird	*Hafferia fortis*	Tail feather	PE	2018	2	2.886 ± 0.220	2.730–3.041	7.6%
Spot-winged Antshrike	*Pygiptila stellaris*	Body feather	PE	2019	1	1.116	—	—
Streak-crowned Antvireo	*Dysithamnus striaticeps*	Tail feather	CR	2010	1	0.979	—	—
White-browed Antbird	*Myrmoborus leucophrys*	Body feather	PE	2019	3	1.216 ± 1.330	0.436–2.752	109.3%
		Tail feather	PE	2018	4	1.842 ± 1.138	0.448–3.069	61.8%
White-flanked Antwren	*Myrmotherula axillaris*	Body feather	PE	2019	1	4.052	—	—
		Tail feather	PE	2019	2	0.913 ± 0.391	0.636–1.189	42.9%
White-fringed Antwren	*Formicivora grisea*	Whole blood	CO	2023	2	0.696 ± 0.135	0.600–0.791	19.4%

			Body feather	CO	2023	2	14.588 ± 4.545	11.374–17.802	31.2%
	White-shouldered Antbird	*Akletos melanoceps*	Tail feather	PE	2018	2	4.623 ± 1.346	3.671–5.575	29.1%
	White-shouldered Antshrike	*Thamnophilus aethiops*	Body feather	PE	2019	1	0.956	—	—
	White-throated Antbird	*Oneillornis salvini*	Body feather	PE	2019	2	9.672 ± 5.551	5.747–13.598	57.4%
			Tail feather	PE	2018–2019	8	3.732 ± 2.979	1.100–8.606	79.8%
Thraupidae (Tanagers and Allies)	All species	—	Whole blood	BZ, CO, DR, NI, PR	2007–2023	86	0.067 ± 0.11	0.001–0.509	164.3%
			Body feather	BZ, CO, PE	2019–2023	61	1.074 ± 0.998	0.001–4.659	93.0%
			Tail feather	BZ, CR, NI, PE, PR	2007–2021	93	0.385 ± 0.694	0.001–3.349	180.3%
	Bananaquit	*Coereba flaveola*	Whole blood	DR, PR	2010–2017	23	0.007 ± 0.007	0.001–0.032	99.7%
			Tail feather	PR	2010	19	0.130 ± 0.111	0.037–0.533	85.9%
	Black-billed Seed-Finch	*Sporophila atrirostris*	Body feather	PE	2019	1	1.256	—	—
	Black-headed Saltator	*Saltator atriceps*	Tail feather	NI	2010	2	0.171 ± 0.049	0.136–0.206	28.9%
	Blue-black Grassquit	*Volatinia jacarina*	Whole blood	NI	2012	1	0.012	—	—
			Body feather	PE	2019	4	0.294 ± 0.145	0.150–0.493	49.4%
	Blue-gray Tanager	*Thraupis episcopus*	Whole blood	CO	2023	1	0.059	—	—
			Body feather	CO, PE	2019–2023	3	1.056 ± 0.587	0.609–1.721	55.6%
			Tail feather	CR	2011	1	0.001	—	—
	Buff-throated Saltator	*Saltator maximus*	Whole blood	BZ, CR	2009–2011	2	0.001 ± 0.000	0.001–0.001	0.0%
			Body feather	PE	2019	1	0.745	—	—
			Tail feather	CR, NI, PE	2010–2018	6	0.124 ± 0.093	0.008–0.224	75.1%
	Chestnut-bellied Seed-Finch	*Sporophila angolensis*	Body feather	PE	2019	2	0.529 ± 0.209	0.381–0.677	39.6%
	Chestnut-bellied Seedeater	*Sporophila castaneiventris*	Body feather	PE	2019	4	0.348 ± 0.112	0.230–0.490	32.1%
	Crimson-collared Tanager	*Ramphocelus sanguinolentus*	Tail feather	NI	2010	2	0.090 ± 0.015	0.079–0.100	16.6%
	Golden-hooded Tanager	*Stilpnia larvata*	Tail feather	NI	2010	1	0.131	—	—
	Gray-headed Tanager	*Eucometis penicillata*	Whole blood	BZ	2007–2021	24	0.251 ± 0.110	0.083–0.509	51.3%
			Body feather	BZ	2021	9	2.589 ± 0.599	1.605–4.659	35.1%
			Tail feather	BZ, NI	2007–2021	14	1.879 ± 0.599	1.244–3.349	31.8%
	Grayish Saltator	*Saltator coerulescens*	Body feather	PE	2019	1	0.319	—	—
	Green-and-gold Tanager	*Tangara schrankii*	Body feather	PE	2019	5	0.437 ± 0.372	0.164–1.073	85.1%
			Tail feather	PE	2018–2019	2	0.219 ± 0.153	0.111–0.327	69.7%
	Morelet's Seedeater	*Sporophila morelleti*	Whole blood	BZ	2008–2009	16	0.016 ± 0.021	0.001–0.091	129.4%

			Body feather	BZ	2021	3	0.045 ± 0.024	0.018–0.06	52.5%
			Tail feather	BZ	2021	5	0.065 ± 0.052	0.015–0.130	80.2%
	Puerto Rican Bullfinch	*Melopyrrha portoricensis*	Whole blood	PR	2010	3	0.004 ± 0.001	0.002–0.005	31.1%
			Tail feather	PR	2010	3	0.055 ± 0.028	0.032–0.087	51.4%
	Red-legged Honeycreeper	*Cyanerpes cyaneus*	Tail feather	BZ	2021	1	0.188	—	—
	Ruddy-breasted	*Sporophila minuta*	Tail feather	NI	2014	1	0.001	—	—
	Scarlet-rumped Tanager	*Ramphocelus passerinii*	Whole blood	CR, NI	2011–2014	4	0.010 ± 0.018	0.001–0.037	180.0%
			Tail feather	CR, NI	2010–2014	8	0.119 ± 0.179	0.016–0.55	150.8%
	Silver-beaked Tanager	*Ramphocelus carbo*	Body feather	PE	2019	19	1.454 ± 0.565	0.197–2.921	38.8%
			Tail feather	PE	2018	1	0.414	—	—
	Slate-colored Seedeater	*Sporophila schistacea*	Whole blood	CO	2023	1	0.017	—	—
			Body feather	CO	2023	1	0.206	—	—
	Thick-billed Seed-Finch	*Sporophila funerea*	Whole blood	BZ	2009–2021	7	0.002 ± 0.002	0.001–0.007	127.1%
			Body feather	BZ	2021	5	0.021 ± 0.016	0.003–0.046	78.3%
			Tail feather	BZ, CR	2011–2021	9	0.063 ± 0.100	0.001–0.326	158.7%
	Variable Seedeater	*Sporophila corvina*	Whole blood	BZ	2021	1	0.001	—	—
			Body feather	BZ	2021	2	0.002 ± 0.002	0.001–0.003	75.0%
			Tail feather	BZ, CR, NI	2010–2021	16	0.035 ± 0.054	0.001–0.198	153.9%
	White-shouldered Tanager	*Loriotus luctuosus*	Tail feather	PE	2017–2018	2	1.006 ± 0.540	0.624–1.387	53.7%
	White-winged Shrike-Tanager	*Lanio versicolor*	Body feather	PE	2019	1	2.819	—	—
Tityridae (Tityras and Allies)	All species	—	Whole blood	BZ	2007–2021	4	0.014 ± 0.024	0.001–0.050	170.2%
			Body feather	BZ, PE	2019–2021	5	0.496 ± 0.747	0.014–1.791	150.5%
			Tail feather	BZ, PE	2007–2021	4	0.085 ± 0.092	0.014–0.221	108.1%
	Cinereous Mourner	*Laniocera hypopyrra*	Body feather	PE	2019	1	0.100	—	—
	Northern Schiffornis	*Schiffornis veraepacis*	Whole blood	BZ	2008–2021	3	0.002 ± 0.001	0.001–0.003	57.0%
			Body feather	BZ	2021	2	0.052 ± 0.054	0.014–0.09	102.976
			Tail feather	BZ	2021	2	0.030 ± 0.023	0.014–0.046	77.663
	Pink-throated Becard	*Pachyramphus minor*	Body feather	PE	2019	1	1.791	—	—
	Rose-throated Becard	*Pachyramphus aglaiae*	Whole blood	BZ	2007	1	0.050	—	—
			Tail feather	BZ	2007	1	0.221	—	—
	Varzea Schiffornis	*Schiffornis major*	Body feather	PE	2019	1	0.486	—	—
			Tail feather	PE	2019	1	0.061	—	—
Troglodytidae	All species	—	Whole blood	BZ, CO, NI	2007–2023	43	0.119 ± 0.149	0.001–0.909	125.3%

(Wrens)									
			Body feather	BZ, CO, PE	2019–2023	25	2.096 ± 2.701	0.208–12.841	128.9%
			Tail feather	BZ, CR, NI, PA, PE	2007–2021	39	1.330 ± 1.169	0.023–4.632	87.9%
	Bicolored Wren	*Campylorhynchus griseus*	Whole blood	CO	2023	1	0.364	—	—
			Body feather	CO	2023	1	12.841	—	—
	Cabanis's Wren	*Cantorchilus modestus*	Tail feather	NI	2010	1	0.499	—	—
	House Wren	*Troglodytes aedon*	Whole blood	CO	2023	1	0.184	—	—
			Body feather	CO, PE	2019–2023	5	3.372 ± 1.878	1.526–6.393	55.7%
			Tail feather	CR, PA	2011	8	2.690 ± 1.500	0.374–4.632	55.8%
	Moustached Wren	*Pheugopedius genibarbis*	Body feather	PE	2019	1	3.542	—	—
	Musician Wren	*Cyphorhinus arada*	Body feather	PE	2019	3	2.199 ± 0.419	1.848–2.663	19.1%
			Tail feather	PE	2018	1	2.327	—	—
	Riverside Wren	*Cantorchilus semibadius*	Whole blood	CR	2011	1	0.001	—	—
			Tail feather	CR	2011	1	0.023	—	—
	Rufous-and-white Wren	*Thryophilus rufalbus*	Whole blood	NI	2012	1	0.132	—	—
	Rufous-naped Wren	*Campylorhynchus rufinucha*	Whole blood	NI	2012	4	0.079 ± 0.011	0.069–0.094	13.9%
	Scaly-breasted Wren	*Microcerculus marginatus*	Body feather	PE	2019	1	3.154	—	—
			Tail feather	PE	2018	1	0.859	—	—
	Spot-breasted Wren	*Pheugopedius maculipectus*	Whole blood	BZ	2007–2021	23	0.081 ± 0.073	0.009–0.289	89.333
			Body feather	BZ	2021	10	0.579 ± 0.38	0.260–1.500	65.642
			Tail feather	BZ	2007–2021	15	0.782 ± 0.505	0.277–1.745	64.571
	Stripe-breasted Wren	*Cantorchilus thoracicus*	Tail feather	CR	2010	1	2.215	—	—
	White-bellied Wren	*Uropsila leucogastra*	Whole blood	BZ	2007–2021	4	0.149 ± 0.095	0.208–0.24	63.9%
			Body feather	BZ	2021	3	0.339 ± 0.117	0.208–0.433	34.5%
			Tail feather	BZ	2007–2021	6	1.009 ± 1.165	0.265–3.257	115.4%
	White-breasted Wood-Wren	*Henicorhina leucosticta*	Whole blood	BZ	2009–2021	8	0.204 ± 0.294	0.053–0.909	143.9%
			Body feather	BZ	2021	1	2.595		
			Tail feather	BZ, NI	2010–2021	5	1.327 ± 0.618	0.798–2.385	46.6%%
Turdidae (Thrushes and Allies)	All species	—	Whole blood	BZ, CO, CR, DR, NI, PR	2007–2023	34	0.025 ± 0.03	0.001–0.117	118.0%
			Body feather	BZ, CO, PE	2019–2023	20	0.744 ± 0.675	0.092–2.881	90.7%
			Tail feather	BZ, CR, NI, PE, PR	2010–2021	44	0.444 ± 0.441	0.026–1.703	99.2%
	Black-billed Thrush	*Turdus ignobilis*	Body feather	PE	2019	5	0.688 ± 0.319	0.379–1.223	46.4%
	Black-headed Nightingale-Thrush	*Catharus mexicanus*	Tail feather	NI	2010	2	1.348 ± 0.008	1.343–1.354	0.6%

	Clay-colored Thrush	*Turdus grayi*	Whole blood	BZ, CO, CR, NI	2010–2023	18	0.022 ± 0.025	0.001–0.085	113.8%
			Body feather	BZ, CO	2021–2023	10	0.400 ± 0.27	0.092–1.008	67.5%
			Tail feather	BZ, CR, NI	2010–2021	35	0.347 ± 0.318	0.058–1.703	91.5%
	Hauxwell's Thrush	*Turdus hauxwelli*	Body feather	PE	2019	5	1.489 ± 0.951	0.354–2.881	63.8%
			Tail feather	PE	2019	1	0.690	—	—
	Red-legged Thrush	*Turdus plumbeus*	Whole blood	DR, PR	2010–2017	3	0.039 ± 0.023	0.013–0.056	58.5%
			Tail feather	PR	2010	1	0.258	—	—
	Slate-colored Solitaire	*Myadestes unicolor*	Tail feather	NI	2010	2	0.080 ± 0.077	0.026–0.135	95.7%
	Swainson's Thrush	*Catharus ustulatus*	Whole blood	BZ, CR, NI	2011–2021	8	0.003 ± 0.002	0.001–0.007	73.0%
	Veery	*Catharus fuscescens*	Whole blood	BZ	2021	1	0.025	—	—
	White-necked Thrush	*Turdus albicollis*	Tail feather	PE	2018–2019	3	1.197 ± 0.669	0.436–1.694	55.9%
	White-throated Thrush	*Turdus assimilis*	Whole blood	BZ	2010	1	0.038	—	—
	Wood Thrush	*Hylocichla mustelina*	Whole blood	BZ	2007–2009	3	0.084 ± 0.032	0.054–0.117	37.7%
Tyrannidae (Tyrant Flycatchers)	All species	—	Whole blood	BZ, CO, DR, MX, NI	2007–2023	106	0.084 ± 0.142	0.001–0.978	169.5%
			Body feather	BZ, CO, PE	2019–2023	45	1.746 ± 2.401	0.038–13.940	137.5%
			Tail feather	BZ, CR, MX, NI, PA, PE	2007–2021	71	0.894 ± 0.961	0.001–5.165	107.5%
	Acadian Flycatcher	*Empidonax virescens*	Whole blood	BZ	2008–2021	5	0.172 ± 0.034	0.136–0.216	19.6%
	Black Phoebe	*Sayornis nigricans*	Whole blood	NI	2014	1	0.699	—	—
			Tail feather	NI	2014	1	1.639	—	—
	Bran-colored Flycatcher	*Myiophobus fasciatus*	Tail feather	PE	2018	1	5.165	—	—
	Bright-rumped Attila	*Attila spadiceus*	Whole blood	BZ	2007	3	0.07 ± 0.041	0.025–0.107	59.1%
			Body feather	BZ, PE	2019–2021	3	1.798 ± 1.561	0.774–3.595	86.8%
			Tail feather	BZ, PE	2007–2021	3	2.271 ± 0.774	1.693–3.151	34.1%
	Brown-crested Flycatcher	*Myiarchus tyrannulus*	Whole blood	NI	2012–2014	3	0.023 ± 0.019	0.006–0.044	82.4%
			Tail feather	NI	2014	1	0.213	—	—
	Common Tody-Flycatcher	*Todirostrum cinereum*	Whole blood	NI	2012	1	0.081	—	—
			Tail feather	CR, PA	2011	2	0.207 ± 0.292	0.001–0.414	140.7%
	Dusky-capped Flycatcher	*Myiarchus tuberculifer*	Whole blood	BZ	2008–2021	3	0.036 ± 0.004	0.031–0.039	11.9%
			Body feather	BZ	2021	2	1.038 ± 0.021	1.023–1.053	1.9%
			Tail feather	BZ	2021	2	0.515 ± 0.14	0.416–0.615	27.2%
	Eastern Wood-Pewee	*Contopus virens*	Whole blood	BZ	2021	1	0.484	—	—
	Eye-ringed Flatbill	*Rhynchocyclus brevirostris*	Whole blood	BZ	2019	2	0.066 ± 0.042	0.036–0.096	64.3%

		Tail feather	BZ, NI	2010–2019	3	0.567 ± 0.083	0.509–0.662	14.6%
Flammulated Pygmy-Tyrant	*Hemitriccus flammulatus*	Body feather	PE	2019	1	0.823	—	—
Flammulated Pygmy-Tyrant	*Hemitriccus flammulatus*	Tail feather	PE	2018	1	4.177	—	—
Flatbill sp.	*Ramphotrigon sp.*	Tail feather	PE	2018–2019	2	1.139 ± 0.893	0.508–1.771	78.4%
Golden-crowned Spadebill	*Platyrinchus coronatus*	Body feather	PE	2019	1	0.700	—	—
		Tail feather	PE	2018	2	0.949 ± 0.270	0.758–1.140	28.5%
Gray Kingbird	*Tyrannus dominicensis*	Whole blood	DR	2017	1	0.009	—	—
Great Kiskadee	*Pitangus sulphuratus*	Whole blood	BZ, MX, NI	2012–2021	16	0.035 ± 0.033	0.006–0.094	93.9%
		Body feather	BZ	2021	4	1.393 ± 1.155	0.245–2.908	82.9%
		Tail feather	BZ, CR, MX, NI	2011–2021	25	0.651 ± 0.552	0.063–2.392	84.8%
Greenish Elaenia	*Myiopagis viridicata*	Whole blood	BZ	2008–2021	2	0.011 ± 0.014	0.001–0.020	128.0%
		Body feather	BZ	2021	1	0.038	—	—
		Tail feather	BZ	2021	1	0.103	—	—
Hispaniolan Pewee	*Contopus hispaniolensis*	Whole blood	DR	2017	3	0.311 ± 0.090	0.209–0.378	28.9%
Lesser Elaenia	*Elaenia chiriquensis*	Whole blood	CO	2023	1	0.028	—	—
		Body feather	CO	2023	1	1.092	—	—
Mountain Elaenia	*Elaenia frantzii*	Tail feather	CR	2010	1	0.100	—	—
Northern Beardless-Tyrannulet	*Camptostoma imberbe*	Tail feather	BZ	2021	1	0.199	—	—
Northern Bentbill	*Oncostoma cinereigulare*	Whole blood	BZ	2007–2021	5	0.069 ± 0.035	0.041–0.113	50.6%
		Body feather	BZ	2021	3	0.786 ± 0.185	0.574–0.919	23.6%
		Tail feather	BZ	2007–2021	4	1.217 ± 0.796	0.532–2.367	65.4%
Ochre-bellied Flycatcher	*Mionectes oleagineus*	Whole blood	BZ	2008–2011	28	0.031 ± 0.050	0.009–0.278	162.8%
		Body feather	BZ, PE	2019	2	0.831 ± 0.330	0.597–1.064	39.8%
		Tail feather	CR, NI, PE	2010–2017	9	0.591 ± 0.415	0.068–1.302	70.2%
Olivaceous Flatbill	*Rhynchocyclus olivaceus*	Body feather	PE	2019	4	1.668 ± 0.876	0.967–2.923	52.5%
Pied Water-Tyrant	*Fluvicola pica*	Whole blood	CO	2023	1	0.978	—	—
		Body feather	CO	2023	1	7.840	—	—
Ringed Antpipit	*Corythopis torquatus*	Body feather	PE	2019	2	1.079 ± 0.016	1.068–1.091	1.5%
Rusty-margined Flycatcher	*Myiozetetes cayanensis*	Whole blood	CO	2023	2	0.092 ± 0.058	0.051–0.133	63.0%
		Body feather	CO	2023	2	2.118 ± 0.596	1.697–2.540	28.1%
Sepia-capped Flycatcher	*Leptopogon amaurocephalus*	Body feather	PE	2019	1	0.724	—	—
		Tail feather	PE	2018	3	0.999 ± 0.504	0.417–1.292	50.5%

	Slate-headed Tody-flycatcher	*Poecilotriccus sylvia*	Whole blood	CO	2023	1	0.472	—	—
			Body feather	CO	2023	1	13.94	—	—
	Slender-footed Tyrannulet	*Zimmerius gracilipes*	Body feather	PE	2019	1	0.716	—	—
	Social Flycatcher	*Myiozetetes similis*	Whole blood	BZ, CO, NI	2009–2023	4	0.051 ± 0.054	0.005–0.125	106.4%
			Body feather	CO, PE	2019–2023	4	1.650 ± 0.817	0.934–2.699	49.5%
	Southern Mouse-colored Tyrannulet	*Nesotriccus murina*	Body feather	PE	2019	1	1.231	—	—
	Stub-tailed Spadebill	*Platyrinchus cancrominus*	Tail feather	NI	2010	1	0.618	—	—
	Sulphur-bellied Flycatcher	*Myiodynastes luteiventris*	Whole blood	BZ	2019	2	0.057 ± 0.023	0.041–0.073	39.7%
	Tody-Tyrant sp.	*Hemitriccus sp.*	Tail feather	PE	2018	1	2.739	—	—
	Traill's Flycatcher	*Empidonax sp.*	Whole blood	CR	2011	1	0.001	—	—
	Tropical Kingbird	*Tyrannus melancholicus*	Whole blood	BZ, CO, NI	2008–2023	5	0.104 ± 0.119	0.020–0.306	114.2%
			Body feather	CO	2023	2	5.280 ± 0.118	5.196–5.364	2.2%
			Tail feather	NI	2012	2	0.785 ± 0.550	0.396–1.174	70.1%
	Tropical Pewee	*Contopus cinereus*	Whole blood	BZ	2009	3	0.174 ± 0.042	0.126–0.203	24.0%
	Western Wood-Pewee	*Contopus sordidulus*	Whole blood	NI	2014	1	0.187	—	—
	White-bellied Tody-Tyrant	*Hemitriccus griseipectus*	Body feather	PE	2019	1	0.349	—	—
			Tail feather	PE	2018	1	2.753	—	—
	Wing-barred Piprites	*Piprites chloris*	Tail feather	PE	2018	1	0.385	—	—
	Yellow-bellied Elaenia	*Elaenia flavogaster*	Whole blood	BZ, CO, NI	2009–2023	7	0.004 ± 0.003	0.001–0.010	81.8%
			Body feather	BZ, CO	2021–2023	3	0.143 ± 0.153	0.049–0.319	106.6%
			Tail feather	BZ	2021	1	0.051	—	—
	Yellow-bellied Flycatcher	*Empidonax flaviventris*	Whole blood	BZ	2007–2021	2	0.114 ± 0.018	0.101–0.126	15.4%
	Yellow-crowned Tyrannulet	*Tyrannulus elatus*	Body feather	PE	2019	3	1.097 ± 0.551	0.612–1.696	50.3%
	Yellow-olive Flycatcher	*Tolmomyias sulphurescens*	Whole blood	BZ	2007–2021	2	0.033 ± 0.046	0.001–0.066	137.2%
			Body feather	BZ	2021	1	0.118	—	—
			Tail feather	BZ	2007–2021	2	0.128 ± 0.051	0.092–0.165	39.8%
Vireonidae (Vireos, Shrike-Babblers, and Erpornis)	All species	—	Whole blood	BZ, DR, PR	2007–2021	31	0.062 ± 0.08	0.005–0.315	130.2%
			Body feather	BZ	2021	3	1.033 ± 0.603	0.627–1.726	58.4%
			Tail feather	BZ, CR, PR	2007–2021	9	0.930 ± 0.502	0.228–1.937	53.9%
	Black-whiskered Vireo	*Vireo altiloquus*	Whole blood	DR, PR	2010–2017	3	0.036 ± 0.027	0.005–0.052	74.5%

		Lesser Greenlet	*Pachysylvia decurtata*	Whole blood	BZ	2021	1	0.084	—	—
				Body feather	BZ	2021	1	1.726	—	—
				Tail feather	BZ	2021	1	0.911	—	—
		Mangrove Vireo	*Vireo pallens*	Whole blood	BZ	2007–2021	8	0.154 ± 0.113	0.019–0.315	73.3%
				Body feather	BZ	2021	2	0.687 ± 0.084	0.627–0.746	12.2%
				Tail feather	BZ	2007–2021	5	1.045 ± 0.667	0.228–1.937	63.8%
		Red-eyed Vireo	*Vireo olivaceus*	Whole blood	BZ	2008–2009	2	0.025 ± 0.000	0.025–0.025	0%
		White-eyed Vireo	*Vireo griseus*	Whole blood	BZ	2008–2009	3	0.057 ± 0.038	0.018–0.093	66.8%
		Yellow-green Vireo	*Vireo flavoviridis*	Whole blood	BZ	2008–2021	14	0.019 ± 0.008	0.007–0.035	44.4%
		Yellow-winged Vireo	*Vireo carmioli*	Tail feather	CR	2010	3	0.745 ± 0.180	0.537–0.849	24.2%
Pelecaniformes	All families	—	—	Whole blood	DR	2017	6	0.151 ± 0.088	0.053–0.281	57.9%
				Tail feather	NI	2014	1	0.447	—	—
	Ardeidae (Herons, Egrets, and Bitterns)	All species	—	Whole blood	DR	2017	6	0.151 ± 0.088	0.053–0.281	57.9%
				Tail feather	NI	2014	1	0.447	—	—
		Cattle Egret	*Bubulcus ibis*	Tail feather	NI	2014	1	0.447	—	—
		Green Heron	*Butorides virescens*	Whole blood	DR	2017	6	0.151 ± 0.088	0.053–0.281	57.9%
Piciformes	All families	—	—	Whole blood	BZ, CO, DR, MX, NI	2013–2023	9	0.027 ± 0.058	0.001–0.179	213.6%
				Body feather	BZ, PE	2019–2023	24	0.747 ± 2.009	0.008–9.721	268.9%
				Tail feather	BZ, CR, NI, PE	2010–2021	5	0.094 ± 0.067	0.033–0.205	72.0%
	Capitonidae (New World Barbets)	All species	—	Body feather	PE	2019	1	0.808	—	—
		Gilded Barbet	*Capito auratus*	Body feather	PE	2019	1	0.808	—	—
	Picidae (Woodpeckers)	All species	—	Whole blood	BZ, CO, DR, MX, NI	2013–2023	9	0.027 ± 0.058	0.001–0.179	213.6%
				Body feather	BZ, CO, PE	2019–2023	13	1.161 ± 2.701	0.008–9.721	232.7%
				Tail feather	BZ, CR, NI, PE	2010–2021	5	0.094 ± 0.067	0.033–0.205	72.0%
		Golden-cheeked Woodpecker	*Melanerpes chrysogenys*	Whole blood	MX	2013	1	0.026	—	—
		Golden-fronted Woodpecker	*Melanerpes aurifrons*	Body feather	BZ	2021	1	0.013	—	—
		Hispaniolan Woodpecker	*Melanerpes striatus*	Whole blood	DR	2017	3	0.006 ± 0.008	0.001–0.015	142.6%
		Hoffmann's Woodpecker	*Melanerpes hoffmannii*	Whole blood	NI	2014	1	0.001	—	—
				Tail feather	CR, NI	2010–2014	2	0.050 ± 0.025	0.033–0.068	49.0%
		Little Woodpecker	*Dryobates passerinus*	Body feather	PE	2019	2	0.111 ± 0.058	0.071–0.152	51.8%

		Rufous-breasted Piculet	*Picumnus rufiventris*	Body feather	PE	2019	5	0.341 ± 0.165	0.115–0.578	48.3%
				Tail feather	PE	2018	1	0.057	—	—
		Smoky-brown Woodpecker	*Dryobates fumigatus*	Whole blood	BZ	2021	1	0.001	—	—
				Body feather	BZ	2021	1	0.008	—	—
		Spot-breasted Woodpecker	*Colaptes punctigula*	Whole blood	CO	2023	1	0.179	—	—
				Body feather	CO, PE	2019–2023	2	6.425 ± 4.662	3.128–9.721	72.6%
		Yucatan Woodpecker	*Melanerpes pygmaeus*	Whole blood	BZ	2021	2	0.009 ± 0.004	0.006–0.013	47.5%
				Body feather	BZ	2021	2	0.145 ± 0.039	0.117–0.172	26.9%
				Tail feather	BZ	2021	2	0.155 ± 0.071	0.105–0.205	45.7%
	Ramphastidae (Toucans)	All species	—	Body feather	PE	2019	10	0.203 ± 0.085	0.069–0.315	42.1%
		Curl-crested Aracari	*Pteroglossus beauharnaisii*	Body feather	PE	2019	2	0.073 ± 0.006	0.069–0.077	7.9%
		Ivory-billed Aracari	*Pteroglossus azara*	Body feather	PE	2019	3	0.237 ± 0.075	0.167–0.315	31.5%
		Lettered Aracari	*Pteroglossus inscriptus*	Body feather	PE	2019	5	0.235 ± 0.055	0.155–0.292	23.3%
Psittaciformes	All families	—	—	Whole blood	DR	2017	1	0.001	—	—
	Psittacidae (New World and African Parrots)	All species	—	Whole blood	DR	2017	1	0.001	—	—
		Hispaniolan Parakeet	*Psittacara chloropterus*	Whole blood	DR	2017	1	0.001	—	—
Strigiformes	All families	—	—	Body feather	PE	2011–2019	5	0.833 ± 1.055	0.092–2.695	126.7%
	Strigidae (Owls)	All species	—	Body feather	PE	2011–2019	5	0.833 ± 1.055	0.092–2.695	126.7%
		Burrowing Owl	*Athene cunicularia*	Body feather	PE	2011	1	2.695	—	—
		Tawny-bellied Screech-Owl	*Megascops watsonii*	Body feather	PE	2019	2	0.253 ± 0.228	0.092–0.415	90.2
		Tropical Screech-Owl	*Megascops choliba*	Body feather	PE	2019	2	0.482 ± 0.123	0.395–0.569	25.5%
Suliformes	All families	—	—	Whole blood	MX	2013	1	0.162	—	—
				Tail feather	MX	2013	2	2.011 ± 1.426	1.003–3.019	70.9%
	Sulidae (Boobies and Gannets)	All species	—	Whole blood	MX	2013	1	0.162	—	—
				Tail feather	MX	2013	2	2.011 ± 1.426	1.003–3.019	70.9%
		Brown Booby	*Sula leucogaster*	Whole blood	MX	2013	1	0.162	—	—
				Tail feather	MX	2013	2	2.011 ± 1.426	1.003–3.019	70.9%
Tinamiformes	All families	—	—	Body feather	PE	2019	1	0.048	—	—
	Tinamidae (Tinamous)	All species	—	Body feather	PE	2019	1	0.048	—	—
		Little Tinamou	*Crypturellus soui*	Body feather	PE	2019	1	0.048	—	—
Trogoniformes	All families	—	—	Body feather	PE	2019	1	0.480	—	—
	Trogonidae	All species	—	Body feather	PE	2019	1	0.480	—	—

(Trogons)									
	Black-tailed Trogon	*Trogon melanurus*	Body feather	PE	2019	1	0.480	—	—

Table S9. Non-lethal tissue sampling procedural and inferential flowchart for Hg analysis: ww = wet weight, dw = dry weight, and fw = fresh weight. An asterisks (*) indicates that the sum does not include the cost of refrigeration, which varies based on machine energy efficiency and geography.

Desired tissue type		Method of collection	Approximate cost per sample (US Dollars)	Storage requirements	Hg Inference	
					Temporal exposure	Geographic exposure
Blood (ww or dw)	Whole blood	Heparinized capillary tube or vial	$1.16*	Standard freezer	Days to weeks	Site-specific
	Dried blood spots	Filter paper	$1.72	Ambient temperature	Days to weeks	Site-specific
Feather (fw)	Secondary feathers (S2)	Clipping	$0.04	Ambient temperature	Duration of feather growth	Varies with movement behavior
	Tail feathers (R6)	Clipping or plucking	$0.04	Ambient temperature	Duration of feather growth	Varies with movement behavior
	Flank feathers	Plucking	$0.04	Ambient temperature	Duration of feather growth	Varies with movement behavior
Egg (ww or dw)		Gentle handling	$0.04*	Standard freezer	Duration of pre-laying egg development	Site-specific

Table S10. Summary of 249 abundant, widespread species with relatively high trophic positions that could be useful sentinels for assessing avian MeHg exposure in various Neotropical habitats. An asterisks (*) indicates that other members within this group may also be useful. Relative abundance was evaluated by visually inspecting mist-netting capture data and country-level eBird Bar Charts (https://ebird.org/GuideMe?cmd=changeLocation). The exclusion of a family or species from this list does not necessarily signify that those taxa would be poor MeHg bioindicators, but that sample acquisition could be challenging. Focal species selection for a given research effort should ultimately be informed by their abundance at the intended sampling locations, their diet and trophic niche, as well as the geographic scale of interest. Species are arranged alphabetically by order and family.

Order	Family	Common name	Latin name	Trophic niche	Migratory status	Primary habitat(s)	Region(s) occupied
**Accipitriformes**	**Accipitridae (Hawks, Eagles, and Kites) ***	Bicolored Hawk	*Accipiter bicolor*	Terrestrial vertivore	Resident	Tropical deciduous forest; tropical lowland evergreen forest	Mexico to Chile
		Black-collared Hawk	*Busarellus nigricollis*	Terrestrial vertivore	Resident	Flooded tropical evergreen forest; freshwater marshes	Mexico to Argentina

		Double-toothed Kite	*Harpagus bidentatus*	Terrestrial vertivore	Resident	Tropical lowland evergreen forest; montane evergreen forest	Mexico to Brazil
		Gray Hawk	*Buteo plagiatus*	Terrestrial vertivore	Partial migrant	Gallery forest; tropical deciduous forest	Arizona, USA to Costa Rica
		Great Black Hawk	*Buteogallus urubitinga*	Terrestrial vertivore	Resident	Tropical deciduous forest; tropical lowland evergreen forest	Mexico to Argentina
		Red-tailed Hawk	*Buteo jamaicensis*	Terrestrial vertivore	Partial migrant	Tropical deciduous forest; pine-oak forest	Canada to Panama; West Indies
		Roadside Hawk	*Rupornis magnirostris*	Terrestrial vertivore	Resident	Tropical lowland evergreen forest; tropical deciduous forest	Mexico to Argentina
		Savanna Hawk	*Buteogallus meridionalis*	Terrestrial vertivore	Resident	Low, seasonally wet grassland; Campo grasslands	Costa Rica to Argentina
		Sharp-shinned Hawk	*Accipiter striatus*	Terrestrial vertivore	Partial migrant	Montane evergreen forest; pine-oak forest; southern temperate forest	Canada to Uruguay; West Indies
		Slate-colored Hawk	*Buteogallus schistaceus*	Terrestrial vertivore	Resident	Flooded tropical evergreen forest	Ecuador to Brazil
		Swallow-tailed Kite	*Elanoides forficatus*	Invertivore	Partial migrant	Tropical lowland evergreen forest; tropical deciduous forest	South Carolina, USA to Brazil; West Indies
		Variable Hawk	*Geranoaetus polyosoma*	Terrestrial vertivore	Resident	Arid lowland/montane scrub; Campo grasslands	Colombia to Chile
		White-tailed Kite	*Elanus leucurus*	Terrestrial vertivore	Resident	Pastures/agricultural lands; second-growth scrub	California, USA to Argentina
	**Pandionidae (Osprey)**	Osprey	*Pandion haliaetus*	Aquatic predator	Partial migrant	Coastal waters; freshwater lakes and ponds; rivers	Worldwide
**Caprimulgiformes**	**Caprimulgidae (Nightjars and Allies) ***	Common Pauraque	*Nyctidromus albicollis*	Invertivore	Resident	Tropical lowland evergreen forest; secondary forest; tropical deciduous forest	Texas, USA to Argentina
	**Trochilidae (Hummingbirds) ***	Black-throated Mango	*Anthracothorax nigricollis*	Nectarivore	Resident	Secondary forest; gallery forest	Panama to Uruguay
		Collared Inca	*Coeligena torquata*	Nectarivore	Resident	Montane evergreen forest	Venezuela to Bolivia
		Fork-tailed Woodnymph	*Thalurania furcata*	Nectarivore	Resident	Tropical lowland evergreen forest; secondary forest	Venezuela to Argentina
		Glittering-throated Emerald	*Amazilia fimbriata*	Nectarivore	Resident	River-edge forest; gallery forest	Venezuela to Uruguay
		Rufous-breasted Hermit	*Glaucis hirsutus*	Nectarivore	Resident	Tropical lowland evergreen forest; secondary forest	Panama to Brazil
		Rufous-tailed Hummingbird	*Amazilia tzacatl*	Nectarivore	Resident	Tropical lowland evergreen forest; secondary forest	Mexico to Ecuador
		Sparkling Violetear	*Colibri coruscans*	Nectarivore	Resident	Secondary forest; second-growth scrub	Venezuela to Chile
		White-necked Jacobin	*Florisuga mellivora*	Nectarivore	Resident	Tropical lowland evergreen forest; secondary forest	Mexico to Bolivia

**Cathartiformes**	**Cathartidae (New World Vultures)**	Black Vulture	*Coragyps atratus*	Scavenger	Resident	Second-growth scrub; low, seasonally wet grassland	Connecticut, USA to Chile
**Charadriiformes ***	**Charadriidae (Plovers and Lapwings) ***	Black-bellied Plover	*Pluvialis squatarola*	Aquatic predator	Full migrant	Coastal sand beaches/mudflats; coastal rocky beaches	Worldwide
		Collared Plover	*Charadrius collaris*	Aquatic predator	Resident	Riverine sand beaches; coastal sand beaches/mudflats	Mexico to Argentina
		Southern Lapwing	*Vanellus chilensis*	Omnivore	Resident	Low, seasonally wet grassland; pastures/agricultural lands	Mexico to Chile
	**Haematopodidae (Oystercatchers) ***	American Oystercatcher	*Haematopus palliatus*	Aquatic predator	Partial migrant	Coastal sand beaches/mudflats; coastal rocky beaches	United States to Argentina
	**Jacanidae (Jacanas)**	Northern Jacana	*Jacana spinosa*	Aquatic predator	Resident	Freshwater marshes	West Indies; Mexico to Panama
		Wattled Jacana	*Jacana jacana*	Omnivore	Resident	Freshwater marshes	Panama to Argentina
	**Laridae (Gulls, Terns, and Skimmers) ***	Black Skimmer	*Rynchops niger*	Aquatic predator	Partial migrant	Freshwater/saltwater /brackish marshes	United States to Chile
		Black Tern	*Chlidonias niger*	Aquatic predator	Full migrant	Freshwater/saltwater /brackish marshes; freshwater lakes and ponds	Worldwide
		Large-billed Tern	*Phaetusa simplex*	Aquatic predator	Resident	Rivers; freshwater lakes and ponds	Colombia to Argentina
		Royal Tern	*Thalasseus maximus*	Aquatic predator	Partial migrant	Coastal waters	United States to Argentina; Western Africa
		Sandwich Tern	*Thalasseus sandvicensis*	Aquatic predator	Partial migrant	Coastal waters	Worldwide
	**Recurvirostridae (Stilts and Avocets)**	Black-necked Stilt	*Himantopus mexicanus*	Aquatic predator	Partial migrant	Freshwater/saltwater /brackish marshes; coastal sand beaches/mudflats	United States to Argentina; West Indies
	**Scolopacidae (Sandpipers and Allies) ***	Greater Yellowlegs	*Tringa melanoleuca*	Aquatic predator	Full migrant	Freshwater/saltwater /brackish marshes; coastal sand beaches/mudflats	Canada to Chile; West Indies
		Least Sandpiper	*Calidris minutilla*	Aquatic predator	Full migrant	Freshwater/saltwater /brackish marshes	Canada to Peru; West Indies
		Ruddy Turnstone	*Arenaria interpres*	Aquatic predator	Full migrant	Coastal sand beaches/mudflats; coastal rocky beaches	Worldwide
		Spotted Sandpiper	*Actitis macularius*	Aquatic predator	Full migrant	Riverine sand beaches; freshwater lakes and ponds	Canada to Argentina; West Indies
**Ciconiiformes ***	**Ciconiidae (Storks) ***	Wood Stork	*Mycteria americana*	Aquatic predator	Partial migrant	Freshwater/saltwater /brackish marshes; freshwater lakes and ponds	United States to Argentina; West Indies
**Coraciiformes ***	**Alcedinidae (Kingfishers) ***	Amazon Kingfisher	*Chloroceryle amazona*	Aquatic predator	Resident	Rivers; freshwater lakes and ponds	Mexico to Argentina
		American Pygmy Kingfisher	*Chloroceryle aenea*	Aquatic predator	Resident	Streams; freshwater lakes and ponds	Mexico to Paraguay
		Belted Kingfisher	*Megaceryle alcyon*	Aquatic predator	Full migrant	Rivers; freshwater lakes and ponds	Canada to Colombia; West Indies

		Green-and-rufous Kingfisher	*Chloroceryle inda*	Aquatic predator	Resident	Streams; freshwater lakes and ponds	Nicaragua to Brazil
		Green Kingfisher	*Chloroceryle americana*	Aquatic predator	Resident	Streams; freshwater lakes and ponds	Texas, USA to Argentina
		Ringed Kingfisher	*Megaceryle torquata*	Aquatic predator	Resident	Rivers; freshwater lakes and ponds	Texas, USA to Chile
	**Momotidae (Motmots) ***	Amazonian Motmot	*Momotus momota*	Omnivore	Resident	Tropical lowland evergreen forest; montane evergreen forest; secondary forest	Venezuela to Argentina
		Andean Motmot	*Momotus aequatorialis*	Invertivore	Resident	Montane evergreen forest; secondary forest	Colombia to Bolivia
		Broad-billed Motmot	*Electron platyrhynchum*	Invertivore	Resident	Tropical lowland evergreen forest	Honduras to Bolivia
		Lesson's Motmot	*Momotus lessonii*	Omnivore	Resident	Tropical lowland evergreen forest; montane evergreen forest; secondary forest	Mexico to Panama
		Rufous Motmot	*Baryphthengus martii*	Omnivore	Resident	Tropical lowland evergreen forest	Honduras to Bolivia
**Cuculiformes ***	**Cuculidae (Cuckoos) ***	Greater Ani	*Crotophaga major*	Omnivore	Resident	Flooded tropical evergreen forest; river-edge forest	Panama to Uruguay
		Groove-billed Ani	*Crotophaga sulcirostris*	Invertivore	Resident	Second-growth scrub; riparian thickets	Texas, USA to Peru
		Mangrove Cuckoo	*Coccyzus minor*	Invertivore	Resident	Tropical deciduous forest; gallery forest; mangrove forest	West Indies; Mexico to Panama; Guiana Shield
		Smooth-billed Ani	*Crotophaga ani*	Omnivore	Resident	Second-growth scrub; river island scrub	Florida, USA; West Indies; Costa Rica to Uruguay
		Squirrel Cuckoo	*Piaya cayana*	Invertivore	Resident	Tropical lowland evergreen forest; tropical deciduous forest; secondary forest	Mexico to Uruguay
**Falconiformes ***	**Falconidae (Falcons and Caracaras) ***	American Kestrel	*Falco sparverius*	Omnivore	Partial migrant	Temperate grassland; arid lowland scrub; pastures/agricultural lands	Canada to Argentina; West Indies
		Bat Falcon	*Falco rufigularis*	Terrestrial vertivore	Resident	Tropical lowland evergreen forest; tropical deciduous forest	Mexico to Paraguay
		Barred Forest-Falcon	*Micrastur ruficollis*	Terrestrial vertivore	Resident	Tropical lowland evergreen forest; montane evergreen forest	Mexico to Brazil
		Collared Forest-Falcon	*Micrastur semitorquatus*	Terrestrial vertivore	Resident	Tropical lowland evergreen forest; tropical deciduous forest	Mexico to Brazil
		Crested Caracara	*Caracara plancus*	Omnivore	Resident	Arid lowland/montane scrub; low, seasonally wet grassland	Texas, USA to Chile; Cuba
		Laughing Falcon	*Herpetotheres cachinnans*	Terrestrial vertivore	Resident	Tropical lowland evergreen forest; river-edge forest; tropical deciduous forest	Mexico to Argentina

		Peregrine Falcon	*Falco peregrinus*	Terrestrial vertivore	Partial migrant	Coastal waters; arid montane scrub; tropical deciduous forest	Worldwide
		Yellow-headed Caracara	*Milvago chimachima*	Omnivore	Resident	Low, seasonally wet grassland; river island scrub; second-growth scrub	Nicaragua to Uruguay
**Galbuliformes**	**Galbulidae (Jacamars) ***	Bluish-fronted Jacamar	*Galbula cyanescens*	Invertivore	Resident	Tropical lowland evergreen forest	Peru to Brazil
		Rufous-tailed Jacamar	*Galbula ruficauda*	Invertivore	Resident	Tropical lowland evergreen forest; gallery forest	Mexico to Brazil
**Passeriformes**	**Cardinalidae (Cardinals and Allies) ***	Flame-colored Tanager	*Piranga bidentata*	Invertivore	Resident	Montane evergreen forest; pine-oak forest	Arizona, USA to Panama
		Hepatic Tanager	*Piranga flava*	Invertivore	Resident	Pine-oak forest; gallery forest; tropical deciduous forest	Colorado, USA to Argentina
		Red-crowned Ant-Tanager	*Habia rubica*	Invertivore	Resident	Tropical lowland evergreen forest	Mexico to Brazil
		Red-throated Ant-Tanager	*Habia fuscicauda*	Invertivore	Resident	Tropical lowland evergreen forest; flooded tropical evergreen forest	Mexico to Colombia
		Summer Tanager	*Piranga rubra*	Invertivore	Partial migrant	Gallery forest	Iowa, USA to Bolivia; West Indies
		Western Tanager	*Piranga ludoviciana*	Invertivore	Full migrant	Pine-oak forest	Canada to Panama
	**Cinclidae (Dippers) ***	American Dipper	*Cinclus mexicanus*	Aquatic predator	Resident	Streams; rivers	Alaska, USA to Panama
		White-capped Dipper	*Cinclus leucocephalus*	Aquatic predator	Resident	Streams; rivers	Colombia to Bolivia
	**Donacobiidae (Donacobius)**	Black-capped Donacobius	*Donacobius atricapilla*	Invertivore	Resident	Freshwater marshes	Colombia to Argentina
	**Formicariidae (Antthrushes) ***	Black-faced Antthrush	*Formicarius analis*	Invertivore	Resident	Tropical lowland evergreen forest; flooded tropical evergreen forest	Mexico to Bolivia
	**Furnariidae (Ovenbirds and Woodcreepers) ***	Azara's Spinetail	*Synallaxis azarae*	Invertivore	Resident	Montane evergreen forest; secondary forest	Venezuela to Argentina
		Buff-throated Foliage-gleaner	*Automolus ochrolaemus*	Invertivore	Resident	Tropical lowland evergreen forest	Mexico to Bolivia
		Buff-throated Woodcreeper	*Xiphorhynchus guttatus*	Invertivore	Resident	Tropical lowland evergreen forest; flooded tropical evergreen forest; secondary forest	Venezuela to Bolivia
		Cream-winged Cinclodes	*Cinclodes albiventris*	Invertivore	Resident	Paramo grassland; arid montane scrub	Peru to Argentina
		Cocoa Woodcreeper	*Xiphorhynchus susurrans*	Invertivore	Resident	Tropical lowland evergreen forest; gallery forest; tropical deciduous forest	Guatemala to Venezuela
		Common Miner	*Geositta cunicularia*	Invertivore	Resident	Southern temperate grassland; puna grassland	Peru to Chile
		Ivory-billed Woodcreeper	*Xiphorhynchus flavigaster*	Invertivore	Resident	Tropical lowland evergreen forest; montane evergreen forest; tropical deciduous forest	Mexico to Costa Rica

Many-striped Canastero	*Asthenes flammulata*	Invertivore	Resident	Paramo grassland	Columbia to Peru
Montane Foliage-gleaner	*Anabacerthia striaticollis*	Invertivore	Resident	Montane evergreen forest	Venezuela to Bolivia
Montane Woodcreeper	*Lepidocolaptes lacrymiger*	Invertivore	Resident	Montane evergreen forest	Venezuela to Bolivia
Olivaceous Woodcreeper	*Sittasomus griseicapillus*	Invertivore	Resident	Tropical lowland evergreen forest; montane evergreen forest; tropical deciduous forest	Mexico to Uruguay
Pale-breasted Spinetail	*Synallaxis albescens*	Invertivore	Resident	Cerrado; campo grasslands; pastures/agricultural lands	Costa Rica to Argentina
Pale-legged Hornero	*Furnarius leucopus*	Invertivore	Resident	Second-growth scrub; Pastures/agricultural lands	Colombia to Paraguay
Pearled Treerunner	*Margarornis squamiger*	Invertivore	Resident	Montane evergreen forest; elfin forest	Venezuela to Bolivia
Plain-brown Woodcreeper	*Dendrocincla fuliginosa*	Invertivore	Resident	Tropical lowland evergreen forest	Honduras to Bolivia
Plain-crowned Spinetail	*Synallaxis gujanensis*	Invertivore	Resident	River-edge forest; secondary forest	Venezuela to Bolivia
Plain Xenops	*Xenops minutus*	Invertivore	Resident	Tropical lowland evergreen forest; flooded tropical evergreen forest	Mexico to Brazil
Red-faced Spinetail	*Cranioleuca erythrops*	Invertivore	Resident	Montane evergreen forest	Costa Rica to Ecuador
Ruddy Woodcreeper	*Dendrocincla homochroa*	Invertivore	Resident	Tropical lowland evergreen forest; montane evergreen forest; tropical deciduous forest	Mexico to Venezuela
Slaty Spinetail	*Synallaxis brachyura*	Invertivore	Resident	Second-growth scrub; riparian thickets	Honduras to Ecuador
Spot-crowned Woodcreeper	*Lepidocolaptes affinis*	Invertivore	Resident	Montane evergreen forest; pine-oak forest	Mexico to Panama
Spotted Barbtail	*Premnoplex brunnescens*	Invertivore	Resident	Montane evergreen forest	Costa Rica to Bolivia
Spotted Woodcreeper	*Xiphorhynchus erythropygius*	Invertivore	Resident	Montane evergreen forest; tropical lowland evergreen forest	Mexico to Ecuador
Straight-billed Woodcreeper	*Dendroplex picus*	Invertivore	Resident	River-edge forest; tropical deciduous forest	Panama to Brazil
Streaked Xenops	*Xenops rutilans*	Invertivore	Resident	Tropical lowland evergreen forest; montane evergreen forest	Costa Rica to Brazil
Streak-headed Woodcreeper	*Lepidocolaptes souleyetii*	Invertivore	Resident	Tropical deciduous forest; gallery forest; tropical lowland evergreen forest	Mexico to Peru
Tawny-winged Woodcreeper	*Dendrocincla anabatina*	Invertivore	Resident	Tropical lowland evergreen forest	Mexico to Panama
Wedge-billed Woodcreeper	*Glyphorynchus spirurus*	Invertivore	Resident	Tropical lowland evergreen forest; montane evergreen forest	Mexico to Brazil

	Wren-like Rushbird	*Phleocryptes melanops*	Invertivore	Resident	Freshwater marshes	Peru to Chile
	Yellow-chinned Spinetail	*Certhiaxis cinnamomeus*	Invertivore	Resident	Freshwater marshes; mangrove forests	Colombia to Brazil
**Hirundinidae (Swallows) ***	Barn Swallow	*Hirundo rustica*	Invertivore	Partial migrant	Pastures/agricultural lands; Northern temperate grassland	Worldwide
	Blue-and-white Swallow	*Pygochelidon cyanoleuca*	Invertivore	Austral migrant	Second-growth scrub; pastures/agricultural lands	Costa Rica to Chile
	Gray-breasted Martin	*Progne chalybea*	Invertivore	Partial migrant	Second-growth scrub; pastures/agricultural lands	Mexico to Argentina
	Northern Rough-winged Swallow	*Stelgidopteryx serripennis*	Invertivore	Partial migrant	Second-growth scrub; pastures/agricultural lands	Canada to Panama; West Indies
	Southern Rough-winged Swallow	*Stelgidopteryx ruficollis*	Invertivore	Resident	Second-growth scrub; pastures/agricultural lands	Nicaragua to Argentina
**Oxyruncidae (Sharpbill, Royal Flycatcher, and Allies) ***	Royal Flycatcher	*Onychorhynchus coronatus*	Invertivore	Resident	Tropical lowland evergreen forest	Mexico to Brazil
	Ruddy-tailed Flycatcher	*Terenotriccus erythrurus*	Invertivore	Resident	Tropical lowland evergreen forest	Mexico to Bolivia
**Parulidae (New World Warblers) ***	American Redstart	*Setophaga ruticilla*	Invertivore	Full migrant	Tropical lowland evergreen forest; montane evergreen forest	Canada to Peru; West Indies
	Black-and-white Warbler	*Mniotilta varia*	Invertivore	Full migrant	Tropical lowland evergreen forest; montane evergreen forest	Canada to Ecuador; West Indies
	Buff-rumped Warbler	*Myiothlypis fulvicauda*	Invertivore	Resident	Tropical lowland evergreen forest	Honduras to Peru
	Common Yellowthroat	*Geothlypis trichas*	Invertivore	Partial migrant	Freshwater/saltwater /brackish marshes	Canada to Panama; West Indies
	Golden-crowned Warbler	*Basileuterus culicivorus*	Invertivore	Resident	Tropical lowland evergreen forest; montane evergreen forest	Mexico to Argentina
	Gray-crowned Yellowthroat	*Geothlypis poliocephala*	Invertivore	Resident	Second-growth scrub; riparian thickets	Mexico to Panama
	Hooded Warbler	*Setophaga citrina*	Invertivore	Full migrant	Tropical lowland evergreen forest; secondary forest	Canada to Venezuela; West Indies
	Masked Yellowthroat	*Geothlypis aequinoctialis*	Invertivore	Resident	Riparian thickets; freshwater marshes	Venezuela to Argentina
	Northern Waterthrush	*Parkesia noveboracensis*	Invertivore	Full migrant	Tropical lowland evergreen forest; secondary forest	Alaska, USA to Ecuador; West Indies
	Rufous-capped Warbler	*Basileuterus rufifrons*	Invertivore	Resident	Tropical deciduous forest; tropical lowland evergreen forest	Mexico to Colombia
	Slate-throated Redstart	*Myioborus miniatus*	Invertivore	Resident	Montane evergreen forest; secondary forest	Mexico to Bolivia
	Tropical Parula	*Setophaga pitiayumi*	Invertivore	Resident	Montane evergreen forest; tropical lowland evergreen forest; tropical deciduous forest	Texas, USA to Argentina

	Yellow-rumped Warbler	*Setophaga coronata*	Invertivore	Full migrant	Pine forest; pine-oak forest	Alaska, USA to Colombia; West Indies
	Yellow Warbler	*Setophaga petechia*	Invertivore	Partial migrant	Gallery forest; secondary forest; mangrove forest	Alaska, USA to Peru; West Indies
**Passerellidae (New World Sparrows) ***	Black-striped Sparrow	*Arremonops conirostris*	Omnivore	Resident	Tropical lowland evergreen forest; tropical deciduous forest	Honduras to Ecuador
	Chestnut-capped Brushfinch	*Arremon brunneinucha*	Invertivore	Resident	Montane evergreen forest; pine-oak forest	Mexico to Peru
	Common Chlorospingus	*Chlorospingus flavopectus*	Invertivore	Resident	Montane evergreen forest; secondary forest	Mexico to Argentina
	Orange-billed Sparrow	*Arremon aurantiirostris*	Omnivore	Resident	Tropical lowland evergreen forest	Mexico to Peru
	Pectoral Sparrow	*Arremon taciturnus*	Omnivore	Resident	Tropical lowland evergreen forest	Venezuela to Brazil
	Rufous-collared Sparrow	*Zonotrichia capensis*	Omnivore	Resident	Arid lowland/montane scrub; second-growth scrub	Mexico to Chile; West Indies
	Yellow-browed Sparrow	*Ammodramus aurifrons*	Granivore	Resident	River island scrub; second-growth scrub	Venezuela to Bolivia
**Polioptilidae (Gnatcatchers) ***	Blue-gray Gnatcatcher	*Polioptila caerulea*	Invertivore	Partial migrant	Tropical deciduous forest; tropical lowland evergreen forest	Canada to Honduras; West Indies
	Tropical Gnatcatcher	*Polioptila plumbea*	Invertivore	Resident	Tropical deciduous forest; tropical lowland evergreen forest; Arid lowland/montane scrub	Mexico to Brazil
**Thamnophilidae (Typical Antbirds) ***	Barred Antshrike	*Thamnophilus doliatus*	Invertivore	Resident	Second-growth scrub; riparian thickets; river island scrub	Mexico to Argentina
	Black-faced Antbird	*Myrmoborus myotherinus*	Invertivore	Resident	Tropical lowland evergreen forest	Ecuador to Brazil
	Black-spotted Bare-eye	*Phlegopsis nigromaculata*	Invertivore	Resident	Tropical lowland evergreen forest; flooded tropical evergreen forest	Ecuador to Brazil
	Common Scale-backed Antbird	*Willisornis poecilinotus*	Invertivore	Resident	Tropical lowland evergreen forest	Venezuela to Bolivia
	Chestnut-backed Antbird	*Poliocrania exsul*	Invertivore	Resident	Tropical lowland evergreen forest; secondary forest	Honduras to Ecuador
	Dot-winged Antwren	*Microrhopias quixensis*	Invertivore	Resident	Tropical lowland evergreen forest	Mexico to Brazil
	Dusky Antbird	*Cercomacroides tyrannina*	Invertivore	Resident	Tropical lowland evergreen forest; secondary forest	Mexico to Brazil
	Dusky-throated Antshrike	*Thamnomanes ardesiacus*	Invertivore	Resident	Tropical lowland evergreen forest	Ecuador to Brazil
	Great Antshrike	*Taraba major*	Invertivore	Resident	Tropical lowland evergreen forest; secondary forest; riparian thickets	Mexico to Argentina
	Long-winged Antwren	*Myrmotherula longipennis*	Invertivore	Resident	Tropical lowland evergreen forest	Ecuador to Brazil
	Plain Antvireo	*Dysithamnus mentalis*	Invertivore	Resident	Montane evergreen forest; tropical	Mexico to Brazil

					lowland evergreen forest	
	Plain-throated Antwren	*Isleria hauxwelli*	Invertivore	Resident	Tropical lowland evergreen forest	Ecuador to Brazil
	Plain-winged Antshrike	Thamnophilus schistaceus	Invertivore	Resident	Tropical lowland evergreen forest; flooded tropical evergreen forest	Colombia to Bolivia
	Pygmy Antwren	*Myrmotherula brachyura*	Invertivore	Resident	Tropical lowland evergreen forest; flooded tropical evergreen forest; secondary forest	Venezuela to Bolivia
	Slaty Antwren	*Myrmotherula schisticolor*	Invertivore	Resident	Montane evergreen forest; tropical lowland evergreen forest	Mexico to Peru
	Spotted Antbird	*Hylophylax naevioides*	Invertivore	Resident	Tropical lowland evergreen forest	Honduras to Ecuador
	White-browed Antbird	*Myrmoborus leucophrys*	Invertivore	Resident	Tropical lowland evergreen forest; montane evergreen forest; river-edge forest	Venezuela to Bolivia
	White-flanked Antwren	*Myrmotherula axillaris*	Invertivore	Resident	Tropical lowland evergreen forest; flooded tropical evergreen forest	Honduras to Brazil
	White-fringed Antwren	*Formicivora grisea*	Invertivore	Resident	Tropical deciduous forest; secondary forest; gallery forest	Panama to Brazil
**Thraupidae (Tanagers and Allies) ***	Blue-gray Tanager	*Thraupis episcopus*	Omnivore	Resident	Tropical lowland evergreen forest; secondary forest	Mexico to Bolivia
	Blue-winged Mountain Tanager	*Anisognathus somptuosus*	Invertivore	Resident	Montane evergreen forest	Venezuela to Bolivia
	Gray-headed Tanager	*Eucometis penicillata*	Invertivore	Resident	Tropical lowland evergreen forest; gallery forest; secondary forest	Mexico to Paraguay
	Hooded Mountain Tanager	*Buthraupis montana*	Invertivore	Resident	Montane evergreen forest	Venezuela to Bolivia
	Masked Flowerpiercer	*Diglossa cyanea*	Frugivore	Resident	Montane evergreen forest; elfin forest	Venezuela to Bolivia
	Palm Tanager	*Thraupis palmarum*	Omnivore	Resident	Tropical lowland evergreen forest; secondary forest	Nicaragua to Brazil
	Red-legged Honeycreeper	*Cyanerpes cyaneus*	Omnivore	Resident	Tropical lowland evergreen forest; secondary forest	Mexico to Brazil; West Indies
	Scarlet-bellied Mountain Tanager	*Anisognathus igniventris*	Frugivore	Resident	Montane evergreen forest; elfin forest	Venezuela to Bolivia
	Silver-beaked Tanager	*Ramphocelus carbo*	Omnivore	Resident	Secondary forest; tropical lowland evergreen forest	Venezuela to Paraguay
	White-lined Tanager	*Tachyphonus rufus*	Invertivore	Resident	Secondary forest; gallery forest	Nicaragua to Argentina
**Troglodytidae (Wrens) ***	Buff-breasted Wren	*Cantorchilus leucotis*	Invertivore	Resident	Tropical lowland evergreen forest; gallery forest	Panama to Paraguay
	Coraya Wren	*Pheugopedius coraya*	Invertivore	Resident	Tropical lowland evergreen forest; montane evergreen forest	Ecuador to Brazil

	Gray-breasted Wood-Wren	*Henicorhina leucophrys*	Invertivore	Resident	Montane evergreen forest	Mexico to Bolivia
	House Wren	*Troglodytes aedon*	Invertivore	Resident	Second-growth scrub; arid lowland/montane scrub	Canada to Chile
	Moustached Wren	*Pheugopedius genibarbis*	Invertivore	Resident	Tropical lowland evergreen forest; montane evergreen forest	Peru to Brazil
	Musician Wren	*Cyphorhinus arada*	Invertivore	Resident	Tropical lowland evergreen forest	Ecuador to Brazil
	Thrush-like Wren	*Campylorhynchus turdinus*	Invertivore	Resident	Tropical lowland evergreen forest; secondary forest	Colombia to Paraguay
	Scaly-breasted Wren	*Microcerculus marginatus*	Invertivore	Resident	Tropical lowland evergreen forest	Costa Rica to Bolivia
	White-breasted Wood-Wren	*Henicorhina leucosticta*	Invertivore	Resident	Tropical lowland evergreen forest; montane evergreen forest	Mexico to Peru
**Turdidae (Thrushes and Allies) ***	Andean Solitaire	*Myadestes ralloides*	Invertivore	Resident	Montane evergreen forest; secondary forest	Colombia to Bolivia
	Black-billed Thrush	*Turdus ignobilis*	Omnivore	Resident	River-edge forest; gallery forest	Colombia to Bolivia
	Chiguanco Thrush	*Turdus chiguanco*	Invertivore	Resident	Secondary forest; semihumid/humid/arid montane scrub	Ecuador to Argentina
	Clay-colored Thrush	*Turdus grayi*	Omnivore	Resident	Tropical lowland evergreen forest; tropical deciduous forest; secondary forest	Texas, USA to Colombia
	Great Thrush	*Turdus fuscater*	Invertivore	Resident	Montane evergreen forest; secondary forest	Colombia to Bolivia
	Hauxwell's Thrush	*Turdus hauxwelli*	Invertivore	Resident	Flooded tropical evergreen forest; river-edge forest	Colombia to Bolivia
	Red-legged Thrush	*Turdus plumbeus*	Omnivore	Resident	Tropical deciduous forest; tropical lowland evergreen forest; montane evergreen forest	West Indies
	Rufous-bellied Thrush	*Turdus rufiventris*	Invertivore	Resident	Tropical lowland evergreen forest; montane evergreen forest	Brazil to Argentina
	Swainson's Thrush	*Catharus ustulatus*	Invertivore	Full migrant	Montane evergreen forest; tropical lowland evergreen forest	Alaska, USA to Argentina
	White-necked Thrush	*Turdus albicollis*	Invertivore	Resident	Tropical lowland evergreen forest	Colombia to Uruguay
	Wood Thrush	*Hylocichla mustelina*	Invertivore	Full migrant	Tropical lowland evergreen forest; secondary forest	Canada to Colombia
**Tyrannidae (Tyrant Flycatchers) ***	Black Phoebe	*Sayornis nigricans*	Invertivore	Resident	Riparian thickets; streams	Oregon, USA to Argentina
	Black-billed Shrike-Tyrant	*Agriornis montanus*	Omnivore	Resident	Arid montane scrub; puna grassland	Colombia to Chile
	Boat-billed Flycatcher	*Megarynchus pitangua*	Invertivore	Resident	Tropical lowland evergreen forest;	Mexico to Argentina

				secondary forest	
Bran-colored Flycatcher	*Myiophobus fasciatus*	Invertivore	Resident	Second-growth scrub; riparian thickets	Costa Rica to Argentina
Bright-rumped Attila	*Attila spadiceus*	Invertivore	Resident	Tropical lowland evergreen forest; secondary forest; montane evergreen forest	Mexico to Bolivia
Brown-backed Chat-Tyrant	*Ochthoeca fumicolor*	Invertivore	Resident	Paramo grassland; second-growth scrub	Venezuela to Bolivia
Cattle Tyrant	*Machetornis rixosa*	Invertivore	Resident	Pastures/agricultural lands; second-growth scrub	Panama to Argentina
Cinnamon Flycatcher	*Pyrrhomyias cinnamomeus*	Invertivore	Resident	Montane evergreen forest	Venezuela to Argentina
Common Tody-Flycatcher	*Todirostrum cinereum*	Invertivore	Resident	Tropical lowland evergreen forest; montane evergreen forest; tropical deciduous forest	Mexico to Brazil
Drab Water Tyrant	*Ochthornis littoralis*	Invertivore	Resident	Riverine sand beaches	Ecuador to Brazil
Dusky-capped Flycatcher	*Myiarchus tuberculifer*	Invertivore	Resident	Montane evergreen forest; tropical lowland evergreen forest; tropical deciduous forest	Arizona, USA to Argentina
Eastern Wood-Pewee	*Contopus virens*	Invertivore	Full migrant	Tropical lowland evergreen forest; secondary forest	Canada to Peru; West Indies
Forest Elaenia	*Myiopagis gaimardii*	Invertivore	Resident	Tropical lowland evergreen forest; secondary forest	Panama to Brazil
Golden-crowned Spadebill	*Platyrinchus coronatus*	Invertivore	Resident	Tropical lowland evergreen forest	Honduras to Bolivia
Great Kiskadee	*Pitangus sulphuratus*	Omnivore	Resident	Secondary forest; riparian thickets	Texas, USA to Argentina
Loggerhead Kingbird	*Tyrannus caudifasciatus*	Invertivore	Resident	Tropical lowland evergreen forest; pine forest	West Indies
Northern Beardless-Tyrannulet	*Camptostoma imberbe*	Invertivore	Resident	Gallery forest; secondary forest	Arizona, USA to Costa Rica
Olive-sided Flycatcher	*Contopus cooperi*	Invertivore	Full migrant	Montane evergreen forest; tropical lowland evergreen forest	Alaska, USA to Bolivia
Rufous-breasted Chat-Tyrant	*Ochthoeca rufipectoralis*	Invertivore	Resident	Montane evergreen forest; elfin forest	Venezuela to Bolivia
Scale-crested Pygmy-Tyrant	*Lophotriccus pileatus*	Invertivore	Resident	Montane evergreen forest; tropical lowland evergreen forest	Honduras to Peru
Sepia-capped Flycatcher	*Leptopogon amaurocephalus*	Invertivore	Resident	Tropical lowland evergreen forest; secondary forest	Mexico to Brazil
Slaty-capped Flycatcher	*Leptopogon superciliaris*	Invertivore	Resident	Montane evergreen forest	Costa Rica to Bolivia
Smoke-colored Pewee	*Contopus fumigatus*	Invertivore	Resident	Montane evergreen forest; secondary forest	Venezuela to Argentina
Social Flycatcher	*Myiozetetes similis*	Invertivore	Resident	Tropical lowland evergreen forest; tropical deciduous forest	Mexico to Argentina

	Southern Beardless-Tyrannulet	*Camptostoma obsoletum*	Invertivore	Resident	Secondary forest; gallery forest	Costa Rica to Uruguay
	Spotted Tody-Flycatcher	*Todirostrum maculatum*	Invertivore	Resident	River-edge forest; river-island scrub; secondary forest	Venezuela to Brazil
	Streak-throated Bush-Tyrant	*Myiotheretes striaticollis*	Invertivore	Resident	Montane evergreen forest; secondary forest	Venezuela to Argentina
	Stub-tailed Spadebill	*Platyrinchus cancrominus*	Invertivore	Resident	Tropical lowland evergreen forest; tropical deciduous forest	Mexico to Panama
	Torrent Tyrannulet	*Serpophaga cinerea*	Invertivore	Resident	Rivers; streams	Costa Rica to Bolivia
	Tropical Kingbird	*Tyrannus melancholicus*	Invertivore	Resident	Secondary forest; gallery forest; river-edge forest	Arizona, USA to Argentina
	Tropical Pewee	*Contopus cinereus*	Invertivore	Resident	Tropical lowland evergreen forest; montane evergreen forest	Mexico to Argentina
	Western Wood-Pewee	*Contopus sordidulus*	Invertivore	Partial migrant	Pine-oak forest; gallery forest	Alaska, USA to Bolivia
	White-crested Elaenia	*Elaenia albiceps*	Invertivore	Austral migrant	Southern temperate forest; montane evergreen forest	Colombia to Chile
	White-throated Spadebil	*Platyrinchus mystaceus*	Invertivore	Resident	Tropical lowland evergreen forest; montane evergreen forest; tropical deciduous forest	Costa Rica to Brazil
	White-throated Tyrannulet	*Mecocerculus leucophrys*	Invertivore	Resident	Elfin forest; montane evergreen forest	Venezuela to Argentina
	Yellow-bellied Elaenia	*Elaenia flavogaster*	Invertivore	Resident	Second-growth scrub; riparian thickets	Mexico to Brazil
	Yellow-breasted Flycatcher	*Tolmomyias flaviventris*	Invertivore	Resident	Tropical lowland evergreen forest; river-edge forest	Panama to Brazil
	Yellow-browed Tody-Flycatcher	*Todirostrum chrysocrotaphum*	Invertivore	Resident	Tropical lowland evergreen forest; flooded tropical evergreen forest; secondary forest	Ecuador to Brazil
	Yellow-crowned Tyrannulet	*Tyrannulus elatus*	Invertivore	Resident	River-edge forest; flooded tropical evergreen forest	Costa Rica to Bolivia
	Yellow-olive Flycatcher	*Tolmomyias sulphurescens*	Invertivore	Resident	Tropical lowland evergreen forest; montane evergreen forest; tropical deciduous forest	Mexico to Uruguay
**Vireonidae (Vireos, Shrike-Babblers, and Erpornis) ***	Brown-capped Vireo	*Vireo leucophrys*	Invertivore	Resident	Montane evergreen forest; secondary forest	Mexico to Bolivia
	Chivi Vireo	*Vireo chivi*	Invertivore	Partial migrant	Tropical lowland evergreen forest; tropical deciduous forest; secondary forest	Venezuela to Argentina
	Lesser Greenlet	*Pachysylvia decurtata*	Invertivore	Resident	Tropical lowland evergreen forest; tropical deciduous forest	Mexico to Ecuador
	Mangrove Vireo	*Vireo pallens*	Invertivore	Resident	Tropical deciduous forest; mangrove forest	Mexico to Costa Rica

		Red-eyed Vireo	*Vireo olivaceus*	Invertivore	Full migrant	Tropical lowland evergreen forest; secondary forest	Canada to Bolivia
		Rufous-browed Peppershrike	*Cyclarhis gujanensis*	Invertivore	Resident	Tropical lowland evergreen forest; gallery forest	Mexico to Argentina
		Tawny-crowned Greenlet	*Tunchiornis ochraceiceps*	Invertivore	Resident	Tropical lowland evergreen forest	Mexico to Bolivia
**Pelecaniformes**	**Ardeidae (Herons, Egrets, and Bitterns) ***	Black-crowned Night-Heron	*Nycticorax nycticorax*	Aquatic predator	Partial migrant	Freshwater/saltwater /brackish marshes; freshwater lakes and ponds; rivers	Worldwide
		Great Egret	*Ardea alba*	Aquatic predator	Partial migrant	Freshwater/saltwater /brackish marshes; coastal sand beaches/mudflats	Worldwide
		Green Heron	*Butorides virescens*	Aquatic predator	Partial migrant	Freshwater lakes, ponds, and marshes	United States to Ecuador; West Indies
		Striated Heron	*Butorides striata*	Aquatic predator	Resident	Freshwater/saltwater /brackish marshes; mangrove forests	Worldwide
	**Pelecanidae (Pelicans) ***	Brown Pelican	*Pelecanus occidentalis*	Aquatic predator	Partial migrant	Coastal waters	United States to Peru; West Indies
**Podicipediformes ***	**Podicipedidae (Grebes) ***	Eared Grebe	*Podiceps nigricollis*	Aquatic predator	Partial migrant	Freshwater lakes, ponds, and marshes	Worldwide
		Least Grebe	*Tachybaptus dominicus*	Aquatic predator	Resident	Freshwater marshes, lakes, and ponds	Texas, USA to Uruguay
		Pied-billed Grebe	*Podilymbus podiceps*	Aquatic predator	Resident	Freshwater lakes, ponds, and marshes	Canada to Argentina; West Indies
		White-tufted Grebe	*Rollandia rolland*	Aquatic predator	Resident	Freshwater lakes and ponds	Peru to Argentina
**Suliformes**	**Anhingidae (Anhingas) ***	Anhinga	*Anhinga anhinga*	Aquatic predator	Resident	Freshwater lakes and ponds; rivers	United States to Uruguay
	**Fregatidae (Frigatebirds)**	Magnificent Frigatebird	*Fregata magnificens*	Aquatic predator	Resident	Coastal waters; pelagic waters; mangrove forest	United States to Uruguay; Cape Verde
	**Phalacrocoracidae (Cormorants and Shags) ***	Neotropic Cormorant	*Nannopterum brasilianum*	Aquatic predator	Resident	Coastal waters; freshwater lakes and ponds; rivers	Texas, USA to Chile; Cuba
	**Sulidae (Boobies and Gannets) ***	Blue-footed Booby	*Sula nebouxii*	Aquatic predator	Resident	Coastal waters; pelagic waters	Mexico to Peru
		Brown Booby	*Sula leucogaster*	Aquatic predator	Resident	Coastal waters; pelagic waters	Worldwide
